# Chronological age range estimation of cervical vertebral maturation using Baccetti method: a systematic review and meta-analysis

**DOI:** 10.1093/ejo/cjac009

**Published:** 2022-03-08

**Authors:** Maria Inês Magalhães, Vanessa Machado, Paulo Mascarenhas, João Botelho, José João Mendes, Ana Sintra Delgado

**Affiliations:** Clinical Research Unit (CRU), Centro de Investigação Interdisciplinar Egas Moniz (CiiEM), Egas Moniz—Cooperativa de Ensino Superior, Caparica, Almada, Portugal; Clinical Research Unit (CRU), Centro de Investigação Interdisciplinar Egas Moniz (CiiEM), Egas Moniz—Cooperativa de Ensino Superior, Caparica, Almada, Portugal; Orthodontic Department, Egas Moniz Dental Clinic (EMDC), Egas Moniz—Cooperativa de Ensino Superior, Caparica, Almada, Portugal; Evidence-Based Hub, CiiEM, Egas Moniz—Cooperativa de Ensino Superior, Caparica, Almada, Portugal; Evidence-Based Hub, CiiEM, Egas Moniz—Cooperativa de Ensino Superior, Caparica, Almada, Portugal; Clinical Research Unit (CRU), Centro de Investigação Interdisciplinar Egas Moniz (CiiEM), Egas Moniz—Cooperativa de Ensino Superior, Caparica, Almada, Portugal; Evidence-Based Hub, CiiEM, Egas Moniz—Cooperativa de Ensino Superior, Caparica, Almada, Portugal; Clinical Research Unit (CRU), Centro de Investigação Interdisciplinar Egas Moniz (CiiEM), Egas Moniz—Cooperativa de Ensino Superior, Caparica, Almada, Portugal; Evidence-Based Hub, CiiEM, Egas Moniz—Cooperativa de Ensino Superior, Caparica, Almada, Portugal; Clinical Research Unit (CRU), Centro de Investigação Interdisciplinar Egas Moniz (CiiEM), Egas Moniz—Cooperativa de Ensino Superior, Caparica, Almada, Portugal; Orthodontic Department, Egas Moniz Dental Clinic (EMDC), Egas Moniz—Cooperativa de Ensino Superior, Caparica, Almada, Portugal

## Abstract

**Background:**

The timing of growth is a key factor for correct orthodontic treatment planning. Cervical vertebrae maturation (CVM) is no exception, although the reported chronological ages vary in the literature.

**Objective:**

We aimed to estimate the average chronological age for each Baccetti’s CVM staging.

**Search methods:**

Search on MEDLINE-PubMed, Scopus, LILACS, Google Scholar, Cochrane Central Register of Controlled Trials (CENTRAL) was conducted until July 2021. The review was performed according to Preferred Reporting Items for Systematic Reviews and Meta-Analyses (PRISMA) guidelines.

**Selection criteria:**

Observational or interventional studies reporting chronological age classified through Baccetti’s CVM method were included.

**Data collection and analysis:**

Methodological quality was assessed, and pooled estimates were carried out through random-effects meta-analysis of single means. The impact of sex and continent were also investigated through subgroup analyses.

**Results:**

Forty-one studies were included (9867 participants, 4151 men, and 5716 women). The average chronological age was 9.7 years old (95% confidence interval [CI]: 9.4 to 10.1) in CS1, 10.8 years old (95% CI: 10.5 to 11.1) in CS2, 12.0 years old (95% CI: 11.7 to 12.2) in CS3, 13.4 years old (95% CI: 13.2 to 13.6) in CS4, 14.7 years old (95% CI: 14.4 to 15.1) in CS5, and 15.8 years old (95% CI: 15.3 to 16.3) in CS6. A significant difference was found between the sexes in all CVM stages. We also found significant differences across continents.

**Conclusions:**

For each CVM staging a chronological age range was successfully estimated. Girls presented an earlier skeletal maturation compared to boys. The skeletal maturation differs also according to continents, except for CMV stage 1, pointing to the need for personalized ranges according to each region.

**Registration:**

Registration number: PROSPERO: CRD42021225422

## Introduction

The time of intervention in orthodontics is a key factor during diagnosis since the growth of patients has a huge impact on the treatment plan and outcomes (1, 2). Despite chronological age can guide the treatment timing, there is a recognizable individual variation on development stages that might lead orthodontists to look to other methods to assess maturation and predict facial growth (3, 4). Individual skeletal maturation is an exceptionally diverse variable as chronological age and skeletal age often do not match (5–7).

The determination of timing for growth and maturation in growing patients has been the subject of immense research because chronological age presents a moderate level of prediction of skeletal maturation (8, 9). Among these methods are sexual maturation (10, 11), dental eruption and/or calcification stages (12–14), hand-wrist maturation (HWM) (15), cervical vertebral maturation (CVM) (1, 16–18), and biological biomarkers (19, 20).

The CVM index is an effective and practical method for growth rate estimation using morphological characteristics of the second, third, and fourth cervical vertebrae available in lateral cephalometric radiographs (1). Cephalometry is a standard radiograph in orthodontic diagnosis (21, 22), therefore, patients do not require additional exposure to radiation (18). Over the years, three systematic reviews have estimated that the CVM method presents high agreement levels with the HWM method (22–24), although the opposite has also been exhibited (25). All in all, Ferrillo *et al*. (24) have shown the most used CVM method is Baccetti’s case definition (1) with Hassel and Farman case definition (17) to follow as the second.

Nevertheless, the estimation of age ranges for each CVM stage has never been conducted in a systematic manner. These estimations would be of great clinical interest because they can add potentially useful reference values for orthodontic diagnosis. Furthermore, the evolution of our species is not immutable as a result of inter-racial/inter-ethnic mixing previously discussed (26) making this research continuously needed.

Herein, this systematic review comprehensively estimates the age range associated with each CVM stage based on Baccetti’s method (1). We further investigated the impact of sex and geographical location on each CVM stage age range. During the preparation of this systematic review, we sought to answer the following questions: (1) ‘What is the chronological age associated with each Baccetti’s CVM stage?’; (2) ‘For each Baccetti’s CVM stage, is there a significant sex-based difference on chronological age?’. For the first question, our alternative hypothesis is that it is possible to estimate average age ranges for each stage. For the second question, our alternative hypothesis is the existence of a sex-based difference in the chronological age of each CVM stage.

## Materials and methods

### Protocol and registration

This systematic review protocol was defined *a priori* and was registered at the National Institute for Health Research PROSPERO, International Prospective Register of Systematic Review (http://www.crd.york.ac.uk/PROSPERO, ID Number: CRD42021225422). We conducted this review according to the Preferred Reporting Items for Systematic Reviews and Meta-Analyses (PRISMA) guidelines (27).

### Focused question and eligibility criteria

We developed a protocol to answer the following PECO, questions:

1. ‘What is the chronological age associated with each Baccetti’s CVM stage?’ and
2. ‘For each Baccetti’s CVM stage, is there a significant sex-based difference on chronological age?’.

Each question had the respective statements as follows:

1. Children, adolescents, and young adults (Population, P); Lateral cephalometric X-ray or cranium Cone Beam Computerized Tomography (CBCT) (Exposure, E); Chronological age (Comparison, C); Chronological age classified into CVM stages (Outcome, O).
2. Female children, adolescents, and young adults (Population, P); Lateral cephalometric X-ray or CBCT of the cranium (Exposure, E); Male Children, adolescents, and young adults (Comparison, C); Chronological age classified into CVM stages (Outcome, O).

Observational studies (case-control, cross-sectional. and longitudinal) or interventional studies (randomized clinical trials [RCTs] and non-RCTs) in otherwise healthy humans assessing chronological age related with Baccetti’s CVM method on lateral cephalometric radiographs X-ray or CBCT of cranium were eligible for inclusion. We decided to include both RCT and non-RCT studies because restricting to randomized studies would have given an incomplete summary of CVM data. We restricted studies that have used Baccetti’s method given this is the most reported method in the literature (24).

Non-human studies (animal studies or *in vitro* studies), non-original studies (reviews, author responses, comments) or secondary research (systematic review and meta-analysis), case reports or case series, thesis, book chapters, editorials, conference papers, meeting abstracts, and patents were excluded.

### Information sources search and study selection

To establish potentially relevant studies reporting data related to CVM methods and chronological age, we developed detailed search strategies for each database. MEDLINE (via PubMed), Scopus, LILACS, Google Scholar, Cochrane Central Register of Controlled Trials (CENTRAL) databases were searched up to July 2021. Our PubMed search strategy was based on the algorithm: ‘Cervical vertebral maturation’ OR ‘Baccetti’s method’ OR ‘chronological age’. Also, grey literature was searched through http://www.opengrey.eu. No restrictions were applied regarding the year of publication or language. Study selection was assessed independently by two independent authors (MIM and VM), who assessed the titles and/or abstracts of retrieved articles. Any study classified as potentially eligible was screened by the reviewers. Any disagreements were verified and resolved by discussion with a third author (ASD). Inter-examiner reproducibility was calculated following full-text assessment via kappa statistics.

### Data extraction process and data items

We used an electronic table to record patient and study characteristics: first author’s name, project funding, location of the study, year of publication, design study, records years, radiographic method, patients’ characteristics (total number of participants and by sex, mean chronological age), CVM Baccetti’s method (1). All data were extracted independently by two authors (MIM and VM), and any disagreements were resolved by discussion with a third author (ASD). Corresponding authors of studies were contacted if there was missing information or additional clarifications.

### Risk of bias in individual studies

Two researchers (MIM and VM) independently assessed the methodological quality of the included studies, following the quality assessment modified from the Strengthening the Reporting of Observational Studies in Epidemiology (STROBE) (28), Standards for the Reporting of Diagnostic Accuracy Studies (STARD) (29). This adaptation of the assessment tool was previously published in a systematic review (25). Each item was scored using a two-point scale: 0—*not reported or reported inadequately*; and 1—*reported and adequate*. Studies with 12–11 points were considered to be of high quality, studies with 7–10 were of medium quality and studies with 0–6 points were of low quality. Discussion resolved the disagreements between the review authors (MIM and VM) over the quality assessment in any studies, with the involvement of a third review author where necessary (ASD).

### Summary measures and synthesis of results

For continuous data, mean values and standard deviations (SD) were collected from each article to a predefined table prepared to calculate the quantity of data. Studies that reported median and interquartile range, mean and SD were converted following Hozo’s formula (30). The random-effects of single means meta-analysis and forest plots were calculated in R version 3.4.1 (R Studio Team 2018) using the ‘meta’ package, using DerSimonian-Laird random-effects meta-analysis (31). To assess sources of heterogeneity, meta-regression analysis was conducted for each sex. We assess statistical heterogeneity using *I*^2^ index and Cochrane’s *Q* statistic (*P* < 0.1) (32). Chi-square (*χ*^2^) test calculated overall homogeneity (32). All tests were two-tailed with alpha set at 0.05 except for the homogeneity test whose significance level cutoff was 0.10. Overall estimates were reported with 95% confidence interval (CI). For meta-analysis including at least 10, we analysed publication bias (32). Firstly, we started by conducting an a priori sensitivity analysis (in the form of subgroup analyses) comparing the impact of studies with low methodological quality with studies with moderate/high quality. If the results in terms of significance were different, only studies with moderate to high quality were included in this specific analysis.

We assessed the strength of the quality of evidence of included studies by using the Grading of Recommendations Assessment, Development, and Evaluation (GRADE) approach, which analyses the five following domains: (1.) trial design limitations due to risk of bias; (2.) inconsistency of results (or heterogeneity); (3.) indirectness (generalizability); (4.) imprecision (sufficient data); and (5.) the potential for publication bias (33). As recommended by GRADE approach to systematic reviews, quality score was not defined (34).

## Results

### Study selection

**Figure 1:**
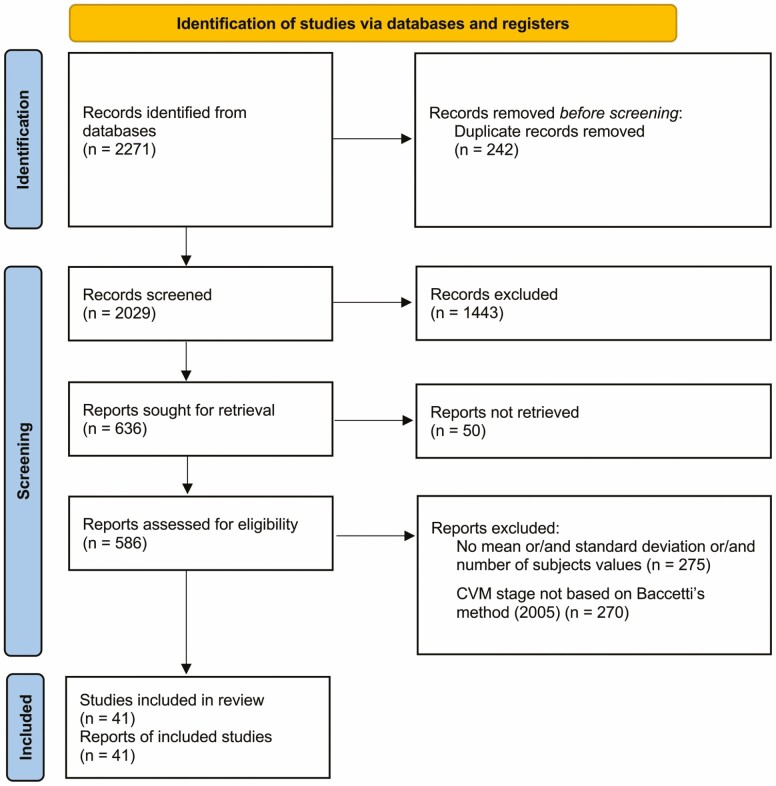
PRISMA flow chart depicting the workflow of the studies selection process based.

The initial database search strategy retrieved 2271 possible relevant articles. Of these, 242 articles were duplicates, and 2029 manuscripts were screened against the eligibility criteria. From a total of 2029 articles, 1443 articles were excluded based on title and/or abstract review. Among these, 586 articles were assessed for full paper review eligibility (detailed reasons for exclusion in Supplementary Table 1). A total of 41 studies were included for qualitative and quantitative analyses (Figure 1). Inter-examiner reliability was calculated and had good results (kappa score = 0.87; 95% CI: 0.79 to 0.95).

### Characteristics of the studies

In this systematic review, 41 studies were included. Overall, 9867 participants were included (4151 men, 5716 women). Eight studies lacked gender information (35–42). Studies were performed in 22 countries across Europe, Asia, America, and Africa (Supplementary Table 2). Notably, no study was performed in Oceania. Only one study used CBCT records to examine the CVM staging (43).

### Methodological quality of the included studies

**Figure 2:**
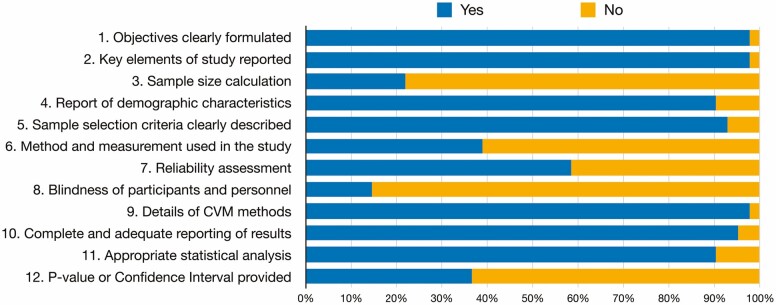
Assessment of the risk of bias in the included studies according to the percentage of the scores attributed to each evaluated study.

Methodological appraisal of the included studies using the STROBE checklist tool is presented in Figure 2 and is detailed in Supplementary Table 3. One study was classified as high quality (11 points) (44), 39 studies had moderate quality (9 scored with 7 points; 15 scored with 8; 9 scored with 9 points; and 6 with 10 points) and one study had low quality (6 points) (37). Good inter-examiner reliability was confirmed at the quality assessment (kappa score = 0.94, 95% CI: 0.84 to 1.00).

Almost all included studies showed clear objectives (*n* = 40, 97.6%), key elements of study design (*n* = 40, 97.6%), and reported demographic characteristics (*n* = 37, 90.2%). The majority carefully described the CVM method (*n* = 40, 97.6%) and reported sample eligibility criteria (*n* = 38, 92.7%). On the opposite, most articles failed on sample size justification (*n* = 32, 78.0%), in the specifications of material used to measurement CVM (*n* = 25, 61.0%), and only six studies reported blindness during CVM measurement (*n* = 6, 14.6%) (Figure 2).

### Mean chronological age for each stage of CVM

Regarding mean chronological age and respective interval for each CVM stage (Table 1), CS1 stage had an estimated mean age of 9.7 years old (95% CI: 9.4 to 10.1, *P* < 0.001, *I*^2^ = 96.8%), CS2 stage had 10.8 years old (95% CI: 10.5 to 11.1, *P* < 0.001, *I*^2^ = 92.0%), CS3 stage had 12.0 years old (95% CI: 11.7 to 12.2, *P* < 0.001, *I*^2^ = 94.3%), CS4 stage had 13.4 years old (95% CI: 13.2 to 13.6, *P* < 0.001, *I*^2^ = 93.6%), CS5 stage had 14.7 years old (95% CI: 14.4 to 15.1, *P* < 0.001, *I*^2^ = 96.5%), and CS6 stage had 15.8 years old (95% CI: 15.3 to 16.3, *P* < 0.001, *I*^2^ = 97.6%).

**Table 1:** Estimates for chronological age as per each cervical vertebrae maturation stage for females and males

	Total					Females					Males					Females vs. males			
CS	*n*	Age mean (years) (95% CI)	*I* ^2^ (%)	*P*-value	Egger test	*n*	Age mean (years) (95% CI)	*I* ^2^ (%)	*P*-value	Egger test	*n*	Age mean (years) (95% CI)	I^2^ (%)	*P*-value	Egger test	*n*	SMD (95% CI)	*I* ^2^ (%)	*P*-value
1	30	9.7 (9.4 to 10.1)	96.8	**<0.0001**	0.2500	24	9.4 (9.0 to 9.7)	95.0	**<0.0001**	0.6867	24	10.2 (9.8 to 10.6)	94.3	**<0.0001**	0.7140	23	0.5 (0.4 to 0.7)	42.5	**<0.0001**
2	33	10.8 (10.5 to 11.1)	92.0		0.9301	26	10.4 (10.1 to 10.7)	88.3		0.6278	27	11.2 (10.9 to 11.6)	91.7		0.8350	25	0.6 (0.4 to 0.7)	52.4	
3	39	12.0 (11.7 to 12.2)	94.3		0.1625	32	11.5 (11.3 to 11.8)	90.1		0.3022	31	12.6 (12.2 to 12.9)	94.0		0.7890	31	0.8 (0.6 to 0.9)	54.7	
4	39	13.4 (13.2 to 13.6)	93.6		0.5143	32	13.1 (12.8 to 13.4)	92.1		0.0788	31	13.9 (13.7 to 14.2)	89.4		**0.0467**	31	0.6 (0.4 to 0.8)	70.5	
5	33	14.7 (14.4 to 15.1)	96.5		0.1121	28	14.5 (14.2 to 14.9)	94.3		0.6719	25	15.3 (14.9 to 15.7)	91.8		0.3678	24	0.6 (0.4 to 0.8)	54.7	
6	30	15.8 (15.3 to 16.3)	97.6		0.0951	25	15.6 (15.0 to 16.2)	96.9		0.2379	21	16.5 (16.0 to 17.0)	95.1		0.0890	21	0.5 (0.4 to 0.7)	0	

Bold-face denotes significance.

CI, confidence interval; CS, cervical stages; *n*, number of included studies; SMD, standardized mean difference.

In what sex concerns, the mean age and respective interval for each CVM stage are presented for both females and males (Table 2). In the comparison between females and males, the estimates pointed to a significant difference in all stages. In other words, females reached the CVM stage about 1 year earlier than the males for CVM stages 1–6 (Table 1). Publication bias was noted only in sub-group analysis in male patients during cervical staging 4 (*P* = 0.0467) (Table 1).

**Table 2:** Estimates comparing cervical vertebrae maturation between continents

CS	Europe			America			Asia			Africa			*P*-value
	n	Mean age (95% CI)	*I* ^2^ (%)	*n*	Mean age (95% CI)	*I* ^2^ (%)	*n*	Mean age (95% CI)	*I* ^2^ (%)	*n*	Mean age (95% CI)	*I* ^2^ (%)	
1	8	9.7 (9.1 to 10.3)	94.3	7	9.0 (8.0 to 10.0)	98.3	13	10.1 (9.5 to 10.6)	97.2	2	9.3 (9.3 to 10.3)	0	0.2815
2	9	10.6 (10.0 to 11.1)	92.3	8	10.2 (9.8 to 10.7)	81.6	14	11.3 (10.9 to 11.7)	93.9	2	10.6 (10.1 to 11.0)	0	**0.0050**
3	13	11.6 (11.2 to 12.0)	92.7	9	11.7 (11.2 to 12.2)	94.2	15	12.5 (12.1 to 12.8)	92.9	2	12.2 (11.1 to 13.3)	74.8	**0.0026**
4	12	12.8 (12.5 to 13.1)	82.6	9	13.3 (12.8 to 13.8)	93.5	16	13.8 (13.5 to 14.1)	92.6	2	13.7 (13.3 to 14.0)	0	**<0.0001**
5	9	13.8 (13.2 to 14.4)	95.6	8	14.7 (14.2 to 15.3)	93.7	15	15.3 (14.9 to 15.8)	94.3	1	14.3 (12.8 to 15.8)	—	**0.0018**
6	8	15.0 (14.3 to 15.7)	93.6	7	15.7 (14.8 to 16.5)	95.2	13	16.5 (15.8 to 17.1)	97.5	2	14.9 (13.9 to 15.9)	22.9	**0.0122**

Bold-face denotes significance.

CI, confidence interval; CS, cervical stages; *n*, number of included studies.

### Additional analyses

Subgroup analysis demonstrated an impact of geographic localization on CVM staging in overall population (Table 2). Importantly, only CVM stage 1 did not differ between continents in overall population, and specifically in male and female patients (Tables 2–4). Otherwise, in CVM stages 2–6 were significant differences between continents in overall population, being the maturation of cervical vertebrae in Asian participants later than European and American participants (Table 2). Furthermore, the CVM occurs in different ages according to continents and sex (Tables 3–5). The quality of evidence for each CVM stage is presented in Supplementary Table 4.

**Table 3:** Female estimates for chronological age as per cervical vertebrae maturation according to continents

CS	Europe			America			Asia			Africa			*P*-value
	*n*	Mean age (years) (95% CI)	*I* ^2^ (%)	n	Mean age (years) (95% CI)	*I* ^2^ (%)	*n*	Mean age (years) (95% CI)	*I* ^2^ (%)	*n*	Mean age (years) (95% CI)	*I* ^2^ (%)	
1	6	9.5 (8.7 to 10.2)	93.3	4	8.5 (6.9 to 10.0)	97.5	12	9.6 (9.1 to 10.1)	95.4	2	9.5 (8.7 to 10.3)	0	0.5921
2	7	10.3 (9.7 to 10.8)	86.7	6	10.0 (9.4 to 10.6)	86.2	11	10.7 (10.3 to 11.1)	91.9	2	10.1 (9.3 to 10.9)	52.5	0.2054
3	10	11.3 (10.9 to 11.7)	87.8	6	11.0 (10.5 to 11.6)	85.0	14	11.9 (11.5 to 12.2)	91.4	2	11.4 (10.1 to 12.7)	74.1	**0.0350**
4	9	12.5 (12.0 to 13.0)	90.3	6	13.6 (13.2 to 14.0)	93.7	15	13.3 (13.1 to 13.8)	92.6	2	13.3 (13.0 to 13.7)	0	**0.0104**
5	7	13.7 (13.0 to 14.5)	94.6	6	14.6 (14.0 to 15.3)	91.5	13	15.0(14.6 to 15.3)	89.2	2	14.1 (11.7 to 16.5)	75.7	**0.0479**
6	6	14.7 (13.8 to 15.6)	93.2	5	15.8 (14.7 to 16.9)	93.0	12	16.0 (15.3 to 16.8)	96.3	2	14.4 (12.7 to 16.2)	63.8	0.0820

Bold-face denotes significance.

CI, confidence interval; CS, cervical stages; *n*, number of included studies.

**Table 4:** Male estimates for chronological age as per cervical vertebrae maturation according continents

CS	Europe			America			Asia			Africa			*P*-value
	*n*	Mean age (95% CI)	*I* ^2^ (%)	*n*	Mean age (95% CI)	*I* ^2^ (%)	*n*	Mean age (95% CI)	*I* ^2^ (%)	*n*	Mean age (95% CI)	*I* ^2^ (%)	
1	6	10.4 (9.7 to 11.1)	88.8	5	9.2 (7.9 to 10.4)	96.3	11	10.4 (9.7 to 11.1)	88.8	2	10.0 (9.4 to 10.5)	0	0.1855
2	7	11.0 (10.5 to 11.5)	83.5	6	10.5 (9.8 to 11.2)	77.6	12	11.7 (11.2 to 12.3)	95.1	2	11.0 (9.7 to 12.3)	62.8	0.0746
3	10	12.1 (11.6 to 12.6)	88.8	6	12.0 (11.1 to 12.9)	85.2	13	13.1 (12.6 to 13.5)	92.7	2	13.3 (12.9 to 13.8)	0	**0.0011**
4	9	13.2 (12.9 to 13.6)	52.8	6	14.0 (13.3 to 14.8)	92.6	14	14.3 (13.9 to 14.6)	88.7	2	14.4 (13.6 to 15.2)	61.2	**<0.0001**
5	7	14.6 (13.9 to 15.3)	87.7	6	15.3 (14.7 to 15.9)	91.4	11	15.7 (15.1 to 16.3)	90.4	1	15.8 (14.7 to 16.8)	—	0.0995
6	5	16.0 (14.8 to 17.1)	91.8	4	16.6 (15.8 to 17.5)	93.5	10	16.8 (16.3 to 17.4)	94.4	2	15.6 (14.9 to 16.2)	0	**0.0334**

Bold-face denotes significance.

CI, confidence interval; CS, cervical stages; *n*, number of included studies.

**Table 5:** Estimates comparing cervical vertebrae maturation between females and males by continents

CS	Europe			America			Asia			Africa			*P*-value
	n	Females vs. males SMD (95% CI)	*I* ^2^ (%)	*n*	Females vs. males SMD (95% CI)	*I* ^2^ (%)	*n*	Females vs. males SMD (95% CI)	*I* ^2^ (%)	*n*	Females vs. males SMD (95% CI)	*I* ^2^ (%)	
1	6	0.5 (0.2 to 0.7)	20.7	4	0.5 (0.2 to 0.8)	0	11	0.6 (0.4 to 0.9)	64.9	2	0.3 (−0.2 to 0.8)	0	0.6275
2	7	0.5 (0.2 to 0.8)	29.8	6	0.5 (0.2 to 0.8)	0	10	0.7 (0.3 to 1.1)	75.8	2	0.4 (−0.7 to 1.5)	56.9	0.7684
3	10	0.6 (0.4 to 0.8)	22.3	6	0.8 (0.5 to 1.0)	0	13	0.9 (0.6 to 1.2)	73.8	2	1.0 (0.4 to 1.6)	0	0.2696
4	9	0.4 (0.1 to 0.7)	69.2	6	0.6 (0.2 to 0.9)	61.7	14	0.6 (0.3 to 1.0)	75.5	2	0.9 (−0.2 to 2.0)	70.3	0.7345
5	7	0.5 (0.3 to 0.8)	36.8	6	0.5 (0.2 to 0.9)	41.4	10	0.5 (0.1 to 0.9)	70.9	1	1.3 (0.1 to 2.6)	—	0.6810
6	5	0.6 (0.3 to 0.9)	0	4	0.4 ( to 0.1 to 0.9)	0	10	0.5 (0.3 to 0.7)	0	2	0.7 (−0.4 to 1.8)	8.3	0.9583

CI, confidence interval; CS, cervical stages; *n*, number of included studies; SMD, standardized mean difference.

## Discussion

### Summary of the main results

Overall, the present systematic review reports age ranges for each Baccetti’s CVM stage. Girls presented earlier maturation rates for each stage, at approximately 1 year earlier than boys. Although stage 1 is consistently alike worldwide, there is some geographic variability for the remaining CVM stages. Despite the majority did not present significant differences or geographic localization, the estimates for American and European subgroups present earlier growth peaks of pubertal growth than African and Asian subgroups, for the overall population and in both female and male patients.

### Quality of the evidence and potential biases in the review process

In the present systematic review, there are some limitations worth mentioning in the included studies. Age range estimates produced by our analyses included mostly unrepresentative studies, and therefore the results cannot be extrapolated worldwide. Additionally, most studies lacked an appropriate sample size calculation, intra- and inter-examiner calibration, and examiners blinding, and these items should be accounted for in future studies. Also, one based the CVM assessment on CBCT images (43). Lateral cephalometric radiograph is a two-dimensional image with several drawbacks, namely lack of perspective, imaging artefacts, variations in magnification, information voids, and head position errors. Contrarily, CBCT allows three-dimensional representation of craniofacial structures with higher reproducibility of measurements compared with conventional cephalometric radiographs, however, rotational errors may occur leading to overestimation of CVM (45). Therefore, conventional two-dimensional cephalometric radiographs must be used, or the CVM staging criteria must be re-evaluated and adjusted to CBCT structures. Also, although we had assessed the impact of continents on CVM, we were unable to explore the possible role of genetic and lifestyle determinants on biological age. Therefore, further studies might search the impact of such factors on growth peak and CVM staging.

To our knowledge, this is the first systematic review proposing age ranges for each CVM staging and exploring the impact of sex and geographic location. These estimations were established upon an evidenced-based, systematic, and strict protocol. In addition, we performed a robust and extensive search to include all relevant evidence and possible confounding factors.

### Agreements and disagreements with studies and clinical relevance

Human development often takes place in a non-linear rhythm, with more than 80% of individuals manifesting such a non-linear pattern, and this is particularly clear during a peak growth (46). The efficacy and effectiveness of orthopaedic therapies are exponentially increased during adolescent growth spurt (47, 48), yet to recognize growth spurts clinicians require the aid of maturation assessment strategies. To make this maturation indexes tangible for the common clinical practice, research has been presenting age ranges to facilitate the interpretation and understanding of clinicians. As such, the chronological age of patients gained some importance.

Chronological age is key factor to consider in orthodontics that not only shapes one’s identity but also is easy to obtain. In contrast, CVM assessment requires a more complex process, requiring radiation exposure to estimate the degree of maturation. When comparing both, similar error variation has been reported to both maturation and timing of peak growth estimation in both sexes (4). Yet, research is not consensual because chronological age has been defined either as the best predictor of skeletal growth and maturation (49), or one not so reliable (8, 9). However, proposing age intervals, in our perspective, has contributed to increase treatment predictability in orthodontics.

For the first time, this evidence-based study proposed estimated age range for each Baccetti’s CVM stage (1). This may contribute to a more consistent orthodontic treatment planning, timing, and clinical management with the patient’s chronological age as an indicator of biological age.

The proposed age ranges will contribute to increase the awareness of clinicians towards the best timing to deliver a particular treatment. Yet the reader must bear in mind that these intervals may be influenced by a multifactorial source, such as biological sex, nutrition, ethnicity, genetics, and socioeconomic status (50, 51). Biological sex is recognized to play a critical role in timing of growth, and therefore, the observed age difference between females and males is not surprising. Female skeletal maturation occurs at an earlier time than males, and this aligns with our estimates, and this may be due to the different hormonal plethora between sexes (52–55).

Overnutrition is another detrimental factor for the timing of puberty (56). Overweight individuals have twice the risk to present early maturation (57), and a strong influence of body mass index (BMI) at the time of puberty (age at peak height velocity) during childhood and adolescence was reported (58, 59). Therefore, a higher BMI gain in childhood (2 and 8 years of age) was related to an earlier onset of puberty, although this is still a matter of debate (60, 61). Serum leptin has been proposed to be responsible for this association, as their serum levels gradually rise prior to puberty in adolescents, suggesting a threshold effect (62, 63). Nevertheless, a serum leptin spike has been reported in shorter or thinner children without any sudden weight gain, questioning this biological marker and the need for more studies in alternative hallmarks (64, 65).

In a recent artificial intelligence (AI)-based study, CVM assessment was proposed to minimize bias between examiners (66). In this experimental work, the determined growth-development period and sex had a satisfactory result. The highest sensitivity and specificity values were found on CVM staging 3, 4, and 5 due to the evidence of formal changes at these stages (66). In the future, more AI studies will allow a full automatic decision regarding this method of skeletal maturation (66).

As such, craniofacial growth reflects a complex and multifactorial interplay. Therefore, in the future, a multifactorial approach with both biological and computerized sciences should be developed to facilitate facial examination and to promote a better understanding, with a personalized localization of areas and tissues involved in normal and abnormal processes. This increasing knowledge should hone our orthodontic diagnosis and therapeutics in the foreseeable future.

## Conclusion

An age range was successfully estimated for each Baccetti CVM stage. Girls were confirmed to present an earlier CVM compared to boys. Cervical maturation differs also according to continents, except for CMV stage 1, pointing to the need for personalized ranges according to each region.

## Supplementary Material

cjac009_suppl_Supplementary_Material

## Data Availability

All data generated or analysed during this study are included in this published article (and its supplementary information files).

## Data availability

All data generated or analysed during this study are included in this published article (and its supplementary information files).
