# Supplementary material for: Chronological age range estimation of cervical vertebral maturation using Baccetti method: a systematic review and meta-analysis
Source: Eur J Orthod. 2022 Mar 8;44(5):548–55. doi: 10.1093/ejo/cjac009 (PMC9486881; doi:10.1093/ejo/cjac009)
Supplement: cjac009_suppl_Supplementary_Material [file cjac009_suppl_supplementary_material.docx]

**Online Supplemental Information**

Summary

[Supplementary Table 1. List of potentially relevant studies not included in the systematic review, along with the reasons for exclusion. 2](#_Toc93563358)

[Supplementary Table 2. Characteristics of included studies. 39](#_Toc93563359)

[Supplementary Table 3. Quality assessment of Selected Full Text Article. 44](#_Toc93563360)

[Supplementary Table 4. Grading of Recommendations Assessment, Development and Evaluation analyses. 46](#_Toc93563361)

Supplementary Table 1. List of potentially relevant studies not included in the systematic review, along with the reasons for exclusion.

| **Year** | **Reference** | **Exclusion reason** |
| --- | --- | --- |
| 1988 | O'Reilly, M. T., & Yanniello, G. J. (1988). Mandibular growth changes and maturation of cervical vertebrae--a longitudinal cephalometric study. *The Angle orthodontist, 58*(2), 179–184. | CVM stage not based on Baccetti’s method (2005) |
| 1990 | Lee, J. H., & Yang, W. S. (1990). A study on the degrees of skeletal maturity of cervical vertebrae and hand-and-wrist in skeletal Class III malocclusions. *The Korean Journal of Orthodontics, 20*(1), 157-168. | CVM stage not based on Baccetti’s method (2005) |
| 1991 | Hellsing E. (1991). Cervical vertebral dimensions in 8-, 11-, and 15-year-old children. *Acta odontologica Scandinavica, 49*(4), 207–213. https://doi.org/10.3109/00016359109005909 | CVM stage not based on Baccetti’s method (2005) |
| 1992 | Solow, B., & Siersbaek-Nielsen, S. (1992). Cervical and craniocervical posture as predictors of craniofacial growth. *American journal of orthodontics and dentofacial orthopedics, 101*(5), 449–458. https://doi.org/10.1016/0889-5406(92)70119-u | No mean or/and standard deviation or/and number of subjects values |
| 1993 | Kopecky, G. .R., Fishman, L. S. (1993). Timing of cervical headgear treatment based on skeletal maturation. American Journal of Orthodontics and Dentofacial Orthopedics , 104(2), 162–169. | CVM stage not based on Baccetti’s method (2005) |
| 1993 | Swischuk, L. E., Swischuk, P. N., & John, S. D. (1993). Wedging of C-3 in infants and children: usually a normal finding and not a fracture. *Radiology, 188*(2), 523–526. https://doi.org/10.1148/radiology.188.2.8327708 | CVM stage not based on Baccetti’s method (2005) |
| 1995 | Hassel, B., & Farman, A. G. (1995). Skeletal maturation evaluation using cervical vertebrae. *American journal of orthodontics and dentofacial orthopedics, 107*(1), 58–66. https://doi.org/10.1016/s0889-5406(95)70157-5 | CVM stage not based on Baccetti’s method (2005) |
| 1997 | Bae, J. H., Park, H. S., & Sung, J. H. (1997). Maturation of cervical vertebrae and mandibular growth changes. The Korean Journal of Orthodontics, 27(3), 481-492. | CVM stage not based on Baccetti’s method (2005) |
| 1998 | García-Fernandez, P., Torre, H., Flores, L., & Rea, J. (1998). The cervical vertebrae as maturational indicators. Journal of clinical orthodontics : JCO, 32(4), 221–225. | CVM stage not based on Baccetti’s method (2005) |
| 2000 | Franchi, L., Baccetti, T., & McNamara, J. A., Jr (2000). Mandibular growth as related to cervical vertebral maturation and body height. *American journal of orthodontics and dentofacial orthopedics, 118*(3), 335–340. https://doi.org/10.1067/mod.2000.107009 | CVM stage not based on Baccetti’s method (2005) |
| 2000 | Baccetti, T., Franchi, L., Toth, L. R., & McNamara, J. A., Jr (2000). Treatment timing for Twin-block therapy. *American journal of orthodontics and dentofacial orthopedics, 118*(2), 159–170. https://doi.org/10.1067/mod.2000.105571 | No mean or/and standard deviation or/and number of subjects values |
| 2001 | Franchi, L., Baccetti, T., & McNamara, J. A., Jr (2001). Thin-plate spline analysis of mandibular growth. *The Angle orthodontist, 71*(2), 83–89. https://doi.org/10.1043/0003-3219(2001)071<0083:TPSAOM>2.0.CO;2 | CVM stage not based on Baccetti’s method (2005) |
| 2001 | Chang, H. P., Liao, C. H., Yang, Y. H., Chang, H. F., & Chen, K. C. (2001). Correlation of cervical vertebra maturation with hand-wrist maturation in children. *The Kaohsiung journal of medical sciences, 17*(1), 29–35. | CVM stage not based on Baccetti’s method (2005) |
| 2001 | Baccetti, T., Franchi, L., Cameron, C. G., & McNamara, J. A. (2001). Treatment timing for rapid maxillary expansion. *The Angle orthodontist, 71*(5), 343–350. https://doi.org/10.1043/0003-3219(2001)071<0343:TTFRME>2.0.CO;2 | No mean or/and standard deviation or/and number of subjects values |
| 2002 | Mito, T., Sato, K., & Mitani, H. (2002). Cervical vertebral bone age in girls. *American journal of orthodontics and dentofacial orthopedics 122*(4), 380–385. https://doi.org/10.1067/mod.2002.126896 | CVM stage not based on Baccetti’s method (2005) |
| 2002 | Baccetti, T., Franchi, L., & McNamara, J. A. (2002). An improved version of the cervical vertebral maturation (CVM) method for the assessment of mandibular growth. *The Angle orthodontist, 72*(4), 316–323. | CVM stage not based on Baccetti’s method (2005) |
| 2002 | San-Román, P., Palma, J. C., Oteo, M. D., & Nevado, E. (2002). Skeletal maturation determined by cervical vertebrae development. *European journal of orthodontics, 24*(3), 303–311. https://doi.org/10.1093/ejo/24.3.303 | CVM stage not based on Baccetti’s method (2005) |
| 2002 | Al-Bustani, A. I., Ismail, A. M., & Al-Izzi, M. K. (2002). The Dental Maturation as an Indicator for the Pubertal Growth Estimation. *Journal of the College of Dentistry, 13, 42-54.* | No mean or/and standard deviation or/and number of subjects values |
| 2003 | Mito, T., Sato, K., & Mitani, H. (2003). Predicting mandibular growth potential with cervical vertebral bone age. *American journal of orthodontics and dentofacial orthopedics, 124*(2), 173–177. https://doi.org/10.1016/s0889-5406(03)00401-3 | CVM stage not based on Baccetti’s method (2005) |
| 2003 | Westwood, P. V., McNamara, J. A., Jr, Baccetti, T., Franchi, L., & Sarver, D. M. (2003). Long-term effects of Class III treatment with rapid maxillary expansion and facemask therapy followed by fixed appliances. American journal of orthodontics and dentofacial orthopedics, 123(3), 306–320. https://doi.org/10.1067/mod.2003.44 | CVM stage not based on Baccetti’s method (2005) |
| 2003 | Faltin, K. J., Faltin, R. M., Baccetti, T., Franchi, L., Ghiozzi, B., & McNamara, J. A., Jr (2003). Long-term effectiveness and treatment timing for Bionator therapy. *The Angle orthodontist, 73*(3), 221–230. https://doi.org/10.1043/0003-3219(2003)073<0221:LEATTF>2.0.CO;2 | No mean or/and standard deviation or/and number of subjects values |
| 2003 | Madhu, S., Hegde, A. M., & Munshi, A. K. (2003). The developmental stages of the middle phalanx of the third finger (MP3): a sole indicator in assessing the skeletal maturity?. *The Journal of clinical pediatric dentistry, 27*(2), 149–156. https://doi.org/10.17796/jcpd.27.2.qtj75rg3714l5543 | CVM stage not based on Baccetti’s method (2005) |
| 2003 | Canali, L., Brucker, M. R., & Lima, E. M. S. (2003). Avaliação da maturação esquelética das vértebras cervicais através de teleradiografia de perfil, *Revista Odonto Ciência, 18*(40), 127-137. | CVM stage not based on Baccetti’s method (2005) |
| 2004 | Chen, F., Terada, K., & Hanada, K. (2004). A new method of predicting mandibular length increment on the basis of cervical vertebrae. *The Angle orthodontist, 74*(5), 630–634. https://doi.org/10.1043/0003-3219(2004)074<0630:ANMOPM>2.0.CO;2 | CVM stage not based on Baccetti’s method (2005) |
| 2004 | Baccetti, T., Franchi, L., & McNamara, J. A., Jr (2004). Cephalometric variables predicting the long-term success or failure of combined rapid maxillary expansion and facial mask therapy. *American journal of orthodontics and dentofacial orthopedics, 126*(1), 16–22. https://doi.org/10.1016/j.ajodo.2003.06.010 | CVM stage not based on Baccetti’s method (2005) |
| 2004 | Leonardi, M., Armi, P., Franchi, L., & Baccetti, T. (2004). Two interceptive approaches to palatally displaced canines: a prospective longitudinal study. *The Angle orthodontist, 74*(5), 581–586. https://doi.org/10.1043/0003-3219(2004)074<0581:TIATPD>2.0.CO;2 | CVM stage not based on Baccetti’s method (2005) |
| 2005 | Baccetti,T., Franchi, L., & McNamara J. A. (2005). The Cervical Vertebral Maturation (CVM) Method for the Assessment of Optimal Treatment Timing in Dentofacial Orth*opedic, Seminars in Ortho*dontics, 11(3), 119–129. | No mean or/and standard deviation or/and number of subjects values |
| 2005 | Hedayati, Z., Shahidi, S., & Golabinejad, M. (2005). Evaluation of Degree of Agreement between Cervical Vertebrae Maturation Indicators in lateral Cephalometry and Hand Wrist Radiographs. *Journal of Dentistry, 6*(4), 151-162. | CVM stage not based on Baccetti’s method (2005) |
| 2005 | Seedat, A. K., & Forsberg, C. D. (2005). An evaluation of the third cervical vertebra (C3) as a growth indicator in Black subjects. *Journal of the South African Dental Association, 60*(4), 156–160. | CVM stage not based on Baccetti’s method (2005) |
| 2005 | Chen, F., Terada, K., & Hanada, K. (2005). A special method of predicting mandibular growth potential for Class III malocclusion. *The Angle orthodontist, 75*(2), 191–195. | CVM stage not based on Baccetti’s method (2005) |
| 2005 | El-Shourbagy, E. M. (2005). The effect of Beta Thalassemia on maturation of cervical vertebrae and craniofacial morphology. *Egyptian Orthodontic Journal, 27*, 55-75. | CVM stage not based on Baccetti’s method (2005) |
| 2005 | Marcelino, E., Tavano, O., & Carvalho, I. M. M. (2005). Cervical vertebrae as growth and development estimator in cleft lip/palate patients. *Salusvita, 24(1)*, 21-28. | No mean or/and standard deviation or/and number of subjects values |
| 2005 | Schulz, S. O., McNamara, J. A., Jr, Baccetti, T., & Franchi, L. (2005). Treatment effects of bonded RME and vertical-pull chincup followed by fixed appliance in patients with increased vertical dimension. *American journal of orthodontics and dentofacial orthopedics, 128*(3), 326–336. https://doi.org/10.1016/j.ajodo.2004.03.039 | No mean or/and standard deviation or/and number of subjects values |
| 2005 | Baccetti, T., Reyes, B. C., & McNamara, J. A. (2005). Gender differences in Class III malocclusion. *The Angle orthodontist, 75*(4), 510–520. https://doi.org/10.1043/0003-3219(2005)75[ | No mean or/and standard deviation or/and number of subjects values |
| 2005 | Bicakci, A. A., Agar, U., Sökücü, O., Babacan, H., & Doruk, C. (2005). Nasal airway changes due to rapid maxillary expansion timing. *The Angle orthodontist, 75*(1), 1–6. https://doi.org/10.1043/0003-3219(2005)075<0001:NACDTR>2.0.CO;2 | No mean or/and standard deviation or/and number of subjects values |
| 2006 | Uysal, T., Ramoglu, S. I., Basciftci, F. A., & Sari, Z. (2006). Chronologic age and skeletal maturation of the cervical vertebrae and hand-wrist: is there a relationship?. American journal of orthodontics and dentofacial orthopedics, 130(5), 622–628. https://doi.org/10.1016/j.ajodo.2005.01.031 | CVM stage not based on Baccetti’s method (2005) |
| 2006 | Kama, J. D., Arslan, S. G., Dari, O., & Özer, T. (2006). Erkek Bireylerde Servikal Vertebra Kemik Yaşının Kronolojik ve İskelet Yaş ile Karşılaştırılması. *Dicle Tıp Dergisi, 33*(1), 36–41. | CVM stage not based on Baccetti’s method (2005) |
| 2006 | Lima, K. T. F., Sales, R. D., Soares, E. A., Cruz, H. N., & Soares, R. P. F. (2006). Comparação entre três métodos para a determinação da maturação esquelética. Odontologia clínico-científica, 5(1), 49–55. | CVM stage not based on Baccetti’s method (2005) |
| 2006 | Tafakhori, Z., Shokrizadeh, M., & Fathollahi, M. S. (2006). Relationship between Dental Development and Cervical Vertebrae Development Assessed Using Radiography in an Iranian Population. Journal of Dentomaxillofacial Radiology, Pathology and Surgery, 5(2), 17-23. | CVM stage not based on Baccetti’s method (2005) |
| 2006 | Gandini, P., Mancini, M., & Andreani, F. (2006). A comparison of hand-wrist bone and cervical vertebral analyses in measuring skeletal maturation. *The Angle orthodontist, 76*(6), 984–989. https://doi.org/10.2319/070605-217 | CVM stage not based on Baccetti’s method (2005) |
| 2006 | Grippaudo, C., Garcovich, D., Volpe, G., & Lajolo, C. (2006). Comparative evaluation between cervical vertebral morphology and hand-wrist morphology for skeletal maturation assessment. Minerva stomatologica, 55(5), 271–280. | CVM stage not based on Baccetti’s method (2005) |
| 2006 | Ozer, T., Kama, J. D., & Ozer, S. Y. (2006). A practical method for determining pubertal growth spurt. *American journal of orthodontics and dentofacial orthopedics, 130*(2), 131.e1–131.e1316. https://doi.org/10.1016/j.ajodo.2006.01.019 | No mean or/and standard deviation or/and number of subjects values |
| 2006 | Kamal, M., Ragini, & Goyal, S. (2006). Comparative evaluation of hand wrist radiographs with cervical vertebrae for skeletal maturation in 10-12 years old children. Journal of the Indian Society of Pedodontics and Preventive Dentistry, 24(3), 127–135. https://doi.org/10.4103/0970-4388.27901 | CVM stage not based on Baccetti’s method (2005) |
| 2006 | Santos, E. C., Bertoz, F. A., Arantes, F., Reis, P. M., & de Bertoz, A. P. (2006). Skeletal maturation analysis by morphological evaluation of the cervical vertebrae. The Journal of clinical pediatric dentistry, 30(3), 265–270. https://doi.org/10.17796/jcpd.30.3.f53857h02n3t1022 | CVM stage not based on Baccetti’s method (2005) |
| 2006 | Baccetti, T., Franchi, L., De Toffol, L., Ghiozzi, B., & Cozza, P. (2006). The diagnostic performance of chronologic age in the assessment of skeletal maturity. *Progress in orthodontics, 7*(2), 176–188. | No mean or/and standard deviation or/and number of subjects values |
| 2006 | Damian, M. F., Woitchunas, F. E., Cericato, G. O., Cechinato, F., Moro, G., Massochin, M. E., & Castoldi, F. L. (2006). Análise da confiabilidade e da correlação de dois índices de estimativa da maturação esquelética: índice carpal e índice vertebral. *Revista Dental Press de Ortodontia e Ortopedia Facial, 11*, 110-120. | No mean or/and standard deviation or/and number of subjects values |
| 2006 | Flores-Mir, C., Burgess, C. A., Champney, M., Jensen, R. J., Pitcher, M. R., & Major, P. W. (2006). Correlation of skeletal maturation stages determined by cervical vertebrae and hand-wrist evaluations. *The Angle orthodontist, 76*(1), 1–5. https://doi.org/10.1043/0003-3219(2006)076[0001:COSMSD]2.0.CO;2 | No mean or/and standard deviation or/and number of subjects values |
| 2007 | Caldas, M., Ambrosano, G. M., & Haiter-Neto, F. (2007). Use of cervical vertebral dimensions for assessment of children growth. *Journal of applied oral science, 15*(2), 144–147. https://doi.org/10.1590/s1678-77572007000200014 | CVM stage not based on Baccetti’s method (2005) |
| 2007 | Caldas, M., Ambrosano, G. M., & Haiter Neto, F. (2007). New formula to objectively evaluate skeletal maturation using lateral cephalometric radiographs. *Brazilian oral research, 21*(4), 330–335. https://doi.org/10.1590/s1806-83242007000400009 | CVM stage not based on Baccetti’s method (2005) |
| 2007 | Damian, M. F., Cechinato, F., Molina, R. D., & Woitchunas, F. E. (2007). Relationship between cranial and mandibular growth and the stages of maturation of the cervical vertebrae. Journal of applied oral science, 15(2), 115–119. https://doi.org/10.1590/s1678-77572007000200008 | CVM stage not based on Baccetti’s method (2005) |
| 2007 | Rai, B., & Anand, S. C. (2007). Relationship of different radiograph: Maturity indicator. Advances in Medical and Dental Sciences, 1(1), 15-18. | CVM stage not based on Baccetti’s method (2005) |
| 2007 | Manhães, J. L. R. C., Moraes, M. E. L. D., Moraes, L. C. D., Castilho, J. C. D. M., Varoli, F. P., Oliveira, J. X., & Junqueira, J. L. C. (2007). Maturação óssea das vértebras cervicais correlacionada com a maturação óssea de mão e punho e com a mineralização dentária pelo método radiográfico. Ortodontia, 40(4), 273-280. | CVM stage not based on Baccetti’s method (2005) |
| 2007 | Cericato, G. O., Siviero, D., Woitchunas, F. E., & Damian, M. F. (2007). As vértebras cervicais como instrumento para determinação da idade óssea. Revista Faculdade Odontologica, 12(1), 42–46. | CVM stage not based on Baccetti’s method (2005) |
| 2007 | Baccetti, T., Reyes, B. C., & McNamara, J. A. (2007). Craniofacial changes in Class III malocclusion as related to skeletal and dental maturation. American Journal of Orthodontics and Dentofacial Orthopedics, 132(2), 171.e1–171.e12. https://doi.org/10.1016/j.ajodo.2005.07.031 | No mean or/and standard deviation or/and number of subjects values |
| 2007 | Manhães Júnior, L. R. C., Moraes, M. E. L. de, Moraes, L. C. de, Castilho, J. C. de M., Varoli, F. P., Oliveira, J. X., & Junqueira, J. L. C. (2007). Maturação óssea das vértebras cervicais correlacionada com a maturação óssea de mão e punho e com a mineralização dentária pelo método radiográfico. Ortodontia, 40(4), 273–280. | CVM stage not based on Baccetti’s method (2005) |
| 2007 | Caldas, M. de P., Ambrosano, G. M. B., & Haiter-Neto, F. (2007). Use of cervical vertebral dimensions for assessment of children growth. Journal of Applied Oral Science, 15(2), 144–147. https://doi.org/10.1590/S1678-77572007000200014 | No mean or/and standard deviation or/and number of subjects values |
| 2007 | Caldas, M. de P., Ambrosano, G. M. B., & Haiter Neto, F. (2007). New formula to objectively evaluate skeletal maturation using lateral cephalometric radiographs. Brazilian Oral Research, 21(4), 330–335. https://doi.org/10.1590/S1806-83242007000400009 | CVM stage not based on Baccetti’s method (2005) |
| 2007 | Shi, H., Scarfe, W. C., & Farman, A. G. (2007). Three-dimensional reconstruction of individual cervical vertebrae from cone-beam computed-tomography images. *American journal of orthodontics and dentofacial orthopedics, 131*(3), 426–432. https://doi.org/10.1016/j.ajodo.2005.12.031 | CVM stage not based on Baccetti’s method (2005) |
| 2007 | Wang, Y. Li, H., & Jian-Xi, L. V.(2007). Foundation and analysis of method on the estimating cervical vertebral bone age of the femal children in Tianjin. *Journal of Tianjin Medical University,* 13(3), 343–345. | No mean or/and standard deviation or/and number of subjects values |
| 2007 | Nasssar, A., Hassan, R., Rajion, Z. A., & Khamis, M. F. (2007). The relationship between the stages of cervical vertebral maturation and dental calcification among Malay: A preliminary study. *Malaysian Journal of Medical Sciences, 14,* 257. | CVM stage not based on Baccetti’s method (2005) |
| 2007 | Baccetti, T., Franchi, L., & McNamara Jr, J. A. (2007) Growth in the Untreated Class III Subject. *Seminars in Orthodontics, 13*(3), 130-142. | No mean or/and standard deviation or/and number of subjects values |
| 2007 | Sun, Y., You, Q. L., & Liu, H. H. (2007). Correlation of adolescents' skeletal maturation determined by cervical vertebrae and hand-wrist in Shanghai region. *Shanghai journal of stomatology, 16*(4), 365-369. | CVM stage not based on Baccetti’s method (2005) |
| 2007 | Choi, B. S., Choi, N. K., Kim, S. M., Yang, K. H., & Chung, S. S. (2007). Usefulness of cervical vertebrae maturation stage as a mandibular maturation indicator. The Journal of The Korean Academy of Pediatric Dentistry, 34(4), 551-559. | No mean or/and standard deviation or/and number of subjects values |
| 2007 | Tassi, N. G., Franchi, L., Baccetti, T., & Barbato, E. (2007). Diagnostic performance study on the relationship between the exfoliation of the deciduous second molars and the pubertal growth spurt. *American journal of orthodontics and dentofacial orthopedics, 131*(6), 769–771. https://doi.org/10.1016/j.ajodo.2006.09.039 | No mean or/and standard deviation or/and number of subjects values |
| 2007 | Başaran, G., Ozer, T., & Hamamci, N. (2007). Cervical vertebral and dental maturity in Turkish subjects. American journal of orthodontics and dentofacial orthopedics, 131(4), 447.e13–447.e4.47E20. https://doi.org/10.1016/j.ajodo.2006.08.016 | CVM stage not based on Baccetti’s method (2005) |
| 2007 | Al-Hadlaq, A. D. E. L., Al-Qarni, M., Al-Kahtani, A., & Al-Obaid, A. (2007). Comparative study between hand-wrist method and cervical vertebral maturation method for evaluation of skeletal maturity in Saudi boys. *Pakistan Oral & Dental Journal, 27*(2), 187-192. | CVM stage not based on Baccetti’s method (2005) |
| 2008 | Alkhal, H. A., Wong, R. W., & Rabie, A. B. (2008). Correlation between chronological age, cervical vertebral maturation and Fishman's skeletal maturity indicators in southern Chinese*. The Angle orthodontist, 78*(4), 591–596. https://doi.org/10.2319/0003-3219(2008)078[0591:CBCACV]2.0.CO;2 | No mean or/and standard deviation or/and number of subjects values |
| 2008 | Mourelle, R., Barbería, E., Gallardo, N., & Lucavechi, T. (2008). Correlation between dental maturation and bone growth markers in paediatric patients. European Journal of Paediatric Dentistry, 9(1), 23–29. | CVM stage not based on Baccetti’s method (2005) |
| 2008 | Franchi, L., Baccetti, T., De Toffol, L., Polimeni, A., & Cozza, P. (2008). Phases of the dentition for the assessment of skeletal maturity: a diagnostic performance study. *American journal of orthodontics and dentofacial orthopedics, 133*(3), 395–476.e4762. https://doi.org/10.1016/j.ajodo.2006.02.040 | No mean or/and standard deviation or/and number of subjects values |
| 2008 | Chen, L.-L., Xu, T.-M., Jiang, J.-H., Zhang, X.-Z., & Lin, J.-X. (2008). Quantitative cervical vertebral maturation assessment in adolescents with normal occlusion: A mixed longitudinal study. American Journal of Orthodontics and Dentofacial Orthopedics, 134(6), 720.e1-720.e7. https://doi.org/10.1016/j.ajodo.2008.03.014 | CVM stage not based on Baccetti’s method (2005) |
| 2008 | Fudalej, P., Rothe, L. E., & Bollen, A. M. (2008). Effects of posttreatment skeletal maturity measured with the cervical vertebral maturation method on incisor alignment relapse. *American journal of orthodontics and dentofacial orthopedics, 134*(2), 238–244. https://doi.org/10.1016/j.ajodo.2006.09.060 | CVM stage not based on Baccetti’s method (2005) |
| 2008 | Soegiharto, B. M., Cunningham, S. J., & Moles, D. R. (2008). Skeletal maturation in Indonesian and white children assessed with hand-wrist and cervical vertebrae methods. *American journal of orthodontics and dentofacial orthopedics, 134(*2), 217–226. https://doi.org/10.1016/j.ajodo.2006.07.037 | CVM stage not based on Baccetti’s method (2005) |
| 2008 | Moghadam, I. M., Heravi, F., Maryam, K., & Elah, E. H. (2008). Evaluation of the Correlation of Different Methods in Determining Skeletal Maturation Utilizing Cervical Vertebrae in Lateral Cephalogram. *Journal of Mashhad Dental School, 32*(2), 95-102. | CVM stage not based on Baccetti’s method (2005) |
| 2008 | Gu, Y., & McNamara Jr, A. (2008). Cephalometric Superimpositions: A Comparison of Anatomical and Metallic Implant Methods. *The Angle Orthodontist, 78*(6), 967-976. | No mean or/and standard deviation or/and number of subjects values |
| 2008 | Stahl, F., Baccetti, T., Franchi, L., & McNamara, J. A., Jr (2008). Longitudinal growth changes in untreated subjects with Class II Division 1 malocclusion. *American journal of orthodontics and dentofacial orthopedics, 134*(1), 125–137. https://doi.org/10.1016/j.ajodo.2006.06.028 | No mean or/and standard deviation or/and number of subjects values |
| 2008 | Baccetti, T., Franchi, L., De Lisa, S., & Giuntini, V. (2008). Eruption of the maxillary canines in relation to skeletal maturity. *American journal of orthodontics and dentofacial orthopedics, 13*3(5), 748–751. https://doi.org/10.1016/j.ajodo.2007.10.031 | No mean or/and standard deviation or/and number of subjects values |
| 2008 | Rozira, H., Zainul, A. R., Fadhli, M., & Ahmad, N. (2008). Skeletal maturation of Malay Patients attending orthodontic clinic in hospital Universiti Sains Malaysia. *Malaysian Jornal of Medical Sciences, 15,* 242. | CVM stage not based on Baccetti’s method (2005) |
| 2008 | Al Khal, H. A., Wong, R. W., & Rabie, A. B. (2008). Elimination of hand-wrist radiographs for maturity assessment in children needing orthodontic therapy. *Skeletal radiology, 37*(3), 195–200. https://doi.org/10.1007/s00256-007-0369-4 | No mean or/and standard deviation or/and number of subjects values |
| 2008 | Baccetti, T., Franchi, L., Schulz, S. O., & McNamara, J. A., Jr (2008). Treatment timing for an orthopedic approach to patients with increased vertical dimension. *American journal of orthodontics and dentofacial orthopedics, 133*(1), 58–64. https://doi.org/10.1016/j.ajodo.2006.01.048 | No mean or/and standard deviation or/and number of subjects values |
| 2008 | Lee, K. H., Hwang, Y. I., Kim, Y. J., Park, Y. H., Baek, S. H., & Cha, K. S. (2008). Skeletal maturation associated with the fourth cervical vertebra and menarcheal timing. *Korean Journal of Orthodontics, 38*(1), 52-59. | No mean or/and standard deviation or/and number of subjects values |
| 2008 | Ismail, H. A., Essam, W., Mahmoud, M., & Sheta, A. (2008). Assessment of the skeletal maturation of cervical vertebrae and hand wrist in relation to chronological age among a sample of Egyptian adolescents. *Egyptian Orthodontic Journal, 33*, 1-21. | CVM stage not based on Baccetti’s method (2005) |
| 2008 | Giuntini, V., Franchi, L., Baccetti, T., Mucedero, M., & Cozza, P. (2008). Dentoskeletal changes associated with fixed and removable appliances with a crib in open-bite patients in the mixed dentition. *American journal of orthodontics and dentofacial orthopedics, 13*3(1), 77–80. https://doi.org/10.1016/j.ajodo.2007.07.012 | No mean or/and standard deviation or/and number of subjects values |
| 2008 | Sjahruddin, L. D., Retno, R. H. S., & Ariffin, S. (2008). The cervical vertebral bone maturation of Hb E Beta Thalassemia patietns of Deuteromalay aged 9-14 years. *Journal of Dentistry Indonesia, 13*(1), 134-138. | No mean or/and standard deviation or/and number of subjects values |
| 2008 | Browd, S. R., McIntyre, J. S., & Brockmeyer, D. (2008). Failed age-dependent maturation of the occipital condyle in patients with congenital occipitoatlantal instability and Down syndrome: a preliminary analysis. *Journal of neurosurgery, 2(*5), 359–364. https://doi.org/10.3171/PED.2008.2.11.359 | No mean or/and standard deviation or/and number of subjects values |
| 2008 | Masoud, M., Masoud, I., Kent, R. L., Jr, Gowharji, N., & Cohen, L. E. (2008). Assessing skeletal maturity by using blood spot insulin-like growth factor I (IGF-I) testing*. American journal of orthodontics and dentofacial orthopedics, 134*(2), 209–216. https://doi.org/10.1016/j.ajodo.2006.09.063 | No mean or/and standard deviation or/and number of subjects values |
| 2008 | Samartzis, D., Kalluri, P., Herman, J., Lubicky, J. P., & Shen, F. H. (2008). The Extent of Fusion Within the Congenital Klippel-Feil Segment. *Spine, 33*(15), 1637–1642. doi:10.1097/brs.0b013e31817c0bc2 | No mean or/and standard deviation or/and number of subjects values |
| 2008 | Samartzis, D., Kalluri, P., Herman, J., Lubicky, J. P., & Shen, F. H. (2008). 2008 Young Investigator Award: The Role of Congenitally Fused Cervical Segments Upon the Space Available for the Cord and Associated Symptoms in Klippel-Feil Patients. *Spine, 33*(13), 1442–1450. doi:10.1097/brs.0b013e3181753ca6 | No mean or/and standard deviation or/and number of subjects values |
| 2008 | Rey, D., Angel, D., Oberti, G., & Baccetti, T. (2008). Treatment and posttreatment effects of mandibular cervical headgear followed by fixed appliances in Class III malocclusion*. American Journal of Orthodontics and Dentofacial Orthopedics, 133*(3), 371–378. doi:10.1016/j.ajodo.2006.04.043 | No mean or/and standard deviation or/and number of subjects values |
| 2008 | Dong-gyun, K., Tae-wan, K., Jin, K., Soon-hyun, N., & Hyun-jung, K. (2008). Um estudo sobre a correlação entre o estágio de calcificação dentária e o estágio de maturação óssea. *Journal of The Korean Academy of Pediatric Dentistry, 35*(2), 243-258. | CVM stage not based on Baccetti’s method (2005) |
| 2008 | Moscatiello, V. A. M., Lederman, H., Moscatiello, R. A., Júnior, K. F. & Moscatiello, R. M. (2008). Maturação das vértebras cervicais e sua correlação com a idade óssea da mão e punho como indicadores no tratamento ortodôntico. *Revista Dental Press de ortodontia e ortopedia facial, 13*(4), 92-100. | CVM stage not based on Baccetti’s method (2005) |
| 2008 | Soegiharto, B. M., Moles, D. R., & Cunningham, S. J. (2008). Discriminatory ability of the skeletal maturation index and the cervical vertebrae maturation index in detecting peak pubertal growth in Indonesian and white subjects with receiver operating characteristics analysis. *American journal of orthodontics and dentofacial orthopedics, 134*(2), 227–237. https://doi.org/10.1016/j.ajodo.2006.09.062 | CVM stage not based on Baccetti’s method (2005) |
| 2009 | Vieira, C. L., Oliveira, A. E. F., Ribeiro, C. C. C. & Lima, a. A. S. J. (2009). Relação entre os índices de maturação das vértebras cervicais e os estágios de calcificação dentária. Revista Dental Press de Ortodontia e Ortopedia Facial, 14(2), 45-53. https://dx.doi.org/10.1590/S1415-54192009000200006 | CVM stage not based on Baccetti’s method (2005) |
| 2009 | Cho, S., & Hwang, C. (2009). Skeletal maturation evaluation using mandibular third molar development in adolescents. The Korean Journal of Orthodontics, 39(2), 120–129. | CVM stage not based on Baccetti’s method (2005) |
| 2009 | Wong, R. W., Alkhal, H. A., & Rabie, A. B. (2009). Use of cervical vertebral maturation to determine skeletal age. *American journal of orthodontics and dentofacial orthopedics, 136*(4), 484.e1–485. | No mean or/and standard deviation or/and number of subjects values |
| 2009 | Gabriel, D. B., Southard, K. A., Qian, F., Marshall, S. D., Franciscus, R. G., & Southard, T. E. (2009). Cervical vertebrae maturation method: poor reproducibility. *American journal of orthodontics and dentofacial orthopedics, 136*(4), 478.e1–480. https://doi.org/10.1016/j.ajodo.2007.08.028 | No mean or/and standard deviation or/and number of subjects values |
| 2009 | Chen, L., Lin, J., Xu, T., & Long, X. (2009). The longitudinal sagittal growth changes of maxilla and mandible according to quantitative cervical vertebral maturation. *Journal of Huazhong University of Science and Technology Medical sciences, 29*(2), 251–256. https://doi.org/10.1007/s11596-009-0224-z | CVM stage not based on Baccetti’s method (2005) |
| 2009 | Chatzigianni, A., & Halazonetis, D. J. (2009). Geometric morphometric evaluation of cervical vertebrae shape and its relationship to skeletal maturation. *American journal of orthodontics and dentofacial orthopedics, 136*(4), 481.e1–483. https://doi.org/10.1016/j.ajodo.2009.04.017 | No mean or/and standard deviation or/and number of subjects values |
| 2009 | Wong, R. W. K., Alkhal, H. A., & Rabie, A. B. M. (2009). Editor's Summary and Q&A: Use of cervical vertebral maturation to determine skeletal age. *American Journal of Orthodontics and Dentofacial Orthopedics, 136*(4), 484-485. | No mean or/and standard deviation or/and number of subjects values |
| 2009 | Lee, J. H., Kang, Y. G. Lee, K. S. & Nam, J. (2009). Maturation of cervical vertebrae in relation to menarche. *Korean Journal of Orthodontics, 39*(1), 28-35. | CVM stage not based on Baccetti’s method (2005) |
| 2009 | Baccetti, T., Stahl, F., & McNamara, J. A., Jr (2009). Dentofacial growth changes in subjects with untreated Class II malocclusion from late puberty through young adulthood. *American journal of orthodontics and dentofacial orthopedics, 13*5(2), 148–154. https://doi.org/10.1016/j.ajodo.2007.03.033 | No mean or/and standard deviation or/and number of subjects values |
| 2009 | Stiehl, J., Müller, B., & Dibbets, J. (2009). The development of the cervical vertebrae as an indicator of skeletal maturity: comparison with the classic method of hand-wrist radiograph. *Journal of orofacial orthopedics, 70*(4), 327–335. https://doi.org/10.1007/s00056-009-9918-x | CVM stage not based on Baccetti’s method (2005) |
| 2009 | Vásquez, M. J., Baccetti, T., Franchi, L., & McNamara, J. A., Jr (2009). Dentofacial features of Class II malocclusion associated with maxillary skeletal protrusion: a longitudinal study at the circumpubertal growth period. *American journal of orthodontics and dentofacial orthopedics, 135*(5), 568.e1–569. https://doi.org/10.1016/j.ajodo.2007.05.026 | No mean or/and standard deviation or/and number of subjects values |
| 2009 | Haralambidis, A., Ari-Demirkaya, A., Acar, A., Küçükkeleş, N., Ateş, M., & Ozkaya, S. (2009). Morphologic changes of the nasal cavity induced by rapid maxillary expansion: a study on 3-dimensional computed tomography models. *American journal of orthodontics and dentofacial orthopedics, 136*(6), 815–821. https://doi.org/10.1016/j.ajodo.2008.03.020 | No mean or/and standard deviation or/and number of subjects values |
| 2009 | Malta, L. A., Ortolani, C. F., & Faltin, K. (2009). Quantification of cranial base growth during pubertal growth*. Journal of orthodontics, 36*(4), 229–235. https://doi.org/10.1179/14653120723256 | No mean or/and standard deviation or/and number of subjects values |
| 2010 | Caldas, M., Ambrosano, G. M., & Haiter Neto, F. (2010). Computer-assisted analysis of cervical vertebral bone age using cephalometric radiographs in Brazilian subjects. *Brazilian oral research, 24*(1), 120–126. https://doi.org/10.1590/s1806-83242010000100020 | CVM stage not based on Baccetti’s method (2005) |
| 2010 | Bica C., Pacurar M., Bud. E. (2010). Relations Between Cervical Vertebral Maturation And Chronological Age. In Romanian Journal Oral Rehabilitation, 2(1), 4-7. | No mean or/and standard deviation or/and number of subjects values |
| 2010 | Johnson, B. M., McNamara, J. A., Bandeen, R. L., & Baccetti, T. (2010). Changes in soft tissue nasal widths associated with rapid maxillary expansion in prepubertal and postpubertal subjects. The Angle Orthodontist, 80(6), 995–1001. https://doi.org/10.2319/033110-179.1 | No mean or/and standard deviation or/and number of subjects values |
| 2010 | Chen, L., Liu, J., Xu, T., Long, X., & Lin, J. (2010). Quantitative skeletal evaluation based on cervical vertebral maturation: a longitudinal study of adolescents with normal occlusion. *International journal of oral and maxillofacial surgery, 39*(7), 653–659. https://doi.org/10.1016/j.ijom.2010.03.026 | CVM stage not based on Baccetti’s method (2005) |
| 2010 | Litsas, G., & Ari-Demirkaya, A. (2010). Growth indicators in orthodontic patients. Part 1: comparison of cervical vertebral maturation and hand-wrist skeletal maturation. *European journal of paediatric dentistry, 11*(4), 171–175. | CVM stage not based on Baccetti’s method (2005) |
| 2010 | Chen, L., Liu, J., Xu, T., & Lin, J. (2010). Longitudinal study of relative growth rates of the maxilla and the mandible according to quantitative cervical vertebral maturation. *American journal of orthodontics and dentofacial orthopedics, 137*(6), 736.e1–737. https://doi.org/10.1016/j.ajodo.2009.12.022 | CVM stage not based on Baccetti’s method (2005) |
| 2010 | Bicã, C., Pãcurar, M., & Bud, E. (2010). Relations between cervical vertebral maturation and chronological age.*Romanian Journal of Oral Rehabilitation, 2*(1), 4-7. | No mean or/and standard deviation or/and number of subjects values |
| 2010 | Jaqueira, L. M., Armond, M. C., Pereira, L. J., Alcântara, C. E., & Marques, L. S. (2010). Determining skeletal maturation stage using cervical vertebrae: evaluation of three diagnostic methods. *Brazilian oral research, 24*(4), 433–437. https://doi.org/10.1590/s1806-83242010000400010 | CVM stage not based on Baccetti’s method (2005) |
| 2010 | Rasool, G., Bashir, U., & Kundi, I. U. (2010). Comparative evaluation between cervical vertebrae and hand-wrist maturation for assessment of skeletal maturity orthodontic patients. *Pakistan Oral & Dental Journal, 30*(1), 85-95. | CVM stage not based on Baccetti’s method (2005) |
| 2010 | Santos, M. F., Lima, R. L., Ary-Pires, B., Pires-Neto, M. A., & Ary-Pires, R. (2010). Developmental steps of the human cervical spine: parameters for evaluation of skeletal maturation stages. *Anatomical science international, 85*(2), 105–114. https://doi.org/10.1007/s12565-009-0065-7 | CVM stage not based on Baccetti’s method (2005) |
| 2010 | Generoso, R., Sadoco, E. C., Armond, M. C., & Gameiro, G. H. (2010). Evaluation of mandibular length in subjects with Class I and Class II skeletal patterns using the cervical vertebrae maturation*. Brazilian oral research, 24*(1), 46–51. https://doi.org/10.1590/s1806-83242010000100008 | CVM stage not based on Baccetti’s method (2005) |
| 2010 | Rasool, G., Bashir, U., & Kundi, I. I. (2010). Comparative evaluation between cervical vertebrae and hand-wrist maturation for assessment of skeletal maturity orthodontic patients. *Pakistan Oral & Dental Journal,* 30(1), 85-95*.* | No mean or/and standard deviation or/and number of subjects values |
| 2010 | Joshi, V. V., Iyengar, A. R., Nagesh, K. S. & Gupta, J. (2010). Comparative study between cervical vertebrae and hand-wrist maturation for the assessment of skeletal age. *Revista de Clínica e Pesquisa Odontológica, 6*(3), 207-213. | CVM stage not based on Baccetti’s method (2005) |
| 2010 | Alhadlaq, A. M. (2010). Prediction of Mandibular Growth Potential Using Cervical Vertebral Bone Age in Saudi Subjects. *Journal of King Saud University, 22*(1), 1-7. | CVM stage not based on Baccetti’s method (2005) |
| 2010 | Warmeling, D., Rodrigues, K. M., Zastrow, M. D., & Thiesen, G. (2010). Comparative study of two skeletal maturation evaluation indexes. *Revista Odonto Ciência, 25(2)*, 188-193. | CVM stage not based on Baccetti’s method (2005) |
| 2010 | Litsas, G., & Ari-Demirkaya, A. (2010). Growth indicators in orthodontic patients. Part 2: comparison of cervical bone age to hand-wrist skeletal age. Relationship with chronological age. *European journal of paediatric dentistry, 11*(4), 176–180. | CVM stage not based on Baccetti’s method (2005) |
| 2010 | Celli, D., Gasperoni, E., Oliva, B., & Deli, R. (2010). Assessment of mandibular growth and response to functional appliance treatment in prepubertal patients with different auxologic categories. *Progress in orthodontics, 11*(1), 20–26. https://doi.org/10.1016/j.pio.2010.04.010 | CVM stage not based on Baccetti’s method (2005) |
| 2010 | Di Vece, L., Faleri, G., Picciotti, M., Guido, L., & Giorgetti, R. (2010). Does a transverse maxillary deficit affect the cervical vertebrae? A pilot study. *American journal of orthodontics and dentofacial orthopedics, 137*(4), 515–519. https://doi.org/10.1016/j.ajodo.2009.12.001 | CVM stage not based on Baccetti’s method (2005) |
| 2010 | Baccetti T. (2010). Malocclusions de classe II: bien choisir le moment du traitement pour optimiser l'effet orthopédique des appareils fonctionnels. *L' Orthodontie francaise, 81*(4), 279–286. https://doi.org/10.1051/orthodfr/2010026 | No mean or/and standard deviation or/and number of subjects values |
| 2010 | Guest, S. S., McNamara, J. A., Baccetti, T., & Franchi, L. (2010). Improving Class II malocclusion as a side-effect of rapid maxillary expansion: a prospective clinical study. *American journal of orthodontics and dentofacial orthopedics, 138*(5), 582–591. https://doi.org/10.1016/j.ajodo.2008.12.026 | No mean or/and standard deviation or/and number of subjects values |
| 2010 | Singh, S., Singh, M., Saini, A., Misra, V., Sharma, V., & Singh, G. (2010). Timing of Myofunctional Appliance Therapy. *Journal of Clinical Pediatric Dentistry, 35*(2), 233–240. doi:10.17796/jcpd.35.2.9572h13218806871 | No mean or/and standard deviation or/and number of subjects values |
| 2010 | Thiesen, G., Silva, M. F. B., & Zastrow, M. D. (2010). Estudo comparativo entre dois índices de estimativa da maturação esquelética em crianças HlV positivo: índice carpal e índice vertebral. *Revista Ortodontia Gaúcha, 14*(1), 11-20. | No mean or/and standard deviation or/and number of subjects values |
| 2010 | Mayarí, G. T., & Lugo, R. O. (2010). Evaluación de la maduración ósea a través de las vértebras cervicales en pacientes de ortodoncia. *Revista Cubana de Estomatología, 47*(3), 326-335. | No mean or/and standard deviation or/and number of subjects values |
| 2010 | Fudalej, P., & Bollen, A. M. (2010). Effectiveness of the cervical vertebral maturation method to predict postpeak circumpubertal growth of craniofacial structures. *American journal of orthodontics and dentofacial orthopedics, 137*(1), 59–65. https://doi.org/10.1016/j.ajodo.2008.01.018 | CVM stage not based on Baccetti’s method (2005) |
| 2010 | Sukhia, R. H., & Fida, M. (2010). Correlation among chronologic age, skeletal maturity, and dental age. *World Journal of Orthodontics, 11*(4). | CVM stage not based on Baccetti’s method (2005) |
| 2010 | Toledo Mayarí, G., & Otaño Lugo, R. (2010). Assessment of bone maturation in cervical vertebrae in Orthodontics patients. *Revista Cubana de Estomatologia, 47*(3), 326-335. | CVM stage not based on Baccetti’s method (2005) |
| 2011 | Sachan, K., Sharma, V. P., & Tandon, P. (2011). A correlative study of dental age and skeletal maturation. Indian journal of dental research, 22(6), 882. https://doi.org/10.4103/0970-9290.94698 | CVM stage not based on Baccetti’s method (2005) |
| 2011 | Machorowska-Pieniążek, A. (2011). Morphometric Assessment of the Bone Age, Sequence of Developmental Changes of the Cervical Vertebrae . Dental and Medical problems, 48(3), 335-341. | CVM stage not based on Baccetti’s method (2005) |
| 2011 | Gupta, K. P., Garg, S., & Grewal, P. S. (2011). Establishing a diagnostic tool for assessing optimal treatment timing in Indian children with developing malocclusions. *Journal of Clinical and Experimental Dentistry, 3*(1), 1–4. https://doi.org/10.4317/jced.3.e18 | CVM stage not based on Baccetti’s method (2005) |
| 2011 | Ball, G., Woodside, D., Tompson, B., Hunter, W. S., & Posluns, J. (2011). Relationship between cervical vertebral maturation and mandibular growth. *American journal of orthodontics and dentofacial orthopedics, 139*(5), 455–461. | No mean or/and standard deviation or/and number of subjects values |
| 2011 | Costacurta, M., Condò, R., Sicuro, L., Perugia, C., & Docimo, R. (2011). Cervical vertebral maturation and dental age in coeliac patients. *ORAL & implantology, 4*(3), 11–17. | CVM stage not based on Baccetti’s method (2005) |
| 2011 | Nestman, T. S., Marshall, S. D., Qian, F., Holton, N., Franciscus, R. G., & Southard, T. E. (2011). Cervical vertebrae maturation method morphologic criteria: poor reproducibility. *American journal of orthodontics and dentofacial orthopedics, 140*(2), 182–188. https://doi.org/10.1016/j.ajodo.2011.04.013 | No mean or/and standard deviation or/and number of subjects values |
| 2011 | Al-Taqi, A., & Alawadhi, S. (2011). Comparative evaluation of cervical vertebral maturation assessment with hand and wrist analysis in skeletal maturity. *Pakistan Orthodontic Journal, 3*(1), 3-10. | CVM stage not based on Baccetti’s method (2005) |
| 2011 | Mahajan S. (2011). Evaluation of skeletal maturation by comparing the hand wrist radiograph and cervical vertebrae as seen in lateral cephalogram. *Indian journal of dental research, 22*(2), 309–316. https://doi.org/10.4103/0970-9290.84310 | CVM stage not based on Baccetti’s method (2005) |
| 2011 | Murthy, V., Begum, A., Kumar, P., & Lalitha, C. H. (2011).Reliability of an Objective Method of Evaluating Skeletal Maturity based on Cervical Vertebral Bone Age as Compared with the TW2 Method. *The Journal of Indian Orthodontic Society, 47*(4), 202-206. | CVM stage not based on Baccetti’s method (2005) |
| 2011 | Hu, H., Liu, H., Chen, L., Hung, C., Xu, X., & Lan, Z. (2011). Image Segmentation of Cervical Vertebra in X-Ray Radiographs Using the Curve Fitting Strategy. *Proceedings of the 2011 ACM Symposium on Applied Computing,* 853-858. | CVM stage not based on Baccetti’s method (2005) |
| 2011 | Baccetti, T., Franchi, L., & McNamara, J. A., Jr (2011). Longitudinal growth changes in subjects with deepbite. *American journal of orthodontics and dentofacial orthopedics, 140*(2), 202–209. https://doi.org/10.1016/j.ajodo.2011.04.015 | No mean or/and standard deviation or/and number of subjects values |
| 2011 | Franchi, L., Alvetro, L., Giuntini, V., Masucci, C., Defraia, E., & Baccetti, T. (2011). Effectiveness of comprehensive fixed appliance treatment used with the Forsus Fatigue Resistant Device in Class II patients. *The Angle orthodontist, 81*(4), 678–683. https://doi.org/10.2319/102710-629.1 | No mean or/and standard deviation or/and number of subjects values |
| 2011 | Martins, M. M., Oliveira, P. C. B., Andrade Goldner, M. T., & Miguel, J. A. M. (2011). Skeletal maturation of cervical vertebrae and hand-wrist region. *Brazilian Dental Science, 14*(1), 4-8. | CVM stage not based on Baccetti’s method (2005) |
| 2011 | Costacurta, M., Condò, R., Sicuro, L., Perugia, C., & Docimo, R. (2011). Cervical vertebral maturation and dental age in coeliac patients. *ORAL & implantology, 4*(3-4), 11–17. | No mean or/and standard deviation or/and number of subjects values |
| 2011 | Baptista, R. S., Hummel, A. D., Finkelsztain, R. A., Ortolani, C. L. F., & Pisa, I. T. (2011). Desenvolvimento e avaliação de um classificador de padrões para análise do crescimento facial pelo método de maturação vertebral cervical. *Journal of Health Informatics, 3*(2), 64-68. | No mean or/and standard deviation or/and number of subjects values |
| 2011 | Ghislanzoni, L. T., Toll, D. E., Defraia, E., Baccetti, T., & Franchi, L. (2011). Treatment and posttreatment outcomes induced by the Mandibular Advancement Repositioning Appliance; a controlled clinical study. *The Angle orthodontist, 81*(4), 684–691. https://doi.org/10.2319/111010-656.1 | No mean or/and standard deviation or/and number of subjects values |
| 2011 | Yoganandan, N., Pintar, F. A., Lew, S. M., Rao, R. D., & Rangarajan, N. (2011). Quantitative analyses of pediatric cervical spine ossification patterns using computed tomography. *Annals of advances in automotive medicine, 55*, 159–168. | No mean or/and standard deviation or/and number of subjects values |
| 2011 | Sachan, K., Tandon, P., & Sharma, V. (2011). A correlative study of dental age and skeletal maturation. Indian Journal of Dental Research, 22(6), 882. https://doi.org/10.4103/0970-9290.94698 | No mean or/and standard deviation or/and number of subjects values |
| 2011 | Sigler, L. M., Baccetti, T., & McNamara, J. A., Jr (2011). Effect of rapid maxillary expansion and transpalatal arch treatment associated with deciduous canine extraction on the eruption of palatally displaced canines: A 2-center prospective study. *American journal of orthodontics and dentofacial orthopedics*, 139(3), 235–244. https://doi.org/10.1016/j.ajodo.2009.07.015 | No mean or/and standard deviation or/and number of subjects values |
| 2012 | Gottimukkala, P., Gandikota, C. S., Perumalla, P. L., Palla, Y., & Juvvadi, S. (2012). Assessment of Skeletal and Dental Maturation of Short and Long-Face Children of South Indian Population. The Journal of Indian Orthodontic Society, 46, 148–153. https://doi.org/10.5005/jp-journals-10021-1078 | CVM stage not based on Baccetti’s method (2005) |
| 2012 | Valizadeh, S., Eil, N., Ehsani, S., & Bakhshandeh, H. (2012). Correlation between dental and cervical vertebral maturation in Iranian females. *Iranian journal of radiology, 10*(1), 1–7. https://doi.org/10.5812/iranjradiol.9993 | CVM stage not based on Baccetti’s method (2005) |
| 2012 | Moraes, M. B., Moraes, M. E. L., Raldi, F. V., Ragone, S. M. G., & Holleben, D. (2012). Evaluation and development of a bone age assessment method in patients with Down syndrome. *Brazilian Dental Science, 15*(2), 6–11. https://doi.org/10.14295/bds.2012.v15i2.760 | CVM stage not based on Baccetti’s method (2005) |
| 2012 | Bhatia, A. S., Shah, R. B., Singh, A., & Paul, R. (2012). Evaluating Period of Accelerated Skeletal Maturation in Gujarati Children between Ages 8+ and 14+ Years. *Journal of Indian Orthodontic Society, 46*(4), 250–253. https://doi.org/10.5005/jp-journals-10021-1099 | CVM stage not based on Baccetti’s method (2005) |
| 2012 | Varshosaz, M., Ehsani, S., Nouri, M., & Tavakoli, M. A. (2012). Bone age estimation by cervical vertebral dimensions in lateral cephalometry. Progress in Orthodontics, 13(2), 126–131. https://doi.org/10.1016/j.pio.2011.09.003 | CVM stage not based on Baccetti’s method (2005) |
| 2012 | EmamiMeibodi, S., Pousti, M., Fetrati, A., KharraziFard, M. (2012). Assessment of the Relation between Clinical Phases of Dental Eruption and Skeletal Maturity Stages Using Cervical Vertebrae Method. *Journal of Mashhad Dental School*, 36(2) 95-104. | No mean or/and standard deviation or/and number of subjects values |
| 2012 | Kothavade, D. S., Pandey, R. K., & Nagar, A. (2012). An assessment of the relationship between cervical vertebrae maturation index and eruption of permanent maxillary canines. *Journal of the Indian Society of Pedodontics and Preventive Dentistry, 30*(4), 301–304. https://doi.org/10.4103/0970-4388.108925 | No mean or/and standard deviation or/and number of subjects values |
| 2012 | Costacurta, M., Sicuro, L., Di Renzo, L., Condò, R., De Lorenzo, A., & Docimo, R. (2012). Childhood obesity and skeletal-dental maturity. European journal of paediatric dentistry, 13(2), 128–132. | CVM stage not based on Baccetti’s method (2005) |
| 2012 | Hegde, D. Y., Baliga, S., Yeluri, R., & Munshi, A. K. (2012). Digital radiograph of the middle phalanx of the third finger (MP3) region as a tool for skeletal maturity assessment. Indian journal of dental research, 23(4), 447–453. https://doi.org/10.4103/0970-9290.104947 | CVM stage not based on Baccetti’s method (2005) |
| 2012 | Armond, M. C., Generoso, R., Falci, S. G. M., Ramos-Jorge, M. L., & Marques, L. S. (2012). Skeletal maturation of the cervical vertebrae: Association with various types of malocclusion. Brazilian Oral Research, 26(2), 145–150. https://doi.org/10.1590/S1806-83242012005000003 | CVM stage not based on Baccetti’s method (2005) |
| 2012 | Perinetti, G., Franchi, L., Castaldo, A., & Contardo, L. (2012). Gingival crevicular fluid protein content and alkaline phosphatase activity in relation to pubertal growth phase. The Angle orthodontist, 82(6), 1047–1052. https://doi.org/10.2319/123111-806.1 | No mean or/and standard deviation or/and number of subjects values |
| 2012 | Nogueira, F. E. P. T., Ayub, O. S., Freitas, P. Z., Gomide, R. T., & Lara, T. S. (2012). Avaliação da morfologia vertebral em pacientes adultos: Um indicador maturacional. Revista brasileira de odontologia, 69(1), 116–119. | No mean or/and standard deviation or/and number of subjects values |
| 2012 | Thevissen, P. W., Kaur, J., & Willems, G. (2012). Human age estimation combining third molar and skeletal development. International journal of legal medicine, 126(2), 285–292. https://doi.org/10.1007/s00414-011-0639-5. | No mean or/and standard deviation or/and number of subjects values |
| 2012 | Shim, J. J., Heo, G., Lagravère, M. O. (2012). Évaluation de la maturation squelettique à l’aide des vertèbres cervicales et par CBCT. International Orthodontics, 10(4), 351–362. | No mean or/and standard deviation or/and number of subjects values |
| 2012 | Zhao, X. G., Lin, J., Jiang, J. H., Wang, Q., & Ng, S. H. (2012). Validity and reliability of a method for assessment of cervical vertebral maturation. *The Angle orthodontist, 82*(2), 229–234. https://doi.org/10.2319/051511-333.1 | No mean or/and standard deviation or/and number of subjects values |
| 2012 | Baptista, R. S., Quaglio, C. L., Mourad, L. M., Hummel, A. D., Caetano, C. A., Ortolani, C. L., & Pisa, I. T. (2012). A semi-automated method for bone age assessment using cervical vertebral maturation. *The Angle orthodontist, 82*(4), 658–662. https://doi.org/10.2319/070111-425.1 | No mean or/and standard deviation or/and number of subjects values |
| 2012 | Giuca, M. R., Pasini, M., Tecco, S., Marchetti, E., Giannotti, L., & Marzo, G. (2012). Skeletal maturation in obese patients. *American journal of orthodontics and dentofacial orthopedics, 142*(6), 774–779. https://doi.org/10.1016/j.ajodo.2012.07.011 | No mean or/and standard deviation or/and number of subjects values |
| 2012 | Ishaq, R. A., Soliman, S. A., Foda, M. Y., & Fayed, M. M. (2012). Insulin-like growth factor I: a biologic maturation indicator. *American journal of orthodontics and dentofacial orthopedics, 14*2(5), 654–661. https://doi.org/10.1016/j.ajodo.2012.06.015 | CVM stage not based on Baccetti’s method (2005) |
| 2012 | Joshi, V., Yamaguchi, T., Matsuda, Y., Kaneko, N., Maki, K., & Okano, T. (2012). Skeletal maturity assessment with the use of cone-beam computerized tomography. *Oral surgery, oral medicine, oral pathology and oral radiology, 113*(6), 841–849. https://doi.org/10.1016/j.oooo.2011.11.018 | CVM stage not based on Baccetti’s method (2005) |
| 2012 | Perinetti, G., Contardo, L., Gabrieli, P., Baccetti, T., & Di Lenarda, R. (2012). Diagnostic performance of dental maturity for identification of skeletal maturation phase. *European journal of orthodontics, 34*(4), 487–492. https://doi.org/10.1093/ejo/cjr027 | No mean or/and standard deviation or/and number of subjects values |
| 2012 | Yang, C., Mi, C. B., & Zu Qing, X. H. (2012). Correlation of Han adolescents’s hand-wrist bone maturation and cervical vertebrae maturation in Urumqi region. *Chinese Journal of Tissue Engineering Research, 16*(35), 6641-6645. | No mean or/and standard deviation or/and number of subjects values |
| 2012 | Gupta, S., Jain, S., Gupta, P., & Deoskar, A. (2012). Determining skeletal maturation using insulin-like growth factor I (IGF-I) test. *Progress in orthodontics, 13*(3), 288–295. https://doi.org/10.1016/j.pio.2011.09.006 | No mean or/and standard deviation or/and number of subjects values |
| 2012 | Vahdettin, L., & Altuğ, Z. (2012). Longitudinal soft-tissue profile changes in adolescent Class I subjects. *Journal of Orofacial Orthopedics, 73*(6), 440–453. doi:10.1007/s00056-012-0099-7 | No mean or/and standard deviation or/and number of subjects values |
| 2012 | Dolphens, M., Cagnie, B., Coorevits, P., Vanderstraeten, G., Cardon, G., Dʼhooge, R., & Danneels, L. (2012). Sagittal standing posture and its association with spinal pain: a school-based epidemiological study of 1196 Flemish adolescents before age at peak height velocity. Spine, 37(19), 1657–1666. https://doi.org/10.1097/BRS.0b013e3182408053 | No mean or/and standard deviation or/and number of subjects values |
| 2012 | Bebnowski, D., Hänggi, M. P., Markic, G., Roos, M., & Peltomäki, T. (2012). Cervical vertebrae anomalies in subjects with Class II malocclusion assessed by lateral cephalogram and cone beam computed tomography. *European journal of orthodontics, 34*(2), 226–231. https://doi.org/10.1093/ejo/cjq192 | No mean or/and standard deviation or/and number of subjects values |
| 2012 | Masoud, M. I., Marghalani, H. Y., Masoud, I. M., & Gowharji, N. F. (2012). Prospective longitudinal evaluation of the relationship between changes in mandibular length and blood-spot IGF-1 measurements. American journal of orthodontics and dentofacial orthopedics, 141(6), 694–704. https://doi.org/10.1016/j.ajodo.2011.12.021 | No mean or/and standard deviation or/and number of subjects values |
| 2012 | Karwacki, G. M., & Schneider, J. F. (2012). Normal ossification patterns of atlas and axis: a CT study. AJNR. American journal of neuroradiology, 33(10), 1882–1887. https://doi.org/10.3174/ajnr.A3105 | No mean or/and standard deviation or/and number of subjects values |
| 2012 | Raberin, M., Cozor, I., & Gobert-Jacquart, S. (2012). Les vertèbres cervicales : indicateurs du dynamisme de la croissance mandibulaire ?. L' Orthodontie francaise, 83(1), 45–58. https://doi.org/10.1051/orthodfr/2011148 | No mean or/and standard deviation or/and number of subjects values |
| 2012 | Chen, L., Lan, Z., Xu, X., Lin, J., & Hu, H. (2012). Accuracy and repeatability of computer aided cervical vertebra landmarking in cephalogram. *Journal of Huazhong University of Science and Technology Medical sciences , 32*(1), 119–123. https://doi.org/10.1007/s11596-012-0021-y | CVM stage not based on Baccetti’s method (2005) |
| 2012 | Al-Jewair, T. S., Preston, C. B., Moll, E. M., & Dischinger, T. (2012). A comparison of the MARA and the AdvanSync functional appliances in the treatment of Class II malocclusion. *The Angle orthodontist, 82*(5), 907–914. https://doi.org/10.2319/090411-569.1 | No mean or/and standard deviation or/and number of subjects values |
| 2012 | Lucchese, A., Carinci, F., & Brunelli, G. (2012). Skeletal effects induced by twin block in therapy of class II malocclusion. *European Journal of Inflammation, 10*(1), 83-86. | No mean or/and standard deviation or/and number of subjects values |
| 2012 | Altan, M., Nebioğlu Dalci, Ö., & İseri, H. (2012). Growth of the cervical vertebrae in girls from 8 to 17 years. A longitudinal study. *European journal of orthodontics, 34*(3), 327–334. https://doi.org/10.1093/ejo/cjr013 | CVM stage not based on Baccetti’s method (2005) |
| 2012 | Timmins, K., Liversidge, H., Farella, M., Herbison, P., & Kieser, J. (2012). The usefulness of dental and cervical maturation stages in New Zealand children for Disaster Victim Identification. *Forensic science, medicine, and pathology, 8*(2), 101–108. https://doi.org/10.1007/s12024-011-9251-8 | CVM stage not based on Baccetti’s method (2005) |
| 2012 | Kumar, S., Singla, A., Sharma, R., Virdi, M. S., Anupam, A., & Mittal, B. (2012). Skeletal maturation evaluation using mandibular second molar calcification stages. *The Angle orthodontist, 82*(3), 501–506. https://doi.org/10.2319/051611-334.1 | CVM stage not based on Baccetti’s method (2005) |
| 2013 | Tiro, Alisa;Nakas, E;Dzemidzic, V; Redzepagic-Vrazalica, L. (2013). Growth indicators in Orthodontic Patients: Cervical Bone age vs chronological age*. Stomatoloski Vjesnik 2*(1), 3-7. | No mean or/and standard deviation or/and number of subjects values |
| 2013 | Sun, L., & Li, W. R. (2013). Cervical vertebral maturation of female children with orofacial clefts. The Cleft palate-craniofacial journal, 50(5), 535–541. https://doi.org/10.1597/11-215 | No mean or/and standard deviation or/and number of subjects values |
| 2013 | Ballrick, J. W., Fields, H., Beck, F., Sun, Z., & Germak, J. (2013). The cervical vertebrae staging method's reliability in detecting pre and post mandibular growth. *Orthodontic Waves*, 72(3), 105-111. | CVM stage not based on Baccetti’s method (2005) |
| 2013 | Prasad, C. K., Reddy, V. N., Sreedevi, G., Ponnada, S. R., Priya, K. P., & Naik, B. R. (2013). Objective evaluation of cervical vertebral bone age' its reliability in comparison with hand-wrist bone age: by TW3 method. The journal of contemporary dental practice, 14(5), 806–813. https://doi.org/10.5005/jp-journals-10024-1407 | CVM stage not based on Baccetti’s method (2005) |
| 2013 | Mellion, Z. J., Behrents, R. G., & Johnston, L. E., Jr. (2013). The pattern of facial skeletal growth and its relationship to various common indexes of maturation. American journal of orthodontics and dentofacial orthopedics, 143(6), 845–854. https://doi.org/10.1016/j.ajodo.2013.01.019 | No mean or/and standard deviation or/and number of subjects values |
| 2013 | Alhadlaq, A. M., & Al-Maflehi, N. S. (2013). New model for cervical vertebral bone age estimation in boys. King Saud University Journal of Dental Sciences, 4(1), 1–5. https://doi.org/10.1016/j.ksujds.2012.11.001 | CVM stage not based on Baccetti’s method (2005) |
| 2013 | Krisztina, M. I., A, O., Réka, G., & Zsuzsa, B. (2013). Evaluation of the Skeletal Maturation Using Lower First Premolar Mineralisation. Acta Medica Marisiensis, 59(6), 289–292. https://doi.org/10.2478/amma-2013-0066 | CVM stage not based on Baccetti’s method (2005) |
| 2013 | MacK, K. B., Phillips, C., Jain, N., & Koroluk, L. D. (2013). Relationship between body mass index percentile and skeletal maturation and dental development in orthodontic patients. American Journal of Orthodontics and Dentofacial Orthopedics, 143(2), 228–234. https://doi.org/10.1016/j.ajodo.2012.09.015 | No mean or/and standard deviation or/and number of subjects values |
| 2013 | Navlani, M., & Makhija, P.G. (2013). Evaluation of skeletal and dental maturity indicators and assessment of cervical vertebral maturation stages by height/width ratio of third cervical vertebra. Journal of Pierre Fauchard Academy, 27(3), 73–80. | CVM stage not based on Baccetti’s method (2005) |
| 2013 | Perinetti, G., Callovi, M., Salgarello, S., Biasotto, M., Contardo, L. (2013). Eruption of the permanent maxillary canines in relation to mandibular second molar maturity. The Angle Orthodontist, 83(4), 578–583. | No mean or/and standard deviation or/and number of subjects values |
| 2013 | Alhadlaq, A. M., & Al-Shayea, E. I. (2013). New method for evaluation of cervical vertebral maturation based on angular measurements. *Saudi medical journal, 34*(4), 388–394. | CVM stage not based on Baccetti’s method (2005) |
| 2013 | Tikku, T., Khanna, R., Sachan, K., & Agrawal, S. (2013). Correlation of Improved Version of Cervical Vertebral Maturation Indicator with Other Growth Maturity Indicators. *The Journal of Indian Orthodontic Society, 47*(1),28-32. | No mean or/and standard deviation or/and number of subjects values |
| 2013 | Alhadlaq, A. M., & Al-Maflehi, N. S. (2013). New model for cervical vertebral bone age estimationin boys. *King Saud University Journal of Dental Sciences, 4*(1), 1-5. | CVM stage not based on Baccetti’s method (2005) |
| 2013 | Aguiar, L. B., Caldas, M., Haiter Neto, F., & Ambrosano, G. M. (2013). A methodology to measure cervical vertebral bone maturation in a sample from low-income children. *Brazilian dental journal, 24*(1), 30–34. https://doi.org/10.1590/0103-6440201301787 | CVM stage not based on Baccetti’s method (2005) |
| 2013 | Casey, C., Gill, D. S., & Jones, S. P. (2013). A comparison of skeletal maturation in patients with tooth agenesis and unaffected controls assessed by the cervical vertebral maturation (CVM) index. *Journal of orthodontics, 40*(4), 286–298. https://doi.org/10.1179/1465313313Y.0000000070 | No mean or/and standard deviation or/and number of subjects values |
| 2013 | Moshfeghi, M., Rahimi, H., Rahimi, H., Nouri, M., & Bagheban, A. A. (2013). Predicting mandibular growth increment on the basis of cervical vertebral dimensions in Iranian girls. *Progress in orthodontics, 14*(1), 1-6. https://doi.org/10.1186/2196-1042-14-3 | CVM stage not based on Baccetti’s method (2005) |
| 2013 | Chalasani, S., Kumar, J., Prasad, M., Shetty, B. S. K., & Kumar, T. A. (2013). An Evaluation of Skeletal Maturation by Hand-Wrist Bone Analysis and Cervical Vertebral Analysis: A Comparitive Study. *The journal of Indian Orthodontic Society, 47*(4), 433-437. | CVM stage not based on Baccetti’s method (2005) |
| 2013 | Torres, F. C., Yamazaki, M. S., Jóias, R. P., Paranhos, L. R., Rode, S., Siqueira, D. F., & Fuziy, A. (2013). Evaluation of the cervical vertebrae maturation index in lateral cephalograms taken in different head positions. Brazilian dental journal, 24(5), 462–466. https://doi.org/10.1590/0103-6440201302233 | No mean or/and standard deviation or/and number of subjects values |
| 2013 | Bedoya, A., Gallego, M., Pamplona, S., Soto, N., Bermudez, P., & Tamayo, J. (2013). Maduración ósea vertebral en niños de 8 -14 años de la clínica del postgrado de ortodoncia de la Institución Universitaria Colegios de Colombia (UNICOC) sede Santiago de Cali. *Revista estomatologia y Salud, 21*(2), 14-19. | No mean or/and standard deviation or/and number of subjects values |
| 2013 | Prasad, M., Ganji, V. S., George, S. A., Talapaneni, A. K., & Shetty, S. K. (2013). A comparison between cervical vertebrae and modified MP3 stages for the assessment of skeletal maturity. *Journal of natural science, biology, and medicine, 4*(1), 74–80. https://doi.org/10.4103/0976-9668.107264 | CVM stage not based on Baccetti’s method (2005) |
| 2013 | Pasciuti, E., Franchi, L., Baccetti, T., Milani, S., & Farronato, G. (2013). Comparison of three methods to assess individual skeletal maturity. *Journal of orofacial orthopedics, 74*(5), 397–408. https://doi.org/10.1007/s00056-013-0164-x | No mean or/and standard deviation or/and number of subjects values |
| 2013 | Shim, J., Heo, G., & Lagravère, M. O. (2013). Correlation between three-dimensional morphological changes of the hyoid bone with other skeletal maturation methods in adolescents. *Oral surgery, oral medicine, oral pathology and oral radiology, 116*(4), 511–517. https://doi.org/10.1016/j.oooo.2013.06.025 | No mean or/and standard deviation or/and number of subjects values |
| 2013 | Durka-Zając, M., Marcinkowska, A., & Mituś-Kenig, M. (2013). Bone age assessment using cephalometric photographs. *Polish journal of radiology*, *78*(2), 19–25. https://doi.org/10.12659/PJR.889072 | No mean or/and standard deviation or/and number of subjects values |
| 2013 | Gupta, G., Mogra, S., Shetty, V. S., Shetty, S., Goyal, S., & Garg, S. (2013). Hand Wrist Radiographs-Are They Really Required As Maturation Indicators????. *Indian Journal of Dental Sciences, 5*(1), 30-33. | CVM stage not based on Baccetti’s method (2005) |
| 2013 | Jain, S., Jain, S., Deoskar, A., & Prasad, V. S. (2013). Serum IGF-1 levels as a clinical tool for optimizing orthodontic treatment timing. *Progress in orthodontics, 14*, 1-7. https://doi.org/10.1186/2196-1042-14-46 | No mean or/and standard deviation or/and number of subjects values |
| 2013 | Ellyeus, M. K., Sjahruddin, L., Sudhana, W., & Koesoemahardja, H. D. (2013). Correlation Craniofacial Growth, Body Height And Cervical Vertebrae Maturation Stages. *Journal of Dentistry Indonesia, 18*(3), 73-76. | CVM stage not based on Baccetti’s method (2005) |
| 2013 | Chhibber, A., Upadhyay, M., Uribe, F., & Nanda, R. (2013). Mechanism of Class II correction in prepubertal and postpubertal patients with Twin Force Bite Corrector. *The Angle orthodontist, 83*(4), 718–727. https://doi.org/10.2319/090412-709.1 | No mean or/and standard deviation or/and number of subjects values |
| 2013 | Szeląg, E., Paradowska-Stolarz, A., Noga, L., Pietruszka, K., Szumko, M., & Ogiński, T. (2013). Does the Baccetti’s Method of Establishing of Skeletal Age Have Clinical Impotance? *Dental and Medical Problems, 50*(4), 449-453. | CVM stage not based on Baccetti’s method (2005) |
| 2013 | Tiro, A., Nakas, E., Dzemidzic, V., & Redzepagic-Vrazalica, L. (2013).Growth indicators in orthodontic patients: cervical bone age vs chronological age. *Stomatoloski vjesnik, 2*(1), 3-7. | CVM stage not based on Baccetti’s method (2005) |
| 2013 | Alali, O., Sawan, M. N., & Kaddah, A. (2013). Correlation of Two Radiographic Methods of Skeletal Maturation Stages Determination in Class II Skeletal Malocclusion Patients at Puberty*. Damascus University Journal for Health and Sciences, 29*(1), 511-520. | CVM stage not based on Baccetti’s method (2005) |
| 2013 | Paluch, Z., Wojtyna, J., & Misiołek, M. (2013). The influence of nasopharyngeal patency on the morphology of nasomaxillary complex. Acta odontologica Scandinavica, 71(6), 1599–1605. https://doi.org/10.3109/00016357.2013.780291 | No mean or/and standard deviation or/and number of subjects values |
| 2013 | Yoganandan, N., Pintar, F. A., Lew, S. M., & Rao, R. D. (2013). Geometrical Properties of the Human Child Cervical Spine With a Focus on the C1 Vertebra. *Traffic Injury Prevention, 15*(3), 287–293. doi:10.1080/15389588.2013.811719 | CVM stage not based on Baccetti’s method (2005) |
| 2013 | Rao, R. D., Tang, S., Lim, C., & Yoganandan, N. (2013). Developmental Morphology and Ossification Patterns of the C1 Vertebra. The Journal of Bone and Joint Surgery-American Volume, 95(17), 1241–1247. https://doi.org/10.2106/JBJS.L.01035. | CVM stage not based on Baccetti’s method (2005) |
| 2013 | Albert, M. A., & Maier, C. A. (2013). Epiphyseal Union of the Cervical Vertebral Centra: Its Relationship to Skeletal Age and Maturation of Thoracic Vertebral Centra. *Journal of Forensic Sciences, 58*(6), 1568–1574. doi:10.1111/1556-4029.12232 | CVM stage not based on Baccetti’s method (2005) |
| 2013 | Franchi, L., Pavoni, C., Faltin, K., Jr, McNamara, J. A., & Cozza, P. (2013). Long-term skeletal and dental effects and treatment timing for functional appliances in Class II malocclusion. *The Angle orthodontist, 83*(2), 334–340. https://doi.org/10.2319/052912-450.1 | No mean or/and standard deviation or/and number of subjects values |
| 2013 | Perinetti, G., Callovi, M., Salgarello, S., Biasotto, M., & Contardo, L. (2013). Eruption of the permanent maxillary canines in relation to mandibular second molar maturity. T*he Angle orthodontist, 83*(4), 578–583. https://doi.org/10.2319/090212-704.1 | No mean or/and standard deviation or/and number of subjects values |
| 2013 | Landázuri, D. R., Raveli, D. B., Santos-Pinto, A., Dib, L. P., & Maia, S. (2013). Changes on facial profile in the mixed dentition, from natural growth and induced by Balters' bionator appliance. *Dental press journal of orthodontics, 18*(2), 108–115. https://doi.org/10.1590/s2176-94512013000200022 | No mean or/and standard deviation or/and number of subjects values |
| 2013 | Juloski, J., Glisic, B., Scepan, I., Milasin, J., Mitrovic, K., & Babic, M. (2013). Ontogenetic changes of craniofacial complex in Turner syndrome patients treated with growth hormone. *Clinical oral investigations, 17*(6), 1563–1571. https://doi.org/10.1007/s00784-012-0844-8 | No mean or/and standard deviation or/and number of subjects values |
| 2013 | Franchi, L., Pavoni, C., Faltin, K., Jr, McNamara, J. A., Jr, & Cozza, P. (2013). Long-term skeletal and dental effects and treatment timing for functional appliances in Class II malocclusion. The Angle orthodontist, 83(2), 334–340.https://doi.org/10.2319/052912-450.11 | No mean or/and standard deviation or/and number of subjects values |
| 2013 | Patcas, R., Signorelli, L., Peltomäki, T., & Schätzle, M. (2013). Is the use of the cervical vertebrae maturation method justified to determine skeletal age? A comparison of radiation dose of two strategies for skeletal age estimation. *European journal of orthodontics, 35*(5), 604–609. https://doi.org/10.1093/ejo/cjs043 | No mean or/and standard deviation or/and number of subjects values |
| 2013 | Furquim, B. D., Henriques, J. F., Janson, G., Siqueira, D. F., & Furquim, L. Z. (2013). Effects of mandibular protraction appliance associated to fixed appliance in adults. *Dental press journal of orthodontics, 18*(5), 46–52. https://doi.org/10.1590/s2176-94512013000500009 | CVM stage not based on Baccetti’s method (2005) |
| 2013 | Ravi, M., & Ravikala, S. (2013). Assessment of skeletal age in children with unilateral cleft lip and palate. *International journal of clinical pediatric dentistry, 6*(3), 151–155. https://doi.org/10.5005/jp-journals-10005-1209 | No mean or/and standard deviation or/and number of subjects values |
| 2013 | Ramos, N. A. A., Lozano, M. B., & Ocampo, A. M. (2013). Análisis comparativo entre la edad ósea, edad dental y edad cronológica. *Revista Mexicana de Ortodoncia*, 1(1), 33-37. | No mean or/and standard deviation or/and number of subjects values |
| 2013 | Pisek, P., Godfrey, K., Manosudprasit, M., Wangsrimongkol, T., & Leelasinjaroen, P. (2013). A comparison of cervical vertebral maturation assessment of skeletal growth stages with chronological age in Thai between cleft lip and palate and non-cleft patients*. Journal of the Medical Association of Thailand, 96*(4), 9–18. | CVM stage not based on Baccetti’s method (2005) |
| 2013 | Goyal, S., & Goyal, S. (2013). Assessment of skeletal maturation and pubertal growth spurt using cervical vertebrae maturation indicators. *Rwanda Medical Journal, 70*(3), 28-33. | CVM stage not based on Baccetti’s method (2005) |
| 2014 | Majeed, O., & Quadeer, T. A. (2014). Assessment of skeletal maturation and its correlation to chronological age using the cervical vertebral maturation method in a tertiary care hospital*. Journal Pakistan Dental Association, 23*(4), 153–158. | CVM stage not based on Baccetti’s method (2005) |
| 2014 | Carinhena, G., Siqueira, D. F., & Sannomiya, E. K. (2014). Skeletal maturation in individuals with Down's syndrome: comparison between PGS curve, cervical vertebrae and bones of the hand and wrist. *Dental press journal of orthodontics*, 19(4), 58–65. https://doi.org/10.1590/2176-9451.19.4.058-065.oar | CVM stage not based on Baccetti’s method (2005) |
| 2014 | Vichare, G. S., Kumar, P., & Sable, R. B. (2014). Interrelationship of Various Maturity Indicators during Adolescence in Maharashtrian Girls. *The Journal of Indian Orthodontic Society, 48*(1), 14–21. https://doi.org/10.5005/jp-journals-10021-1212 | CVM stage not based on Baccetti’s method (2005) |
| 2014 | Hedayati, Z., & Khalafinejad, F. (2014). Relationship between Body Mass Index, Skeletal Maturation and Dental Development in 6- to 15- Year Old Orthodontic Patients in a Sample of Iranian Population. Journal of Dentistry, 15(4), 180–186. | No mean or/and standard deviation or/and number of subjects values |
| 2014 | Perinetti, G., Caprioglio, A., & Contardo, L. (2014). Visual assessment of the cervical vertebral maturation stages: A study of diagnostic accuracy and repeatability. *The Angle orthodontist, 84*(6), 951–956. https://doi.org/10.2319/120913-906.1 | No mean or/and standard deviation or/and number of subjects values |
| 2014 | Pichai, S., Rajesh, M., Reddy, N., Adusumilli, G., Reddy, J., & Joshi, B. (2014). A comparison of hand wrist bone analysis with two different cervical vertebral analysis in measuring skeletal maturation. *Journal of international oral health, 6*(5), 36–41. | CVM stage not based on Baccetti’s method (2005) |
| 2014 | Crawford, B., Kim, D. G., Moon, E. S., Johnson, E., Fields, H. W., Palomo, J. M., & Johnston, W. M. (2014). Cervical vertebral bone mineral density changes in adolescents during orthodontic treatment. *American journal of orthodontics and dentofacial orthopedics, 146*(2), 183–189. https://doi.org/10.1016/j.ajodo.2014.04.019 | CVM stage not based on Baccetti’s method (2005) |
| 2014 | Santiago, R. C., Cunha, A. R., Júnior, G. C., Fernandes, N., Campos, M. J., Costa, L. F., Vitral, R. W., & Bolognese, A. M. (2014). New software for cervical vertebral geometry assessment and its relationship to skeletal maturation--a pilot study. *Dento maxillo facial radiology, 43*(2), 1-9. https://doi.org/10.1259/dmfr.20130238 | CVM stage not based on Baccetti’s method (2005) |
| 2014 | Santiago, R. C., Cunha, A. R., Júnior, G. C., Fernandes, N., Campos, M. J., Costa, L. F., Vitral, R. W., & Bolognese, A. M. (2014). New software for cervical vertebral geometry assessment and its relationship to skeletal maturation--a pilot study. Dento maxillo facial radiology, 43(2), 20130238. https://doi.org/10.1259/dmfr.20130238 | CVM stage not based on Baccetti’s method (2005) |
| 2014 | Perinetti, G., Perillo, L., Franchi, L., Di Lenarda, R., & Contardo, L. (2014). Maturation of the middle phalanx of the third finger and cervical vertebrae: a comparative and diagnostic agreement study*. Orthodontics & craniofacial research, 17*(4), 270–279. https://doi.org/10.1111/ocr.12052 | No mean or/and standard deviation or/and number of subjects values |
| 2014 | Rasool, G., Hussain, U., & Shah, S. S. (2014). Evaluation of the skeletal maturation using lower canine mineralization. *Pakistan Oral & Dental Journal, 34*(4), 629-634. | No mean or/and standard deviation or/and number of subjects values |
| 2014 | Padalino, S., Sfondrini, M. F., Chenuil, L., Scudeller, L., & Gandini, P. (2014). Reliability of skeletal maturity analysis using the cervical vertebrae maturation method on dedicated software. *International orthodontics, 12*(4), 483–493. https://doi.org/10.1016/j.ortho.2014.10.003 | CVM stage not based on Baccetti’s method (2005) |
| 2014 | Phelan, A., Franchi, L., Baccetti, T., Darendeliler, M. A., & McNamara, J. A., Jr (2014). Longitudinal growth changes in subjects with open-bite tendency: a retrospective study. *American journal of orthodontics and dentofacial orthopedics, 145(*1), 28–35. https://doi.org/10.1016/j.ajodo.2013.09.013 | No mean or/and standard deviation or/and number of subjects values |
| 2014 | Srivastava, M., Aggarwal, A., Batra, P., Datana, S., Kumar, P., & Macrusson, K. A. (2014). Association of cervical vertrebra anomalies with cleft lip and palate. *Journal of Cleft Lip Palate and Craniofacial Anomalies, 1*(1), 43-47. | CVM stage not based on Baccetti’s method (2005) |
| 2014 | Veli, I., Yuksel, B., & Uysal, T. (2014). Longitudinal evaluation of dental arch asymmetry in Class II subdivision malocclusion with 3-dimensional digital models. *American Journal of Orthodontics and Dentofacial Orthopedics, 145*(6), 763-770. | No mean or/and standard deviation or/and number of subjects values |
| 2014 | Yang, C., Qing, Z., Chun-tao, L., & Maimaitili, G. (2014). Comparison of mandibular length in patients with Class I and Class II skeletal patterns using the cervical vertebrae maturation. *Chinese Journal of Tissue Engineering Research, 18*(2), 187-192. | CVM stage not based on Baccetti’s method (2005) |
| 2014 | Nayak, S., Bhad Patil, W. A., & Doshi, U. H. (2014). The relationship between salivary insulin-like growth factor I and quantitative cervical maturational stages of skeletal maturity. *Journal of orthodontics, 41*(3), 170–174. https://doi.org/10.1179/1465313313Y.0000000091 | CVM stage not based on Baccetti’s method (2005) |
| 2014 | Elhaddaoui, R., Benyahia, H., Azaroual, F., & Zaoui, F. (2014). Intérêt de la méthode de maturation des vertèbres cervicales (CVM) en orthopédie dento-faciale : mise au point. *Revue de Stomatologie, de Chirurgie Maxillo-Faciale et de Chirurgie Orale, 115*(5), 293–300. | CVM stage not based on Baccetti’s method (2005) |
| 2014 | Yang, Y.-M., Lee, J., Kim, Y.-I., Cho, B.-H., & Park, S.-B. (2014). Axial cervical vertebrae-based multivariate regression model for the estimation of skeletal-maturation status. *Orthodontics & Craniofacial Research, 17*(3), 187–196. doi:10.1111/ocr.12045 | CVM stage not based on Baccetti’s method (2005) |
| 2014 | Silvestrini-Biavati, F., Ugolini, A., Laffi, N., Canevello, C., & Silvestrini-Biavati, A. (2014). Early diagnostic evaluation of mandibular symmetry using orthopantomogram. Indian journal of dental research, 25(2), 154–159. https://doi.org/10.4103/0970-9290.135909 | No mean or/and standard deviation or/and number of subjects values |
| 2014 | Veli, I., Yuksel, B., & Uysal, T. (2014). Longitudinal evaluation of dental arch asymmetry in Class II subdivision malocclusion with 3-dimensional digital models. *American journal of orthodontics and dentofacial orthopedics, 145*(6), 763–770. https://doi.org/10.1016/j.ajodo.2014.01.023 | No mean or/and standard deviation or/and number of subjects values |
| 2014 | Lione, R., Buongiorno, M., Franchi, L., & Cozza, P. (2014). Evaluation of maxillary arch dimensions and palatal morphology in mouth-breathing children by using digital dental casts. *International journal of pediatric otorhinolaryngology, 78*(1), 91–95. https://doi.org/10.1016/j.ijporl.2013.09.028 | No mean or/and standard deviation or/and number of subjects values |
| 2014 | Parenteau, C. S., Wang, N. C., Zhang, P., Caird, M. S., & Wang, S. C. (2014). Quantification of pediatric and adult cervical vertebra-anatomical characteristics by age and gender for automotive application. Traffic injury prevention, 15(6), 572–582. https://doi.org/10.1080/15389588.2013.843774 | CVM stage not based on Baccetti’s method (2005) |
| 2014 | Angelieri, F., Franchi, L., Cevidanes, L. H., Scanavini, M. A., & McNamara, J. A., Jr (2014). Long-term treatment effects of the FR-2 appliance: a prospective evalution 7 years post-treatment. *European journal of orthodontics, 36*(2), 192–199. https://doi.org/10.1093/ejo/cjt026 | No mean or/and standard deviation or/and number of subjects values |
| 2014 | Hassan, S., Shaikh, A., & Fida, M. (2014). Dental age and skeletal maturity stages in patients with impacted versus erupted maxillary canines. *Oral Health and Dental Management, 13(4)*, 945-949. | No mean or/and standard deviation or/and number of subjects values |
| 2014 | Carrera, M. C. G., Martínez, C. M., Díaz, Í. M., Mendoza, G. R. B., & Orozco, S. P. P. (2014). Estado de maduración ósea de las vértebras cervicales en una población colombiana con y sin labio y paladar fisurado. Universitas Odontológica, 33(70), 41-50. | No mean or/and standard deviation or/and number of subjects values |
| 2014 | Goyal, S., Goyal, S., & Gugnani, N. (2014). Assessment of skeletal maturity using the permanent mandibular canine calcification stages. *Journal of Orthodontic Research, 2*(1), 11. | CVM stage not based on Baccetti’s method (2005) |
| 2014 | Cossellu, G., Biagi, R., Pisani, L., Barbieri, V., & Farronato, G. (2014). Relationship between mandibular second molar calcification stages and cervical vertebrae maturity in Italian children and young adults. *European Journal of Paediatric Dentistry, 15*(4), 355-359. | CVM stage not based on Baccetti’s method (2005) |
| 2014 | Danaei, S. M., Karamifar, A., Sardarian, A., Shahidi, S., Karamifar, H., Alipour, A., & Ghodsi Boushehri, S. (2014). Measuring agreement between cervical vertebrae and hand-wrist maturation in determining skeletal age: reassessing the theory in patients with short stature. American journal of orthodontics and dentofacial orthopedics , 146(3), 294–298. https://doi.org/10.1016/j.ajodo.2014.05.023 | CVM stage not based on Baccetti’s method (2005) |
| 2014 | Ara, S. A., Arora, V., & Ullah, S. Z. (2014). Relationship between Dental maturity and Cervical Vertebral maturity in North Karnataka (India) children: A Hospital-based retrospective study. *Journal of Orofacial Research, 4*(1), 35-40. | CVM stage not based on Baccetti’s method (2005) |
| 2014 | Mujahid, R., Sakrani, H., Faisal, S. S., Samreen, A., & Mirza, D. (2014). Average chronological age & Occurence of pubertal spurt assessed by CVM method in Pakistani girls and boys. *Pakistan Oral & Dental journal, 34*(4), 640-643. | CVM stage not based on Baccetti’s method (2005) |
| 2014 | Goyal, S., Goyal, S., & Gugnani, N. (2014). Assessment of skeletal maturation using mandibular second molar maturation stages. *The Journal of clinical pediatric dentistry, 39*(1), 79–84. https://doi.org/10.17796/jcpd.39.1.9224022173577151 | CVM stage not based on Baccetti’s method (2005) |
| 2015 | De Fuentes, A. M., López, J., Jiménez, J. F., Ruidíaz, V., & Romero, R. M. D. (2015). Correlation between cervical and hand-wrist analysis for skeletal maturation in Mexican boys and girls of the children’s General Hospital of Mexico «Federico Gomez». Revista Mexicana de Ortodoncia, 3(4), 232–237. https://doi.org/10.1016/j.rmo.2016.03.062 | CVM stage not based on Baccetti’s method (2005) |
| 2015 | Román, J. E. P., Bermúdez, O. M., Pombo, J. L., Mardínis, T. F., Edna, M. E., & Herrera, A. H. (2015). Determination of the stages of skeletal maturation by analysis Bacceti. Salud Uninorte, 31(2), 228–233. https://doi.org/10.14482/sun.31.2.6495 | No mean or/and standard deviation or/and number of subjects values |
| 2015 | Gupta, S., Mehendiratta, M., Rehani, S., Kumra, M., Nagpal, R., & Gupta, R. (2015). Age estimation in Indian children and adolescents in the NCR region of Haryana: A comparative study. Journal of forensic dental sciences, 7(3), 253–258. https://doi.org/10.4103/0975-1475.172453 | CVM stage not based on Baccetti’s method (2005) |
| 2015 | Safavi, S. M., Beikaii, H., Hassanizadeh, R., Younessian, F., & Baghban, A. A. (2015). Correlation between cervical vertebral maturation and chronological age in a group of Iranian females. Dental research journal, 12(5), 443–448. https://doi.org/10.4103/1735-3327.166192 | No mean or/and standard deviation or/and number of subjects values |
| 2015 | Chongcharueyskul, P., Wangsrimonkol, T., Pisek, P., Pisek, A., & Manosudprasit, M. (2015). Correlations between cervical vertebral maturation (CVM) and dental development in Thai cleft patients. *Journal of the Medical Association of Thailand*, *98*(7), 92-100. | No mean or/and standard deviation or/and number of subjects values |
| 2015 | Ravadgar, M., Mirshekar, A., Moudi, E., & Bijani, A. (2015). Association Between Teeth Development in Panoramic Radiograph and Skeletal Maturity in Lateral Cephalogram. Avicenna Journal of Dental Research, 7(2), 1-8. https://doi.org/10.5812/ajdr.21202 | No mean or/and standard deviation or/and number of subjects values |
| 2015 | Byun, B. R., Kim, Y. I., Yamaguchi, T., Maki, K., & Son, W. S. (2015). Quantitative assessment of cervical vertebral maturation using cone beam computed tomography in Korean girls. *Computational and mathematical methods in medicine, 2015*, 1-9. https://doi.org/10.1155/2015/405912 | CVM stage not based on Baccetti’s method (2005) |
| 2015 | Dzemidzic, V., Sokic, E., Tiro, A., & Nakas, E. (2015). Computer Based Assessment of Cervical Vertebral Maturation Stages Using Digital Lateral Cephalograms. *Acta informatica medica, 2*3(6), 364–368. https://doi.org/10.5455/aim.2015.23.364-368 | CVM stage not based on Baccetti’s method (2005) |
| 2015 | Mamillapalli, P. K., Sesham, V. M., Neela, P. K., Kondapaka, V., & Mandaloju, S. P. (2015). A smartphone app for identifying cervical vertebral maturation stages. *Journal of clinical orthodontics, 49*(9), 582–585. | No mean or/and standard deviation or/and number of subjects values |
| 2015 | Byun, B. R., Kim, Y. I., Yamaguchi, T., Maki, K., Ko, C. C., Hwang, D. S., Park, S. B., & Son, W. S. (2015). Quantitative skeletal maturation estimation using cone-beam computed tomography-generated cervical vertebral images: a pilot study in 5- to 18-year-old Japanese children. *Clinical oral investigations, 19*(8), 2133–2140. https://doi.org/10.1007/s00784-015-1415-6 | CVM stage not based on Baccetti’s method (2005) |
| 2015 | Wiwatworakul, O., Supaporn Chatrchaiwiwatana, D. D. S., & Tasanee Wangsrimongkol, D. D. S. (2015). Agreement of Tracing and Direct Viewing Techniques for Cervical Vertebral Maturation Assessment. *Journal of the Medical Association of Thailand, 98*(7), 77-83. | No mean or/and standard deviation or/and number of subjects values |
| 2015 | Predko-Engel, A., Kaminek, M., Langova, K., Kowalski, P., & Fudalej, P. S. (2015). Reliability of the cervical vertebrae maturation (CVM) method. *Bratislavske lekarske listy, 11*6(4), 222–226. https://doi.org/10.4149/bll_2015_043 | No mean or/and standard deviation or/and number of subjects values |
| 2015 | Tayyab, M., Hussain, U., Ali, M., Ayub, A., & Hadi, F. (2015). Evaluation of Mandibular lenght in subjetcs with Class I and Class II Skeletal Patterns Using the Cervical Vertebrae Maturation. *Pakistan Oral & Dental Journal, 35*(1), 74-78. | No mean or/and standard deviation or/and number of subjects values |
| 2015 | Cameriere, R., Giuliodori, A., Zampi, M., Galić, I., Cingolani, M., Pagliara, F., & Ferrante, L. (2015). Age estimation in children and young adolescents for forensic purposes using fourth cervical vertebra (C4). *International journal of legal medicine, 129*(2), 347–355. https://doi.org/10.1007/s00414-014-1112-z | No mean or/and standard deviation or/and number of subjects values |
| 2015 | Altan, A. B., Demiturk, H. K., Sinanoglu, E. A., & Mutaf, H. I. (2015). The evaluation of the relationship among various maturation indicators in Turkish subjects. *Cumhuriyet Dental Journal, 18*(3), 235-248. | CVM stage not based on Baccetti’s method (2005) |
| 2015 | Phogat, R., Sidhu, M. S., Grover, S., Dabas, A., Malik, V., & Diwakar, R. (2015). Comparative evaluation of efficiency of serum IGF-1, hand-wrist radiographs, and cervical vertebrae as skeletal maturity indicator. *Journal of Indian Orthodontic Society, 49*(4), 199-205. | No mean or/and standard deviation or/and number of subjects values |
| 2015 | Torun S. G., & Oktay, H. (2015). Evaluation of the relationships between chronological age, skeletal maturation, dental maturation, and sagittal jaw relationships. *Turkish Journal of Orthodontics, 28*(3), 86-91. | No mean or/and standard deviation or/and number of subjects values |
| 2015 | Altan, B., Sinanoglu, A., & Kocasaraç, H. D. (2015). The assessment of cervical vertebral anomalies on lateral cephalograms and cone-beam computed tomographs. *Cumhuriyet Dental Journal, 18*(4), 327-334. | CVM stage not based on Baccetti’s method (2005) |
| 2015 | Sharma, K., Kahlon, S. S., Boparai, C. S., Mehta, V., Jassal, N. S., & Sandhu, A. S. (2015). Correlation of skeletal maturation using CVMI with dental calcification using Willem's method. *Indian Journal of Comprehensive Dental Care, 5*(2), 612–616. | CVM stage not based on Baccetti’s method (2005) |
| 2015 | Arriola-Guillén, L. E., Fitzcarrald, F. D., & Flores-Mir, C. (2015). Semi-longitudinal Study of the Mcnamara Cephalometric Triangle in Class II and Class III Subjects Grouped by Cervical Vertebrae Maturation Stage. *Acta odontologica latinoamericana, 28*(3), 222–230. | No mean or/and standard deviation or/and number of subjects values |
| 2015 | Lazić, E., Glišić, B., Stamenković, Z., & Nedeljković, N. (2015). Changes in Cervical Lordosis and Cervicovertebral Morphology in Different Ages with the Possibility of Estimating Skeletal Maturity. Srpski arhiv za celokupno lekarstvo, 143(12), 662–668. https://doi.org/10.2298/sarh1512662l | No mean or/and standard deviation or/and number of subjects values |
| 2015 | Singh, S., Sandhu, N., Puri, T., Gulati, R., & Kashyap, R. (2015). A Study of Correlation of Various Growth Indicators with Chronological Age. International journal of clinical pediatric dentistry, 8(3), 190–195. https://doi.org/10.5005/jp-journals-10005-1311 | CVM stage not based on Baccetti’s method (2005) |
| 2015 | Deen, E., & Woods, M. G. (2015). Effects of the Herbst appliance in growing orthodontic patients with different underlying vertical patterns. *Australian orthodontic journal, 31*(1), 59–68. | CVM stage not based on Baccetti’s method (2005) |
| 2015 | Caprioglio, A., Finazzi, F., Mortellaro, C., Mangano, C., Lucchina, A. G., Mangano, F., & Levrini, L. (2015). Predictive variables derived from panoramic radiographs for impacted maxillary cuspids treated with easy cuspid system. The Journal of craniofacial surgery, 26(3), 714–718. https://doi.org/10.1097/SCS.0000000000001506 | No mean or/and standard deviation or/and number of subjects values |
| 2015 | Rongo, R., Valleta, R., Bucci, R., Bonetti, G. A., Michelotti, A., & D'Antò, V. (2015). Does clinical experience affect the reproducibility of cervical vertebrae maturation method?. The Angle orthodontist, 85(5), 841–847. https://doi.org/10.2319/080414-544.1 | No mean or/and standard deviation or/and number of subjects values |
| 2015 | Servello, D. F., Fallis, D. W., & Alvetro, L. (2015). Analysis of Class II patients, successfully treated with the straight-wire and Forsus appliances, based on cervical vertebral maturation status. The Angle orthodontist, 85(1), 80–86. https://doi.org/10.2319/102513-780.1 | No mean or/and standard deviation or/and number of subjects values |
| 2015 | Shin, S. M., Kim, Y. I., Choi, Y. S., Yamaguchi, T., Maki, K., Cho, B. H., & Park, S. B. (2015). The skeletal maturation status estimated by statistical shape analysis: axial images of Japanese cervical vertebra. Dento maxillo facial radiology, 44(3). https://doi.org/10.1259/dmfr.20140323 | CVM stage not based on Baccetti’s method (2005) |
| 2015 | Veli, I., Ozturk, M. A., & Uysal, T. (2015). Development of the curve of Spee in Class II subdivision malocclusion: a longitudinal study. European journal of orthodontics, 37(4), 412–417. https://doi.org/10.1093/ejo/cju062 | No mean or/and standard deviation or/and number of subjects values |
| 2015 | Lee, K. M., Chung, D. H., Lee, J. W., & Lee, S. M. (2015). Cervical vertebrae maturation, dentoalveolar, head postural and respiratory parameters in predicting the stable outcome of face-mask treatment. European journal of orthodontics, 37(3), 251–256. https://doi.org/10.1093/ejo/cju035 | No mean or/and standard deviation or/and number of subjects values |
| 2015 | El-Kabbany, S., Ibrahim, S., Salama, A., & Baumrind, S. (2015). Evaluation of craniofacial growth changes in subjects with class II and class III malocclusions during the circum-pubertal period. *Egyptian Orthodontic Journal, 47*, 67-92. | No mean or/and standard deviation or/and number of subjects values |
| 2015 | Masoud, M. I., Marghalani, H. Y., Bamashmous, M., Alamoudi, N. M., El Derwi, D., Masoud, I. M., Allareddy, V., & Gowharji, N. F. (2015). Predicting changes in mandibular length and total anterior facial height using IGF-1, cervical stage, skeletal classification, and gender. *Progress in orthodontics, 16*(1), 1-6. https://doi.org/10.1186/s40510-015-0076-y | No mean or/and standard deviation or/and number of subjects values |
| 2015 | Mustafa, S., Raj, A. C., Anekar, J., Divakar, D. D., Al Kheraif, A. A., Ramakrishnaiah, R., Khan, A. A., Alshahrani, O. A., & Rai, N. P. (2015). Evaluation of dental and skeletal maturity using digital panoramic radiographs and digital cephalograms. *Asian Biomed, 9(*3), 335-342. | No mean or/and standard deviation or/and number of subjects values |
| 2015 | Flieger, R., Kopczyński, P., & Matthews-Brzozowska, T. (2015). Analysis of skeletal maturity of children with cleft palate in terms of healthy children by the CVM method. *Advances in clinical and experimental medicine, 24*(1), 99-102. | No mean or/and standard deviation or/and number of subjects values |
| 2015 | Gupta, S., Deoskar, A., Gupta, P., & Jain, S. (2015). Serum insulin-like growth factor-1 levels in females and males in different cervical vertebral maturation stages. *Dental press journal of orthodontics*, 20(2), 68-75. | No mean or/and standard deviation or/and number of subjects values |
| 2015 | Srkoč, T., Meštrović, S., Anić-Milošević, S., & Šlaj, M. (2015). Association between dental and skeletal maturation stages in Croatian subjects. *Acta clinica Croatica, 54*(4.), 445-452. | CVM stage not based on Baccetti’s method (2005) |
| 2015 | Angelieri, F., Franchi, L., Cevidanes, L. H., & McNamara, J. A., Jr (2015). Diagnostic performance of skeletal maturity for the assessment of midpalatal suture maturation. *American journal of orthodontics and dentofacial orthopedics, 148*(6), 1010–1016. https://doi.org/10.1016/j.ajodo.2015.06.016 | CVM stage not based on Baccetti’s method (2005) |
| 2015 | Baba, I. A., Shah, A. F., Shahnaz, N., Yousuf, A., Adhnan, M. F., & Kanji, M. (2015). Correlation between dental maturity and cervical certebral maturity amongst 7-15 year old Kashmiri children. *Schoulars Journal of Dental Science, 2*(3), 259-264. | CVM stage not based on Baccetti’s method (2005) |
| 2015 | Akarsu-Guven, B., Karakaya, J., Ozgur, F., & Aksu, M. (2015). Growth-related changes of skeletal and upper-airway features in bilateral cleft lip and palate patients. *American journal of orthodontics and dentofacial orthopedics, 148*(4), 576–586. https://doi.org/10.1016/j.ajodo.2015.02.031 | CVM stage not based on Baccetti’s method (2005) |
| 2016 | Cericato, G. O., Franco, A., Bittencourt, M. A., Nunes, M. A., & Paranhos, L. R. (2016). Correlating skeletal and dental developmental stages using radiographic parameters. *Journal of forensic and legal medicine, 42*, 13–18. https://doi.org/10.1016/j.jflm.2016.05.009 | No mean or/and standard deviation or/and number of subjects values |
| 2016 | Jeelani, W., Fida, M., & Shaikh, A. (2016). The duration of pubertal growth peak among three skeletal classes. Dental press journal of orthodontics, 21(5), 67–74. https://doi.org/10.1590/2177-6709.21.5.067-074.oar | No mean or/and standard deviation or/and number of subjects values |
| 2016 | Bonfim, M. A., Costa, A. L., Fuziy, A., Ximenez, M. E., Cotrim-Ferreira, F. A., & Ferreira-Santos, R. I. (2016). Cervical vertebrae maturation index estimates on cone beam CT: 3D reconstructions vs sagittal sections. Dento maxillo facial radiology, 45(1), 20150162. https://doi.org/10.1259/dmfr.20150162 | CVM stage not based on Baccetti’s method (2005) |
| 2016 | Džemidžić, V., Tiro, A., Zukanović, A., Redžić, I., & Nakaš, E. (2016). Skeletal maturity assessment using mandibular canine calcification stages. Acta medica academica, 45(2), 128–134. https://doi.org/10.5644/ama2006-124.168 | CVM stage not based on Baccetti’s method (2005) |
| 2016 | Mehta, N., Patel, D., Mehta, F., Gupta, B., Zaveri, G., & Shah, U. (2016). Evaluation of skeletal maturation using mandibular third molar development in Indian adolescents. Journal of forensic dental sciences, 8(2), 112. https://doi.org/10.4103/0975-1475.186372 | CVM stage not based on Baccetti’s method (2005) |
| 2016 | Jeelani, W., Fida, M., Shaikh, A. (2016). The duration of pubertal growth peak among three skeletal classes. Dental Press Journal of Orthodontics, 21(5), 67–74. https://doi.org/10.1590/2177-6709.21.5.067-074.oar | No mean or/and standard deviation or/and number of subjects values |
| 2016 | Sinha, M., Tripathi, T., Rai, P., & Gupta, S. K. (2016). Serum and urine insulin-like growth factor-1 as biochemical growth maturity indicators. American Journal of Orthodontics and Dentofacial Orthopedics, 150(6), 1020–1027. 10.1016/j.ajodo.2016.04.028 | CVM stage not based on Baccetti’s method (2005) |
| 2016 | Rainey, B. J., Burnside, G., & Harrison, J. E. (2016). Reliability of cervical vertebral maturation staging. *American journal of orthodontics and dentofacial orthopedics, 150*(1), 98–104. https://doi.org/10.1016/j.ajodo.2015.12.013 | No mean or/and standard deviation or/and number of subjects values |
| 2016 | Perinetti, G., Contardo, L., Castaldo, A., McNamara, J. A., Jr, & Franchi, L. (2016). Diagnostic reliability of the cervical vertebral maturation method and standing height in the identification of the mandibular growth spurt. *The Angle orthodontist, 86*(4), 599–609. https://doi.org/10.2319/072415-499.1 | CVM stage not based on Baccetti’s method (2005) |
| 2016 | Mahmood, H. T., Shaikh, A., & Fida, M. (2016). Association between frontal sinus morphology and cervical vertebral maturation for the assessment of skeletal maturity. American journal of orthodontics and dentofacial orthopedics, 150(4), 637–642. https://doi.org/10.1016/j.ajodo.2016.03.022 | No mean or/and standard deviation or/and number of subjects values |
| 2016 | Gray, S., Bennani, H., Kieser, J. A., & Farella, M. (2016). Morphometric analysis of cervical vertebrae in relation to mandibular growth. *American journal of orthodontics and dentofacial orthopedics, 149*(1), 92–98. https://doi.org/10.1016/j.ajodo.2015.06.028 | No mean or/and standard deviation or/and number of subjects values |
| 2016 | Yastani, D., Purbiati, M., & Astuti, I.A. (2016). Dentocraniofacial morphology in unilateral and bilateral cleft lip and palate following labioplasty and palatoplasty; analysis at first and second cervical vertebral maturation stage. Journal of International Dental and Medical Research, 9, 282-286. | CVM stage not based on Baccetti’s method (2005) |
| 2016 | Sohrabi, A., Babay Ahari, S., Moslemzadeh, H., Rafighi, A., & Aghazadeh, Z. (2016). The reliability of clinical decisions based on the cervical vertebrae maturation staging method. European journal of orthodontics, 38(1), 8–12. https://doi.org/10.1093/ejo/cjv030 | CVM stage not based on Baccetti’s method (2005) |
| 2016 | Fernández-Pérez, M. J., Alarcón, J. A., McNamara, J. A., Jr, Velasco-Torres, M., Benavides, E., Galindo-Moreno, P., & Catena, A. (2016). Spheno-Occipital Synchondrosis Fusion Correlates with Cervical Vertebrae Maturation. PloS one, 11(8), 1-11. https://doi.org/10.1371/journal.pone.0161104 | No mean or/and standard deviation or/and number of subjects values |
| 2016 | Engel, T. P., Renkema, A. M., Katsaros, C., Pazera, P., Pandis, N., & Fudalej, P. S. (2016). The cervical vertebrae maturation (CVM) method cannot predict craniofacial growth in girls with Class II malocclusion. *European journal of orthodontics, 38*(1), 1–7. https://doi.org/10.1093/ejo/cju085 | No mean or/and standard deviation or/and number of subjects values |
| 2016 | Hoseini, M., Zamaheni, S., Bashizadeh Fakhar, H., Akbari, F., Chalipa, J., & Rahmati, A. (2016). Comparative Evaluation of the Efficacy of Hand-Wrist and Cervical Vertebrae Radiography for the Determination of Skeletal Age. *Iranian journal of radiology, 13*(3), e21695. https://doi.org/10.5812/iranjradiol.21695 | CVM stage not based on Baccetti’s method (2005) |
| 2016 | DuPlessis, E. A., Araujo, E. A., Behrents, R. G., & Kim, K. B. (2016). Relationship between body mass and dental and skeletal development in children and adolescents. *American journal of orthodontics and dentofacial orthopedics, 150*(2), 268–273. https://doi.org/10.1016/j.ajodo.2015.12.031 | CVM stage not based on Baccetti’s method (2005) |
| 2016 | Kumar, S., Agarwal, N., & Mehrotra, A. K. (2016). Evaluation of skeletal maturity in North Indian subjects using an objective method based on cervical vertebral bone age and assessment of its reliability as compared to hand wrist radiographic method. *Journal of Indian Orthodontic Society, 50*(1), 3-7. | CVM stage not based on Baccetti’s method (2005) |
| 2016 | Cossellu, G., Farronato, G., Nicotera, O., & Biagi, R. (2016). Transverse maxillary deficit and its influence on the cervical vertebrae maturation index. *European journal of paediatric dentistry, 17*(2), 147–150. | CVM stage not based on Baccetti’s method (2005) |
| 2016 | Torun, G. S. (2016). Soft tissue changes in the orofacial region after rapid maxillary expansion. *Journal of Orofacial Orthopedics,* 78(3), 193–200. doi:10.1007/s00056-016-0074-9 | No mean or/and standard deviation or/and number of subjects values |
| 2016 | Moradinejad, M., Berahman, N., Sadeghi, E., & Javidi, P. (2016). Determination of developmental stage of cervical vertebrae at menarche in female orthodontic patients in Ahwaz. *International Journal of Medical Research & Health Sciences, 5*(8), 206-210. | No mean or/and standard deviation or/and number of subjects values |
| 2016 | Khalidi, F., Hegde, C., Sahaf, N., Darsan, J., & Roy, A. (2016). Variability of Subjective v/s Objective Method of determination of skeletal age. *Journal Of Applied Dental and Medical Sciences, 2*(2), 24-31. | CVM stage not based on Baccetti’s method (2005) |
| 2016 | Litsas, G., Athanasiou, A. E., Papadopoulos, M. A., Ioannidou-Marathiotou, I., & Karagiannis, V. (2016). Dental calcification stages as determinants of the peak growth period. *Journal of orofacial orthopedics, 77*(5), 341–349. https://doi.org/10.1007/s00056-016-0040-6 | CVM stage not based on Baccetti’s method (2005) |
| 2016 | Jeelani, W., Fida, M., & Shaikh, A. (2016). The Onset and Duration of Pubertal Growth Spurt among Three Skeletal Malocclusions. *Journal of Pioneering Medical Sciences, 6*(2), 80. | No mean or/and standard deviation or/and number of subjects values |
| 2016 | Perinetti, G., & Contardo, L. (2016). Gingival crevicular fluid alkaline phosphatase activity in relation to pubertal growth spurt and dental maturation: a multiple regression study. *South European Journal of Orthodontics and Dentofacial Research, 3*(1), 6-11. | No mean or/and standard deviation or/and number of subjects values |
| 2016 | Meghana, H. C., Charan, K. S., Pramod, G. V., & Ashok, L. (2016). Radiographic Comparisons between Middle Phalanx of the Third Finger and Cervical Vertebrae Maturation for the Assessment of Skeletal Maturity. *Journal of Forensic Science and Medicine, 2*(3), 141-145. | CVM stage not based on Baccetti’s method (2005) |
| 2016 | Krishnamoorthy, V. V., Parameswaran, R., Vijayalakshmi, D., Khan, N., & Nandakumar, A. (2016). Assessment of Skeletal Maturation in Concordance to Statural Height and Body Weight in 12-Year-Old Children - A Cross-Sectional Study. *Journal of clinical and diagnostic research, 10*(6), 51-55. | No mean or/and standard deviation or/and number of subjects values |
| 2016 | Jayachandran, S., Wiltshire, W. A., Hayasaki, S. M., & Pinheiro, F. H. (2016). Comparison of AdvanSync and intermaxillary elastics in the correction of Class II malocclusions: A retrospective clinical study. American journal of orthodontics and dentofacial orthopedics, 150(6), 979–988. https://doi.org/10.1016/j.ajodo.2016.05.008 | No mean or/and standard deviation or/and number of subjects values |
| 2016 | Khoja, A., Fida, M., & Shaikh, A. (2016). Cephalometric evaluation of the effects of the Twin Block appliance in subjects with Class II, Division 1 malocclusion amongst different cervical vertebral maturation stages. *Dental press journal of orthodontics, 21*(3), 73–84. https://doi.org/10.1590/2177-6709.21.3.073-084.oar | No mean or/and standard deviation or/and number of subjects values |
| 2016 | Choi, Y. K., Kim, J., Yamaguchi, T., Maki, K., Ko, C. C., & Kim, Y. I. (2016). Cervical Vertebral Body's Volume as a New Parameter for Predicting the Skeletal Maturation Stages. BioMed research international, 2016, 1–7. https://doi.org/10.1155/2016/8696735 | CVM stage not based on Baccetti’s method (2005) |
| 2016 | Pakbaznejad, E., Hurmerinta, K., Rice, D., & Suomalainen, A. (2016). Ultrasonographic localization of the thyroid gland for its optimal shielding prior to lateral cephalometric radiography: a pilot study. Dento maxillo facial radiology, 45(3),1-6. https://doi.org/10.1259/dmfr.20150341 | No mean or/and standard deviation or/and number of subjects values |
| 2016 | Šidlauskas, M., Šalomskienė, L., Andriuškevičiūtė, I., Šidlauskienė, M., Labanauskas, Ž., Vasiliauskas, A., Kupčinskas, L., Juzėnas, S., & Šidlauskas, A. (2016). Heritability of mandibular cephalometric variables in twins with completed craniofacial growth. *European journal of orthodontics, 38*(5), 493–502. https://doi.org/10.1093/ejo/cjv062 | No mean or/and standard deviation or/and number of subjects values |
| 2016 | Trakinienė, G., Smailienė, D., & Kučiauskienė, A. (2016). Evaluation of skeletal maturity using maxillary canine, mandibular second and third molar calcification stages. European journal of orthodontics, 38(4), 398–403. https://doi.org/10.1093/ejo/cjv051 | No mean or/and standard deviation or/and number of subjects values |
| 2016 | Jang, H. I., Kim, S. C., Chae, J. M., Kang, K. H., Cho, J. W., Chang, N. Y., Lee, K. Y., & Cho, J. H. (2016). Relationship between maturation indices and morphology of the midpalatal suture obtained using cone-beam computed tomography images. *Korean journal of orthodontics, 46*(6), 345–355. https://doi.org/10.4041/kjod.2016.46.6.345 | No mean or/and standard deviation or/and number of subjects values |
| 2016 | Mokhtari, N., & Ghafari, H. A. (2016). Determining Chronological Age for the Treatment of Class I, II and III Malocclusion in the Crowd of Iranians. *Biomedical & Pharmacology Journal, 9*(3), 1079-1085. | No mean or/and standard deviation or/and number of subjects values |
| 2016 | Kishore, M. S. V., Sravya, R. L., Dasari, A. K., Varalakshmi, C., & Vishal, G. (2016). Diagnostic potential of mandibular second premolar and its relation to physiological and skeletal maturity in girls. *International Journal of Dentistry and Oral Science, 3*(7), 291-295. | CVM stage not based on Baccetti’s method (2005) |
| 2016 | Suresh, M., Patil, B. C., Patil, V. C., Halkai, S., Swathi, B., & Bansal, A. (2016). Assessment of Skeletal and Dental Maturation in Different Facial Types of South Indian Population–A Comparative Study. *Journal of Dental and Medical Sciences, 15*(8), 122-129. | No mean or/and standard deviation or/and number of subjects values |
| 2016 | Torres, E. A., Gutierrez-Rojo, J. F., & Rivas-Gutiérrez, R. (2016). Evaluación del método de maduración ósea de las vértebras cervicales de Baccetti en pacientes de 8 a 15 años. *Acta Odontológica Colombiana, 6*(1), 83-89. | No mean or/and standard deviation or/and number of subjects values |
| 2016 | Jeelani, W., Fida, M., & Shaikh, A. (2016). Timing of adolescent growth spurt among children with different skeletal classes. *Pakistan Orthodontic Journal*, 8(2), 72-79. | No mean or/and standard deviation or/and number of subjects values |
| 2016 | Litsas, G., & Lucchese, A. (2016). Dental and Chronological Ages as Determinants of Peak Growth Period and Its Relationship with Dental Calcification Stages. *The open dentistry journal, 10*, 99–108. https://doi.org/10.2174/1874210601610010099 | CVM stage not based on Baccetti’s method (2005) |
| 2017 | Rahmawati, A. D., Ahmad, I., & Setyawan, A. S. (2017). The role of cervical vertebrae maturation in defining the chronological age of Down syndrome children. *Dental Journal Majalah Kedokteran Gigi, 50*(4), 220-225. https://doi.org/10.20473/j.djmkg.v50.i4.p220-225 | No mean or/and standard deviation or/and number of subjects values |
| 2017 | Tayebi, A., Tofangchiha, M., Fard, M. A., & Gosili, A. (2017). The relationship of mandibular radiomorphometric indices to skeletal age, chronological age and skeletal malocclusion type. Journal of clinical and experimental dentistry, 9(8), 970–975. https://doi.org/10.4317/jced.53819 | CVM stage not based on Baccetti’s method (2005) |
| 2017 | Lecca-Morales, R. M., & Carruitero, M. J. (2017). Relationship between dental calcification and skeletal maturation in a Peruvian sample. *Dental press journal of orthodontics, 22*(3), 89–96. https://doi.org/10.1590/2177-6709.22.3.089-096.oar | No mean or/and standard deviation or/and number of subjects values |
| 2017 | Verulkar, A., Singla, P., Patil, H., & Tekale, P. (2017). Assessment of skeletal and dental maturity indicators and comparison of maturity indicators in vertical and horizontal growth pattern individuals with normal growth pattern individuals. *International Journal of Orthodontic Rehabilitation, 8*(3), 108-111. https://doi.org/10.4103/ijor.ijor_20_17 | CVM stage not based on Baccetti’s method (2005) |
| 2017 | Mehta, V., & Pandey, R. K. (2017). An evaluation of craniofacial growth pattern in North Indian children. *Journal of oral biology and craniofacial research*, *7*(1), 27–31. https://doi.org/10.1016/j.jobcr.2016.12.001 | CVM stage not based on Baccetti’s method (2005) |
| 2017 | Nemati, S., Azimi, F., Rouhi, M., & Tafakhori, Z. (2017). The Relationship between Dental Maturity based on Demirjian’s Method and Cervical Vertebrae Maturation Stages in Guilan patients. *Journal of Dentomaxillofacial Radiology, Pathology and Surgery, 6*(2), 1-9. | No mean or/and standard deviation or/and number of subjects values |
| 2017 | Cisternas, A., Morales, R., Ramirez, V., Real, A., & Oyonarte, R. (2017). Diagnostic assessment of skeletal maturity through dental maturation in Hispanic growing individuals. APOS Trends in Orthodontics, 7(1), 35–40. https://doi.org/10.4103/2321-1407.199181 | No mean or/and standard deviation or/and number of subjects values |
| 2017 | Kang, J. H., Yang, I. H., Hyun, H. K., & Lee, J. Y. (2017). Dental and skeletal maturation in female adolescents with temporomandibular joint osteoarthritis. Journal of oral rehabilitation, 44(11), 879–888. https://doi.org/10.1111/joor.12547 | CVM stage not based on Baccetti’s method (2005) |
| 2017 | Camacho-Basallo, P., Yáñez-Vico, R. M., Solano-Reina, E., & Iglesias-Linares, A. (2017). Five radiographic methods for assessing skeletal maturity in a Spanish population: is there a correlation?. Acta odontologica Scandinavica, 75(2), 106–112. | No mean or/and standard deviation or/and number of subjects values |
| 2017 | Kim, S. H., Choi, Y. K., Shin, S. M., Choi, Y. S., Yamaguchi, T., Takahashi, M., Maki, K., Park, S. B., & Kim, Y. I. (2017). The estimation of skeletal maturity of patients with cleft lip and palate using statistical shape analysis: a preliminary study. Dento maxillo facial radiology, 46(5), 1-8. | No mean or/and standard deviation or/and number of subjects values |
| 2017 | Mânica, S., & Liversidge, H. M. (2017). Accuracy of estimating age from cervical vertebral maturation and mandibular molar maturation. Revista Brasileira de Odontologia Legal, 4(1), 2-10. https://doi.org/10.21117/rbol.v4i1.77 | CVM stage not based on Baccetti’s method (2005) |
| 2017 | Mulett Vasquez, J., & Parra Sánchez, H. (2017). Ossification characteristics of the hand and the cervical vertebrae and correlation between the two techniques as indicators of somatic maturation in children from 8 to 17 years. Revista Estomatología, 20(2), 7–15. https://doi.org/10.25100/re.v20i2.5736 | CVM stage not based on Baccetti’s method (2005) |
| 2017 | Shaaban, A. A. E., & El-Shall, O. S. A. (2017). Age estimation based on some cervical vertebral measurements in a sample of Egyptian children. Ain Shams Journal of Forensic Medicine and Clinical Toxicology, 28(1), 72-87. | CVM stage not based on Baccetti’s method (2005) |
| 2017 | Grünheid, T., Larson, C. E., & Larson, B. E. (2017). Midpalatal suture density ratio: A novel predictor of skeletal response to rapid maxillary expansion. *American journal of orthodontics and dentofacial orthopedics, 151(*2), 267–276. https://doi.org/10.1016/j.ajodo.2016.06.043 | CVM stage not based on Baccetti’s method (2005) |
| 2017 | Montasser, M. A., Viana, G., & Evans, C. A. (2017). Racial and sex differences in timing of the cervical vertebrae maturation stages. *American journal of orthodontics and dentofacial orthopedics, 151*(4), 744–749. https://doi.org/10.1016/j.ajodo.2016.09.019 | CVM stage not based on Baccetti’s method (2005) |
| 2017 | Baldin, C., Kitt, M., Costa, A., Yasuda, C., Cendes, F., & Nahás-Scocate, A. (2017). Evaluation of the Skeletal Maturation of Cervical Vertebrae with Magnetic Resonance Imaging: a pilot study. *Brazilian Journal Of Oral Sciences, 16, 1-8*. doi:10.20396/bjos.v16i1.8650501 | CVM stage not based on Baccetti’s method (2005) |
| 2017 | Gelbrich, B., Fischer, M., Stellzig-Eisenhauer, A., & Gelbrich, G. (2017). Are cervical vertebrae suitable for age estimation? *The Journal of forensic odonto-stomatology, 35*(2), 66–78. | CVM stage not based on Baccetti’s method (2005) |
| 2017 | Nanda, M., Singla, A., Sachdev, V., & Jaj, H. S. (2017). Correlation of Chronological, Skeletal, and Dental Age in North Indian Population. *Indian Journal of Dental Sciences, 9*(5), 13-20. | CVM stage not based on Baccetti’s method (2005) |
| 2017 | Carruitero, M. J. (2017). Correlation between the skeletal maturation stages of two radiographic methods in Peruvians. *Journal of International Oral Health, 9*(6), 289-292. | CVM stage not based on Baccetti’s method (2005) |
| 2017 | Türkoz, Ç., Kaygısız, E., Ulusoy, Ç., & Ateş, C. (2017). A practical formula for determining growth. *Diagnostic and interventional radiology, 23*(3), 194–198. https://doi.org/10.5152/dir.2016.16334 | CVM stage not based on Baccetti’s method (2005) |
| 2017 | Tripathi, T., Gupta, P., Rai, P., Sharma, J., Gupta, V. K., & Singh, N. (2017). Osteocalcin and serum insulin-like growth factor-1 as biochemical skeletal maturity indicators. Progress in orthodontics, 18(1), 1-8. https://doi.org/10.1186/s40510-017-0184-y | CVM stage not based on Baccetti’s method (2005) |
| 2017 | Chen, M. Y., Liu, Y. T., & Hsu, W. H. (2017). Changes in the axial orientation of the zygapophyseal joint in the subaxial cervical spine from childhood to middle-age, and the biomechanical implications of these changes. *Journal of Clinical Neuroscience*, 44, 335–339. doi:10.1016/j.jocn.2017.06.072 | CVM stage not based on Baccetti’s method (2005) |
| 2017 | Fabiani, G., d'Apuzzo, F., Grassia, V., Laino, L., Femiano, F., & Perillo, L. (2017). Dentoskeletal features in mixed dentition children with displaced maxillary canines in a southern Italian population. *European journal of paediatric dentistry*, 18(2), 145–148. https://doi.org/10.23804/ejpd.2017.18.02.10 | No mean or/and standard deviation or/and number of subjects values |
| 2017 | Javangula, P. T., Uloopi, K. S., Vinay, C., Rayala, C., Kumar, N. M., & Chandra, S. P. (2017). Comparison of middle phalanx of the middle finger and cervical vertebrae as skeletal maturity indicators. *Indian Journal of Dental Sciences*, 9(2), 84. | CVM stage not based on Baccetti’s method (2005) |
| 2017 | Candir, M., & Kerosuo, H. (2017). Mode of correction is related to treatment timing in Class II patients treated with the mandibular advancement locking unit (MALU) appliance. *The Angle orthodontist, 87*(3), 363–370. https://doi.org/10.2319/071316-549.1 | No mean or/and standard deviation or/and number of subjects values |
| 2017 | Perinetti, G., Sbardella, V., & Contardo, L. (2017). Diagnostic reliability of the third finger middle phalanx maturation (MPM) method in the identification of the mandibular growth peak. *European journal of orthodontics, 39*(2), 194–201. https://doi.org/10.1093/ejo/cjw059 | No mean or/and standard deviation or/and number of subjects values |
| 2017 | Ghazy, A., Refaat, W. E., & Morcos, S. S. (2017). Three-Dimensional Evaluation of Cervical Vertebral Morphology in Skeletal Class II of Malocclusion in Egyptians. *Suez Canal University Medical Journal, 20*(1), 62-67. | No mean or/and standard deviation or/and number of subjects values |
| 2017 | Das, N. K., Chaudhry, N. A., Rahbar, M. I., & Riaz, A. (2017). Correlation between dental calcification stages and skeletal maturity indicators. *Pakistan Orthodontic Journal, 9*(2), 66-71. | No mean or/and standard deviation or/and number of subjects values |
| 2017 | Singla, R., Urala, A. S., Vineetha, R., & Singla, N. (2017). Skeletal maturity assessment using calcification stages of mandibular canine. *Journal of International Oral Health, 9*(3), 126-129. | No mean or/and standard deviation or/and number of subjects values |
| 2017 | Schlégl, Á. T., O'Sullivan, I., Varga, P., Than, P., & Vermes, C. (2017). Determination and correlation of lower limb anatomical parameters and bone age during skeletal growth (based on 1005 cases). Journal of orthopaedic research, 35(7), 1431–1441. https://doi.org/10.1002/jor.23390 | CVM stage not based on Baccetti’s method (2005) |
| 2017 | Kapoor, A. K., Thakur, S., Singhal, P., Chauhan, D., & Jayam, C. (2017). Compare, evaluate, and estimate chronological age with dental age and skeletal age in 6–14-year-old Himachali children. *International Journal of Health & Allied Sciences, 6*(3), 143-148. | No mean or/and standard deviation or/and number of subjects values |
| 2017 | Jain, P., Kaul, R., Mukhopadhyay, S., Saha, S., & Sarkar, S. (2017). Distance M-Me: A novel parameter having significant potential as a predictor of mandibular growth. *Indian journal of dental research, 28*(3), 320–324. https://doi.org/10.4103/ijdr.IJDR_87_17 | No mean or/and standard deviation or/and number of subjects values |
| 2017 | Sambataro, S., Fastuca, R., Oppermann, N. J., Lorusso, P., Baccetti, T., Franchi, L., & Caprioglio, A. (2017). Cephalometric changes in growing patients with increased vertical dimension treated with cervical headgear. *Journal of orofacial orthopedics , 78*(4), 312–320. https://doi.org/10.1007/s00056-017-0087-z | No mean or/and standard deviation or/and number of subjects values |
| 2017 | Jain, N., Tripathi, T., Gupta, S. K., Rai, P., Kanase, A., & Kalra, S. (2017). Serum IGF-1, IGFBP-3 and their ratio: Potential biochemical growth maturity indicators. *Progress in orthodontics, 18*(1), 1-8. https://doi.org/10.1186/s40510-017-0165-1 | No mean or/and standard deviation or/and number of subjects values |
| 2017 | Ferreira, P. E., Andrade, E. C., Drummond, A. F., França, E. C., Bastos, M. O., & Neves, L. S. (2017). Validação de um software para a estimação da idade óssea por meio das vértebras cervicais. *Arquivos em Odontologia, 53, 1-8*. | No mean or/and standard deviation or/and number of subjects values |
| 2017 | Semeunka, S. M., Fernandes, M. M., Prietch, J. R., Mundstock, K. S., & Fontanella, V. R. C. (2017). Estimativa Da Idade Com Finalidade Pericial Em Imagens Axiais Das Duas Primeiras Vértebras Cervicais–Estudo Piloto. *Revista Brasileira de Odontologia Legal, 4*(3), 13-23. | No mean or/and standard deviation or/and number of subjects values |
| 2017 | Yanagita, N., Terajima, M., Kanomi, R., & Takahashi, I. (2017). Three-dimensional analysis of pharyngeal airway morphology in Japanese female adolescents. *Orthodontic Waves, 76*(2), 89-96. | No mean or/and standard deviation or/and number of subjects values |
| 2017 | Torun G. S. (2017). Soft tissue changes in the orofacial region after rapid maxillary expansion : A cone beam computed tomography study. *Journal of orofacial orthopedics*, 78(3), 193–200. https://doi.org/10.1007/s00056-016-0074-9 | No mean or/and standard deviation or/and number of subjects values |
| 2017 | Mithun, K., Shamnur, N., Gopalkrishna B. R., Ashith M. V., Kumari, D., & Pereira, V. A. (2017). Evaluation of skeletal maturation using mandibular second molar calcification in south indian population. *Internacional Journal of Current research, 9*(7), 54632-54637. | CVM stage not based on Baccetti’s method (2005) |
| 2017 | Kocasarac, H. D., Altan, A. B., Yerlikaya, C., Sinanoglu, A., & Noujeim, M. (2017). Correlation between spheno-occipital synchondrosis, dental age, chronological age and cervical vertebrae maturation in Turkish population: is there a link?. Acta odontologica Scandinavica, 75(2), 79–86. https://doi.org/10.1080/00016357.2016.1255352 | CVM stage not based on Baccetti’s method (2005) |
| 2017 | Montasser, M. A., Viana, G., & Evans, C. A. (2017). Secular trends in the timing of skeletal maturation as assessed by the cervical vertebrae maturation method. *European journal of orthodontics, 39*(2), 188–193. https://doi.org/10.1093/ejo/cjw040 | CVM stage not based on Baccetti’s method (2005) |
| 2017 | Madhu, S. (2017). Correlation between Cervical Vertebrae Maturation and Chronological Age: A Radiographic Study. *World Journal of Dentistry, 8*(5), 382-385. | CVM stage not based on Baccetti’s method (2005) |
| 2017 | Khan, S., Thomas, M., Reddy, D., Eshky, R. T., & Fareed, W. M., (2017). Duration of the pubertal peak in skeletal class I, class II -div 1, div 2 and class III subjects - A cephalometric study. *Saudi Journal of Oral and Dental Research, 2*(2), 49-54. | CVM stage not based on Baccetti’s method (2005) |
| 2018 | Al-Balbeesi, H. O., Al-Nahas, N. W., Baidas, L. F., Bin Huraib, S. M., Alhaidari, R., & Alwadai, G. (2018). Correlation between skeletal maturation and developmental stages of canines and third molars among Saudi subjects. *The Saudi Dental Journal, 30*(1), 74–84. https://doi.org/https://doi.org/10.1016/j.sdentj.2017.11.003 | No mean or/and standard deviation or/and number of subjects values |
| 2018 | Imtiaz, H., Akbar, W., Jadoon, O. K., Ali, U., Ambreen, S., Javed, S., Shaheen, F., & Anwar, J. (2018). A Comparison Of Skeletal Age Of Thalassaemic Patients Of 9-15 Years With Chronological Age By Radiography. *Journal of Ayub Medical College, 30*(4), 642–646. | CVM stage not based on Baccetti’s method (2005) |
| 2018 | Kamal, A. T., Shaikh, A., & Fida, M. (2018). Assessment of skeletal maturity using the calcification stages of permanent mandibular teeth. Dental Press Journal of Orthodontics, 23(4), 44e1-8. https://dx.doi.org/10.1590/2177-6709.23.4.44.e1-8.onl | No mean or/and standard deviation or/and number of subjects values |
| 2018 | Kumagai, A., Willems, G., Franco, A., & Thevissen, P. (2018). Age estimation combining radiographic information of two dental and four skeletal predictors in children and subadults. *International Journal of Legal Medicine, 132*(6), 1769–1777. https://doi.org/10.1007/s00414-018-1910-9 | CVM stage not based on Baccetti’s method (2005) |
| 2018 | Görücü Coşkuner, H., Atik, E., & Taner, T. (2018). Relationship between midpalatal suture maturation and age and maturation of cervical vertebrae: radiographic evaluation. *Acta Odontologica Turcica, 35*(3), 69–74. https://doi.org/10.17214/gaziaot.406823 | No mean or/and standard deviation or/and number of subjects values |
| 2018 | Tehranchi, A., Younessian, F., Fadaei, V., Arabgol, F., & Shirvani, A. (2018). The Effect of Methylphenidate on Cervical Vertebral Maturation and Dental Age in Patients with Attention Deficit Hyperactivity Disorder. Journal of dentistry, 19(3), 197–205. | No mean or/and standard deviation or/and number of subjects values |
| 2018 | Mânica, S., Wong, F. S. L., Davis, G., & Liversidge, H. M. (2018). Estimating age using permanent molars and third cervical vertebrae shape with a novel semi-automated method. Journal of Forensic and Legal Medicine, 58, 140–144. https://doi.org/10.1016/j.jflm.2018.05.010 | CVM stage not based on Baccetti’s method (2005) |
| 2018 | Pamadya, S., Azhari, A., & Firman, R. N. (2018). Correlation of cervical vertebral maturity and teeth calcification stages in children with cleft lip and palate. Majalah Kedokteran Gigi Indonesia, 4(3), 167–171. https://doi.org/10.22146/majkedgiind.31875 | No mean or/and standard deviation or/and number of subjects values |
| 2018 | Chavanavesh, J., Petdachai, S., & Chuenchompoonut, V. (2018). The effects of sex, skeletal age, and sagittal skeletal pattern on pharyngeal airway dimensions and related structures in growing Thai orthodontic patients. Orthodontic Waves, 77(2), 1-14. | No mean or/and standard deviation or/and number of subjects values |
| 2018 | Perinetti, G., Primozic, J., Sharma, B., Cioffi, I., & Contardo, L. (2018). Cervical vertebral maturation method and mandibular growth peak: a longitudinal study of diagnostic reliability. *European journal of orthodontics, 40*(6), 666–672. https://doi.org/10.1093/ejo/cjy018 | No mean or/and standard deviation or/and number of subjects values |
| 2018 | Perinetti, G., Contardo, L., & Primozic, J. (2018). Diagnostic accuracy of the cervical vertebral maturation method. *European journal of orthodontics, 40*(4), 453–454. https://doi.org/10.1093/ejo/cjy043 | No mean or/and standard deviation or/and number of subjects values |
| 2018 | Srinivasan, B., Padmanabhan, S., & Chitharanjan, A. B. (2018). Constancy of cervical vertebral maturation indicator in adults: A cross-sectional study. *International orthodontics, 16*(3), 486–498. https://doi.org/10.1016/j.ortho.2018.06.015 | CVM stage not based on Baccetti’s method (2005) |
| 2018 | Batwa, W., Almoammar, K., Aljohar, A., Alhussein, A., Almujel, S., & Zawawi, K. H. (2018). The Difference in Cervical Vertebral Skeletal Maturation between Cleft Lip/Palate and Non-Cleft Lip/Palate Orthodontic Patients. BioMed research international, 2018. https://doi.org/10.1155/2018/5405376 | CVM stage not based on Baccetti’s method (2005) |
| 2018 | Dodmani, V. R., & Sunilkumar, Patil, C. G. B., Lavate, A. B., Hoshing, S. V., Singh, T. R., & Lipare, S. V. (2018). Relation between Frontal Sinus Morphology and the Cervical Vertebral Maturation for the Evaluation of the Skeletal Maturity among Solapur Population: A Cross Sectional Study. *Journal of Medical Science and Clinica Research, 6*(6), 432-437. | No mean or/and standard deviation or/and number of subjects values |
| 2018 | Cunha, A. C., Cevidanes, L. H., Sant'Anna, E. F., Guedes, F. R., Luiz, R. R., McNamara, J. A., Franchi, L., & Ruellas, A. (2018). Staging hand-wrist and cervical vertebrae images: a comparison of reproducibility. *Dento maxillo facial radiology, 47*(5). https://doi.org/10.1259/dmfr.20170301 | CVM stage not based on Baccetti’s method (2005) |
| 2018 | Lo Giudice, A., Caccianiga, G., Crimi, S., Cavallini, C., & Leonardi, R. (2018). Frequency and type of ponticulus posticus in a longitudinal sample of nonorthodontically treated patients: relationship with gender, age, skeletal maturity, and skeletal malocclusion. *Oral surgery, oral medicine, oral pathology and oral radiology, 126*(3), 291–297. https://doi.org/10.1016/j.oooo.2018.05.001 | CVM stage not based on Baccetti’s method (2005) |
| 2018 | Ayach, O. A., & Hadad, R. (2018). Correlation between Cervical Vertebrae Volume Parameter and the Skeletal Maturation Status. *The journal of contemporary dental practice, 19*(6), 662–668. | CVM stage not based on Baccetti’s method (2005) |
| 2018 | Cangialosi, T.J. and Vives, V.J. (2018) Another Look at Skeletal Maturation Using Hand Wrist and Cervical Vertebrae Evaluation. *Open Journal of Orthopedics, 8*, 1-10. https://doi.org/10.4236/ojo.2018.81001 | CVM stage not based on Baccetti’s method (2005) |
| 2018 | Samra, D. A., & Hadad, R. (2018). Skeletal Age-related Changes of Midpalatal Suture Densities in Skeletal Maxillary Constriction Patients: CBCT Study. *The journal of contemporary dental practice*, *19*(10), 1260–1266. | CVM stage not based on Baccetti’s method (2005) |
| 2018 | Tripathi, T., Gupta, P., Sharma, J., Rai, P., Gupta, V. K., & Singh, N. (2018). Bone-specific alkaline phosphatase - a potential biomarker for skeletal growth assessment. *Journal of orthodontics, 4*5(1), 4–10. https://doi.org/10.1080/14653125.2017.1416571 | CVM stage not based on Baccetti’s method (2005) |
| 2018 | Pryia, B., & Felicita, S. (2018). Evaluation of correlation between cervical vertebra, MP3 and canine calcification in Chennai population. *Drug Invention Today, 10*(1), 2614-2617. | CVM stage not based on Baccetti’s method (2005) |
| 2018 | Frank, D., Rill, L., Kolarovszki, B., & Nagy, Á. K. (2018). Classical and modern methods for the assessment of skeletal maturation and pubertal growth spurt. Orvosi hetilap, 159(35), 1423–1432. https://doi.org/10.1556/650.2018.31151 | CVM stage not based on Baccetti’s method (2005) |
| 2018 | Wani, B. A., Chalkoo, A. H., Tariq, S., Bedar, A. (2018). Assessment of bone age by cervical vertebral dimensions in lateral cephalometric radiographs. *Journal of Oral Medicine, Oral Surgery, Oral Pathology and Oral Radiology, 4*(3), 160-163. | CVM stage not based on Baccetti’s method (2005) |
| 2018 | Mahmood, H. T., Shaikh, A., & Fida, M. (2018). Reliability and validity of maxillary and sphenoid Sinus morphological variations in the assessment of Skeletal maturity. *Journal of Ayub Medical College,* *30*(3), 360–365. | CVM stage not based on Baccetti’s method (2005) |
| 2018 | Knapik, D. M., Abola, M. V., Gordon, Z. L., Seiler, J. G., Marcus, R. E., & Liu, R. W. (2018). Differences in Cross-Sectional Intervertebral Foraminal Area From C3 to C7. *Global spine journal*, *8*(6), 600–606. https://doi.org/10.1177/2192568218758085 | CVM stage not based on Baccetti’s method (2005) |
| 2018 | Stoilova-Todorova, M. G., Krasteva, S., Stoilov, G., & Todorova-Plachiyska. (2018). Comparison of skeletal maturity and chronological age in bulgarian female and male patients with transverse maxillary deficit. *Journal of IMAB, 24*(3), 2119-2124. | No mean or/and standard deviation or/and number of subjects values |
| 2018 | McNamara, J. A., & Franchi, L. (2018). The cervical vertebral maturation method: A user's guide. The Angle orthodontist, 88(2), 133–143. https://doi.org/10.2319/111517-787.1 | No mean or/and standard deviation or/and number of subjects values |
| 2018 | Raza, H. A., Ijaz, W., Ayub, A., & Rasool, G. (2018). Correlation between skeletal maturation and dental calcification stages. *Pakistan Orthodontic Journal, 10*(2), 98-105. | No mean or/and standard deviation or/and number of subjects values |
| 2018 | Vašková, S., Langová, K., & Černochová, P. (2018). Sinus frontalis a jeho vztah ke kostní zralosti. *Ortodoncie, 27*(4), 210-218. | No mean or/and standard deviation or/and number of subjects values |
| 2018 | Stoilova-Todorova, M. G., Krasteva, S., & Stoilov, G. (2018). Skeletal age assessment in patients with transverse maxillary deficit undergoing rapid maxillary expansion. *Journal of IMAB, 24*(3), 2113-2118. | No mean or/and standard deviation or/and number of subjects values |
| 2018 | Chang, H., Oh, M. H., Jung, C., & Cho, J. H. (2018). A longitudinal Study on Change of Mandibular Symphysis using metallic implants. *The journal of the Korean dental association, 56*(2), 94-102. | No mean or/and standard deviation or/and number of subjects values |
| **2018** | Irham, F., Bahirrah, S., & Nazruddin. (2018). The Level of Alkaline Phosphatase in Saliva as Biomarker for Pubertal Growth Phase. 102–105. https://doi.org/10.2991/idcsu-17.2018.27 | No mean or/and standard deviation or/and number of subjects values |
| 2018 | Singh, A., Tikku, T., Pratap, R., Verma, S. L., Khanna, R., Srivastava, K., & Srivastava, A. (2018). Correlation of Skeletal Maturity Indicators-(CVMI, MP3 & Frontal Sinus). *Heal Talk, 11*(2), 62-64. | CVM stage not based on Baccetti’s method (2005) |
| 2018 | Bodapati, S., Singaraju, G. S., Mandava, P., Chalasani, S., Nettam, V., & Unnam, D. (2018). Comparison of Body Mass Index Percentile with Two Different Types of Skeletal Maturity Indicators: An Observational Study. *Journal of Clinical & Diagnostic Research, 12*(3), 14-20. | No mean or/and standard deviation or/and number of subjects values |
| 2018 | Batwa, W., Almoammar, K., Aljohar, A., Alhussein, A., Almujel, S., & Zawawi, K. H. (2018). The Difference in Cervical Vertebral Skeletal Maturation between Cleft Lip/Palate and Non-Cleft Lip/Palate Orthodontic Patients. *BioMed Research International*, 2018, 1–5. 10.1155/2018/5405376 | No mean or/and standard deviation or/and number of subjects values |
| 2018 | Chavanavesh, J., Petdachai, S., & Chuenchompoonut, V. (2018). The effects of sex, skeletal age, and sagittal skeletal pattern on pharyngeal airway dimensions and related structures in growing Thai orthodontic patients. *Orthodontic Waves, 77*(2), 111-124. | No mean or/and standard deviation or/and number of subjects values |
| 2018 | León, R. D. P. R., Huallparimache, J. S., Zapata, C. S., Catacora, S. S., Díaz, G. F., Florián, C. T., & Victorio, D. B. (2018). Evaluación cuantitativa de la maduración ósea en vértebras cervicales con el uso de la tomografía computarizada Cone Beam. *Odontología sanmarquina, 21*(1), 27-33. | No mean or/and standard deviation or/and number of subjects values |
| 2018 | Tepedino, M., Iancu-Potrubacz, M., Ciavarella, D., Masedu, F., Marchione, L., & Chimenti, C. (2018). Expansion of permanent first molars with rapid maxillary expansion appliance anchored on primary second molars. *Journal of clinical and experimental dentistry, 10*(3), 241–247. https://doi.org/10.4317/jced.54585 | No mean or/and standard deviation or/and number of subjects values |
| 2018 | Golfeshan, F., Soltani, M. K., Zohrei, A., & Poorolajal, J. (2018). Comparison between Classic Twin-block and a Modified Clear Twin-block in Class II, Division 1 Malocclusions: A Randomized Clinical Trial. *The journal of contemporary dental practice, 19*(12), 1455-1462. | No mean or/and standard deviation or/and number of subjects values |
| 2018 | Ramírez-Velásquez, M., Viloria-Ávila, T. J., Rodríguez, D. A., Rojas, M. E., & Zambrano, O. (2018). Maturation of cervical vertebrae and chronological age in children and adolescents. Maduración de vértebras cervicales y edad cronológica en niños y adolescentes. *Acta odontologica latinoamericana, 31*(3), 125–130. | CVM stage not based on Baccetti’s method (2005) |
| 2018 | Torres, A., Rojas A., Torres, E.A., Rueda, S.J. & Rodríguez, M.J. (2018). Relationship between dental age, chronological age and cervical vertebral maturation in children and adolescents from Bucaramanga, Colombia. *Journal of Oral Research, 7*(5):190-197. 10.17126/joralres.2018.047 | CVM stage not based on Baccetti’s method (2005) |
| 2018 | Goyal S., Goyal, S., & Chopra, V. (2018). Comparative Evaluation of Development of Mandibular Second and Third Molars for the Assessment of Skeletal Maturity. *Journal of Contemporary Orthodontics, 2*(2), 29-38 | CVM stage not based on Baccetti’s method (2005) |
| 2018 | Ugurlu, M., & Ceylan, I. (2018). Assessment of cervical vertebra maturation in the determination of pubertal growth spurt phases.*The Journal of Dental Faculty of Atatürk University, 28*(4), 457-461. | CVM stage not based on Baccetti’s method (2005) |
| 2018 | Liu, X., & Chen, Z. (2018). Effects of Palate Repair on Cranial Base and Maxillary Morphology in Patients With Unilateral Complete Cleft Lip and Palate. The Cleft palate-craniofacial journal, 55(10), 1367–1374. https://doi.org/10.1177/1055665618768544 | CVM stage not based on Baccetti’s method (2005) |
| 2018 | Chamania, S., Patil, R. U., & Prakash, A. (2018). Evaluation of skeletal maturation in Indian adolescents using calcification stages of permanent mandibular second molar. *International Journal of Pedodontic Rehabilitation, 3*(2), 47. | CVM stage not based on Baccetti’s method (2005) |
| 2019 | Choudhary, S., Shankar, D., Sinha, S., Chandra, S., & Vuthoo, K. (2019). Evaluation of Accelerated Skeletal Maturation Period in Population of Bihar between 7 to 14 Years of Age Group. International Journal of Contemporary Medicine, 7(2), 11-15. | CVM stage not based on Baccetti’s method (2005) |
| 2019 | Fernandes-Retto, P., Matos, D., Ferreira, M., Bugaighis, I., & Delgado, A. (2019). Cervical vertebral maturation and its relationship to circum-pubertal phases of the dentition in a cohort of Portuguese individuals. Journal of clinical and experimental dentistry, 11(7), e642–e649. https://doi.org/10.4317/jced.55907 | No mean or/and standard deviation or/and number of subjects values |
| 2019 | Sheikh, S., Nene, S., J. Kalia, A., Gautam, R., Hegde, A., & P. Thakur, P. (2019). Relationship between age at menarche, body mass index percentile, and skeletal maturity stages in Indian female orthodontic patients. APOS Trends in Orthodontics, 9(1), 32–39. https://doi.org/10.25259/apos-9-1-6 | CVM stage not based on Baccetti’s method (2005) |
| 2019 | Alansari R. A. (2019). Diagnostic performance of eruption stages for identification of skeletal maturity. Saudi medical journal, 40(9), 954–957. https://doi.org/10.15537/smj.2019.9.23831 | No mean or/and standard deviation or/and number of subjects values |
| 2019 | Lévano, J. C. (2019). Relación de la edad cronológica con la maduración ósea cervical mediante el método de Baccetti. Revista Científica Odontológica, 7(2), 42–51. https://doi.org/10.21142/2523-2754-0702-2019-42-51 | No mean or/and standard deviation or/and number of subjects values |
| 2019 | Miller, C. A., Hwang, S. J., Cotter, M. M., & Vorperian, H. K. (2019). Cervical vertebral body growth and emergence of sexual dimorphism: a developmental study using computed tomography. Journal of Anatomy, 234(6), 764–777. https://doi.org/10.1111/joa.12976 | No mean or/and standard deviation or/and number of subjects values |
| 2019 | Vaida, L. L., Moca, A. E., Todor, L., Ţenţ, A., Todor, B. I., Negruţiu, B. M., & Moraru, A. I. (2019). Correlations between morphology of cervical vertebrae and dental eruption. Romanian journal of morphology and embryology, 60(1), 175–180. | No mean or/and standard deviation or/and number of subjects values |
| 2019 | Reverte-Salazar, M. G., Rosales-Berber, M. A., Pozos-Guillen, A. J., Garrocho-Ragel, J. A., Torre-Delgadillo, A., & Esparza-Villapando, V. (2019). Correlación entre la Edad Cronológica y Dental con los Estadios de Maduración Vertebral en Pacientes de 5 a 15 Años. Int. J. Morphol, 37(2), 548–553. | CVM stage not based on Baccetti’s method (2005) |
| 2019 | Shah, P. M., & Vignesh, R. (2019). Correlation of dental age, skeletal age, and chronological age among children aged 7-16 years: A retrospective study. Drug Invention Today, 11(1), 5–9. | No mean or/and standard deviation or/and number of subjects values |
| 2019 | Lee, Y., & Mah, Y. (2019). Evaluation of Midpalatal Suture Maturation using Cone-Beam Computed Tomography in Children and Adolescents. The Journal of the Korean Academy of Pedtatric Dentistry, 46(2), 139–146. https://doi.org/10.5933/jkapd.2019.46.2.139 | No mean or/and standard deviation or/and number of subjects values |
| 2019 | Patil, N., Maheshwari, N., Sharma, R., Soni, S., & Kushwah, A. (2019). Correlation between Chronological Age, Cervical Vertebral Maturation and Fishman’s Skeletal Maturity Indicators in Central India Population. Orthodontic Journal of Nepal, 9(2), 52–56. https://doi.org/10.3126/ojn.v9i2.28415 | CVM stage not based on Baccetti’s method (2005) |
| 2019 | Patel, A. J., & Shah, J. S. (2019). Age determination in children by orthopantomograph and lateral cephalogram: A comparative digital study. Journal of forensic dental sciences, 11(3), 118–124. https://doi.org/10.4103/jfo.jfds_61_19 | CVM stage not based on Baccetti’s method (2005) |
| 2019 | Jaiswal, A., Rohmetra, A., Ishita, Gupta, N., & Kulshrestha, R. (2018). Evaluation of relationship between chronological age cervical vertebrae maturation index method and canine calcification stages for the assessment of optimal treatment timing in orthodontic patients. International Journal of Oral Health Dentistry, 4(4), 214–221. https://doi.org/10.18231/2395-499X.2018.0049 | CVM stage not based on Baccetti’s method (2005) |
| 2019 | Kulkarni, N. (2019). Correlation of Cervical Vertebral Bone Age and Demirjian Stages of Dental Maturation for Lower Left Permanent Canine and Second Molar. The Journal of Contemporary Dental Practice, 20(4), 471–475. https://doi.org/10.5005/jp-journals-10024-2541 | CVM stage not based on Baccetti’s method (2005) |
| 2019 | Morris, K. M., Fields, H. W., Jr, Beck, F. M., & Kim, D. G. (2019). Diagnostic testing of cervical vertebral maturation staging: An independent assessment. *American journal of orthodontics and dentofacial orthopedics, 156*(5), 626–632. https://doi.org/10.1016/j.ajodo.2018.11.016 | No mean or/and standard deviation or/and number of subjects values |
| 2019 | Makaremi, M., Lacaule, C., & Mohammad-Djafari, A. (2019). Deep Learning and Artificial Intelligence for the Determination of the Cervical Vertebra Maturation Degree from Lateral Radiography. *Entropy, 21*(12), 1-24. | No mean or/and standard deviation or/and number of subjects values |
| 2019 | Uys, A., Bernitz, H., Pretorius, S., & Steyn, M. (2019). Age estimation from anterior cervical vertebral ring apophysis ossification in South Africans. *International journal of legal medicine, 133*(6), 1935–1948. https://doi.org/10.1007/s00414-019-02137-7 | No mean or/and standard deviation or/and number of subjects values |
| 2019 | Halithi, L. G., Toma, V., Pacurar, M., Georgescu, R., & Iliescu, C. (2019). Study on correlation between cervical vertebrae maturation, dental age and actual age of children. *The Medical-Surgical Journal, 123*(3), 531-537. | No mean or/and standard deviation or/and number of subjects values |
| 2019 | Desai, K., Thenmozhi, M. S., & Lakshmanan, G. (2019). Effect of cervical vertebrae 2, 3, and 4 on dental alignment. Drug Invention Today, 12(4), 758–760. | No mean or/and standard deviation or/and number of subjects values |
| 2019 | Jahanbin, A., Eslami, N., & Torkamanzadeh, N. (2020). Do Patients With Cleft Lip and Palate Differ From Normal Individuals in Skeletal Maturity?. The Journal of craniofacial surgery, 31(1), 186–188. https://doi.org/10.1097/SCS.0000000000005693 | CVM stage not based on Baccetti’s method (2005) |
| 2019 | Morris, K. M., Fields, H. W., Beck, F. M., & Kim, D. G. (2019). Diagnostic testing of cervical vertebral maturation staging: An independent assessment. *American Journal of Orthodontics and Dentofacial Orthopedics*, *156*(5), 626–632. doi:10.1016/j.ajodo.2018.11.016 | No mean or/and standard deviation or/and number of subjects values |
| 2019 | O'Sullivan, I., Schlégl, Á. T., Varga, P., Kerekes, K., Vermes, C., & Than, P. (2019). Bone age - alternatives for skeletal maturity assessment for the EOS scanner. Orvosi hetilap, 160(16), 619–628. https://doi.org/10.1556/650.2019.31337 | CVM stage not based on Baccetti’s method (2005) |
| 2019 | Montasser M. A. (2019). Craniofacial growth spurt in Class I subjects. American journal of orthodontics and dentofacial orthopedics, 155(4), 473–481. https://doi.org/10.1016/j.ajodo.2018.05.013 | No mean or/and standard deviation or/and number of subjects values |
| 2019 | Wijaya, H., Kusdhany, L. S., Redjeki, S., & Soegiharto, B. M. (2019). Salivary Bone-specific Alkaline Phosphatase as Predictor of Puberty Phase. *Journal of International Dental and Medical Research, 12*(3), 1063-1067. | CVM stage not based on Baccetti’s method (2005) |
| 2019 | Yi, S., Lee, D., Yang, Y., & Kim, J. (2019). Measuring Agreement of Modified MP3 and CVMS according to BMI Percentile. *The Journal of the Korean Academy of Pediatric Dentistry, 46*(1), 48-56. | No mean or/and standard deviation or/and number of subjects values |
| 2019 | Makhbul, M. Z. M., & Hassan, W. N. W. (2019). A clinical audit of the sucess rate of the removable funtional apliaccances treatment. *Malaysian Dental Journal, 1, 61-73*. | No mean or/and standard deviation or/and number of subjects values |
| 2019 | Alkan, Ö., Aydoğan, C., & Akkaya, S. (2016). Morphological comparison of cervical vertebrae in adult females with different sagittal craniofacial patterns: A cross-sectional study. Journal of craniovertebral junction & spine, 7(3), 135–139. https://doi.org/10.4103/0974-8237.188409 | CVM stage not based on Baccetti’s method (2005) |
| 2019 | Nancy, E. D., Yezdani, A. A., Kannan, M. S., Kumar, S. K., & Padmavathy, K. (2019). Serum Insulin Like Growth Factor-1–A Skeletal Maturity Indicator for the Assessment of Orthopedic Treatment Timing of Skeletal Class II Malocclusion. *Biomedical and Pharmacology Journal, 12*(1), 233-238. | No mean or/and standard deviation or/and number of subjects values |
| 2019 | Sulaiman, S. P., & Chatra, L. (2019). The Efficacy of Demirjian’s Method and Mito ET AL Method in Age Estimation–A Comparative Study. *Journal of Dental and Medical Sciences, 18*(7), 41-45. | CVM stage not based on Baccetti’s method (2005) |
| 2019 | Komala, W., Mardiati, E., Soemantri, E. S., & Malik, I. (2019). Physiological maturation stage of cervical vertebrate index in cleft lip/palate and non-cleft lip/palate patients. *Majalah Kedokteran Gigi Indonesia, 4*(3), 149-153. | CVM stage not based on Baccetti’s method (2005) |
| 2019 | Akarsu-Guven, B., Karakaya, J., Ozgur, F., & Aksu, M. (2019). Upper airway features of unilateral cleft lip and palate patients in different growth stages. *The Angle orthodontist, 89*(4), 575–582. https://doi.org/10.2319/022518-155.1 | No mean or/and standard deviation or/and number of subjects values |
| 2019 | Dhoka, S. R., Deshmukh, S., Jethe, S., Rahalkar, J. S., & Bhattacharya, S. (2019). Reliability of Biological Marker, Insulin Like Growth Factor-1 (IGF-1) as an Indicator in Assessing Skeletal Maturity Using Blood Sample By ELISA Technique. J*ournal of Advanced Medical and Dental Sciences Research, 7*(1), 104-112. | No mean or/and standard deviation or/and number of subjects values |
| 2019 | Oh, E., Ahn, S. J., & Sonnesen, L. (2019). Ethnic differences in craniofacial and upper spine morphology between European and Asian children with skeletal Class III malocclusion. *American journal of orthodontics and dentofacial orthopedics, 156*(4), 502–511. https://doi.org/10.1016/j.ajodo.2018.10.024 | No mean or/and standard deviation or/and number of subjects values |
| 2019 | Shafir, N. B. S. (2019). Secular Trends in Age at Onset of Orthodontic Treatment in Adolescents. *Journal of Dental Science Research Review, 1*(1), 2-5. | No mean or/and standard deviation or/and number of subjects values |
| 2019 | Lee, Y. S., Choi, S. H., Kim, K. H., & Hwang, C. J. (2019). Evaluation of skeletal maturity in the cervical vertebrae and hand-wrist in relation to vertical facial types. *Korean journal of orthodontics, 49*(5), 319–325. https://doi.org/10.4041/kjod.2019.49.5.319 | No mean or/and standard deviation or/and number of subjects values |
| 2019 | Kharbanda, O. P., Qureshi, T., & Kandasamy, D. (2019). Prevalence of cervical vertebrae anomalies in patients with cleft lip and palate. *Australasian Orthodontic Journal, 35*(1), 46–52. https://doi.org/10.3316/informit.489549758683275 | No mean or/and standard deviation or/and number of subjects values |
| 2019 | Yildrim, D., Amasya, H., Aydoğan, T., & Kemaloğlu, N. (2019). Lateral sefalometrik görüntülerde servikal vertebra morfolojisinin görsel ve yazılım destekli analizinde gözlemci uyumu. *Selcuk Dental Journal, 6*(4), 382-387. | No mean or/and standard deviation or/and number of subjects values |
| 2019 | Yoo, H., Ra, J., & Lee, J. (2019). Skeletal Maturity Evaluation using Maxillary Canine Development in Growing Children. *The Journal of the Korean Academy of Pediatric Dentistry, 46*(3), 247-254. | CVM stage not based on Baccetti’s method (2005) |
| 2019 | Kök, H., Acilar, A. M., & İzgi, M. S. (2019). Usage and comparison of artificial intelligence algorithms for determination of growth and development by cervical vertebrae stages in orthodontics. Progress in orthodontics, 20(41), 1-10. https://doi.org/10.1186/s40510-019-0295-8 | CVM stage not based on Baccetti’s method (2005) |
| 2019 | Khan, K., Amarnath, B. C., Prashanth , C. S., Roopak , M. D., & Karupakala, P. (2019). A comparison of salivary Indian hedgehog (IHH) protein levels and cervical maturational stages as growth indicators. *International Journal of Applied Dental Sciences, 5*(3), 151-162. | CVM stage not based on Baccetti’s method (2005) |
| 2019 | Dhanare, P., Panda, S., Bhardwaj, S., & Sharma, A. (2019). An evaluation of skeletal age using lateral cephalogram for cervical vertebrae and intraoral periapical x-ray for MP3: a radiographic study. *International Journal of Scientifc Research,* *8*(4), 58-61. | CVM stage not based on Baccetti’s method (2005) |
| 2019 | Alhazmi, N., Trotman, C. A., Finkelman, M., Hawley, D., Zoukhri, D., & Papathanasiou, E. (2019). Salivary alkaline phosphatase activity and chronological age as indicators for skeletal maturity. *The Angle orthodontist, 89*(4), 637–642. https://doi.org/10.2319/030918-197.1 | CVM stage not based on Baccetti’s method (2005) |
| 2019 | Tripathi, T., Gupta, P., Rai, P., Sharma, J., Gupta, V. K., Singh, N., & Verma, M. (2019). Longitudinal evaluation of the association between Insulin-like growth factor-1, Bone specific alkaline phosphatase and changes in mandibular length. Scientific reports, 9(1), 1-9. https://doi.org/10.1038/s41598-019-48067-7 | CVM stage not based on Baccetti’s method (2005) |
| 2019 | Seyedashrafi, M. M., Payahoo, S., Noorizade, A., & Noruzi, M. (2019). Relationship of Morphological Changes of the First Molar Pulp Chamber and Mineralization of Developing Third Molar with Cervical Vertebral Maturation on Panoramic Radiographs and Lateral Cephalograms. *International Journal of Clinical Skills, 13*(2), 278-287. | CVM stage not based on Baccetti’s method (2005) |
| 2020 | Gulsahi, A., Çehreli, S. B., Galić, I., Ferrante, L., & Cameriere, R. (2020). Age estimation in Turkish children and young adolescents using fourth cervical vertebra. International journal of legal medicine, 134(5), 1823–1829. https://doi.org/10.1007/s00414-020-02246-8 | CVM stage not based on Baccetti’s method (2005) |
| 2020 | Goncharuk-Khomyn, M., Akleyin, E., Zhulkevych, I., Nahirnyi, Y., Brekhlichuk, P., Mochalov, Y., Melnychuk, I., Horzov, L., & Stoika, O. (2020). Correspondence between dental and skeletal maturity parameters among patients with different sagittal relationships at the end of puberty period. *Journal of International Dental and Medical Research, 13*(1), 223–228. | No mean or/and standard deviation or/and number of subjects values |
| 2020 | Zhang, Y., Shu, S., Gu, Q., Liu, Z., Zhu, Z., Qiu, Y., & Bao, H. (2020). Cervical vertebral maturation (CVM) stage as a supplementary indicator for the assessment of peak height velocity (PHV) in adolescent idiopathic scoliosis (AIS). Quantitative Imaging in Medicine and Surgery, 10(1), 96–105. https://doi.org/10.21037/qims.2019.11.07 | No mean or/and standard deviation or/and number of subjects values |
| 2020 | Fang, X., Fan, C., Jiang, C., Xue, Q., Xiao, W., Tao, X., Tian, Z., & Xu, X. (2020). Relationship between dental calcification of mandibular teeth and cervical vertebrae maturity in patients with unilateral complete cleft lip and palate. *Oral radiology*, 37, 209-217. https://doi.org/10.1007/s11282-020-00433-2 | No mean or/and standard deviation or/and number of subjects values |
| 2020 | Priyanka, J. N., Chaitanya, N. C. S. K., Srivani, G. S., Mounika, Y., Reddy, G. R., Priya, B., Sanjna, C. R., & Sahiti, R. (2020). Correlating the Age Estimated by Nolla’s Method and Modified Demirjian Method with Cervical Vertebral Maturation Index: A Cross-Sectional Study. Journal of the International Clinical Dental Research Organization, 12(2), 132-139. https://doi.org/10.4103/jicdro.jicdro_13_20 | CVM stage not based on Baccetti’s method (2005) |
| 2020 | Franchi, L., Nieri, M., McNamara, J. A., Jr, & Giuntini, V. (2021). Predicting mandibular growth based on CVM stage and gender and with chronological age as a curvilinear variable. *Orthodontics & craniofacial research*, 24(3), 414–420. https://doi.org/10.1111/ocr.12457 | No mean or/and standard deviation or/and number of subjects values |
| 2020 | Mohammad R. J. (2020). Correlation between skeletal development and maxillary canine eruption. Indian journal of dental research : official publication of Indian Society for Dental Research, 31(3), 408–413. https://doi.org/10.4103/ijdr.IJDR_29_19 | CVM stage not based on Baccetti’s method (2005) |
| 2020 | Zaghloul, M., Al-Dany, A., Farouk, K. (2020). Reliability of Mandibular Canine Maturation Stages in Predicting Pubertal Growth Spurt in a Sample of Egyptian Orthodontic Patients. *Al-Azhar Journal of Dental Science, 23*(2), 139-146. https://doi.org/10.21608/ajdsm.2020.24556.1001 | CVM stage not based on Baccetti’s method (2005) |
| 2020 | Korde, S., Daigavane, P., Khakhar, P. G., Niranjane, P., & Chimote, B. (2020). Association of Skeletal and Dental Maturity Indicators with the Onset of Menarche and Its Applicability for Growth Modification of Jaw Bases in Females Aged between 7 and 14 Years. Journal of Evolution of Medical and Dental Sciences, 9(18), 1484-1489. | No mean or/and standard deviation or/and number of subjects values |
| 2020 | Sousa , R. P. R., Clemente, S. M. P. S., Lima, A. T., Diniz, C. N., Cavalcante, L. H. A., Sousa, J. P., Massoni , A. C .L. T., Bento, P. M,, Freitas, A. P. L. F., & Diniz, D. N. (2020). Reproducibility assessment among three methods for determining skeletal maturation in patients from 06 to 16 years. Research, Society and Development, 9(12), 1-15. https://doi.org/10.33448/rsd-v9i12.11046 | CVM stage not based on Baccetti’s method (2005) |
| 2020 | Carelli, J., Madalena, I. R., Morais, N. D., & Moro, A. (2020). Correlation between skeletal and dental maturity methods in Brazilian children. Revista Sul Brasileira de Odontologia, 17(2), 162–171. | No mean or/and standard deviation or/and number of subjects values |
| 2020 | Cahuana, L. J. G. (2020). Correlación del IMC con la maduración ósea de vértebras cervicales y edad dental en niños y adolescentes. Revista Odontológica Basadrina, 4(1), 10–15. https://doi.org/10.33326/26644649.2020.4.1.909 | CVM stage not based on Baccetti’s method (2005) |
| 2020 | Chandrasekar, R., Chandrasekhar, S., Sundari, K. K. S., & Ravi, P. (2020). Development and validation of a formula for objective assessment of cervical vertebral bone age. Progress in Orthodontics, 21(38), 1-8. https://doi.org/10.1186/s40510-020-00338-0 | No mean or/and standard deviation or/and number of subjects values |
| 2020 | Hasan, S. M., Abbas, H. H., & Hasan, N. M. (2020). Estimation of dental and skeletal age in iron-deficient anemic patients of Iraq. Journal of Cardiovascular Disease Research, 11(4), 218–224. https://doi.org/10.31838/jcdr.2020.11.04.39 | CVM stage not based on Baccetti’s method (2005) |
| 2020 | Kasimoglu, Y., Marsan, G., & Gencay, K. (2020). Skeletal Maturity Prediction Using Radiographs of the Medial Phalanx of the Third Finger and Cervical Vertebrae. Int J Med Invest, 9(1), 42–49. | No mean or/and standard deviation or/and number of subjects values |
| 2020 | Toodehzaeim, M. H., Rafiei, E., Hosseini, S. H., Haerian, A., & Hazeri-Baqdad-Abad, M. (2020). Association between mandibular second molars calcification stages in the panoramic images and cervical vertebral maturity in the lateral cephalometric images. Journal of clinical and experimental dentistry, 12(2), 148–153. https://doi.org/10.4317/jced.56402 | No mean or/and standard deviation or/and number of subjects values |
| 2020 | Fayad, R., Kassis, A., Akl, R., Ghoubril, J., & Khoury, E. (2020). Correlation between fusion of spheno-occipital synchondrosis and cervical vertebral maturation: A CBCT and cephalometric assessment. International Orthodontics, 18(4), 749–757. https://doi.org/10.1016/j.ortho.2020.09.003 | No mean or/and standard deviation or/and number of subjects values |
| 2020 | Sadiq, M. N., Shamim, A., Azeem, M., Hussain, S., Ul Haq, A., Murtaza, N., & Ul Hamid, W. (2020). Correlation Between Serum IGF-1 Levels and CVM Stages for the Assessment of Skeletal Maturity. Journal of the Pakistan Dental Association, 29(01), 09–13. https://doi.org/10.25301/JPDA.291.9 | CVM stage not based on Baccetti’s method (2005) |
| 2020 | Khajah, A., Tadinada, A., Allareddy, V., Kuo, C.-L., Nanda, R., & Uribe, F. (2020). Influence of type of radiograph and levels of experience and training on reproducibility of the cervical vertebral maturation method. American Journal of Orthodontics and Dentofacial Orthopedics, 157(2), 228–239. doi:10.1016/j.ajodo.2019.03.025 | No mean or/and standard deviation or/and number of subjects values |
| 2020 | Amasya, H., Cesur, E., Yidrim, D., & Orhan, K. (2020). Validation of cervical vertebral maturation stages: Artificial intelligence vs human observer visual analysis. American Journal of Orthodontics and Dentofacial Orthopedics, 158(6), 173-179. | No mean or/and standard deviation or/and number of subjects values |
| 2020 | [Danze, A., Jacox, L. A., Bocklage, C., Whitley, J., Moss, K., Hardigan, P., Garcia-Godoy, C. E., & Jackson, T. H. (2021). Influence of BMI percentile on craniofacial morphology and development in children and adolescents. European journal of orthodontics, 43(2), 184–192. https://doi.org/10.1093/ejo/cjaa056](https://doi.org/10.1093/ejo/cjaa056) | CVM stage not based on Baccetti’s method (2005) |
| 2020 | Perinetti, G., Braga, C., Contardo, L., & Primozic, J. (2020). Cervical vertebral maturation: Are postpubertal stages attained in all subjects? *American journal of orthodontics and dentofacial orthopedics, 157*(3), 305–312. https://doi.org/10.1016/j.ajodo.2019.03.026 | No mean or/and standard deviation or/and number of subjects values |
| 2020 | Kök, H., Izgi, M. S., & Acilar, A. M. (2020). Determination of growth and development periods in orthodontics with artificial neural network. Orthodontics & craniofacial research, 1.8. https://doi.org/10.1111/ocr.12443 | No mean or/and standard deviation or/and number of subjects values |
| 2020 | Gladea, Z., Budiardjo, S. B., & Rizal, M. F. (2020). Mapping Cervical Vertebral Maturation Levels with the Dimensions of Frontal Sinuses in a Sample of Indonesian Children. *Journal of International Dental and Medical Research, 13*(3), 1165-1169. | No mean or/and standard deviation or/and number of subjects values |
| 2020 | Alijani, S., Farhadian, N., Alafchi, B., & Najafi, M. (2020). Relationship of Frontal Sinus Size and Maturation of Cervical Vertebrae for Assessment of Skeletal Maturity. *Frontiers in dentistry, 17*(20), 1–6. | No mean or/and standard deviation or/and number of subjects values |
| 2020 | Basheer, S., Thimmaiah, S., & Alle, R. S. (2020). Assessment of Cervical Vertebral Bone Mineral Density in Adolescents Undergoing Functional Appliance Treatment. *The journal of contemporary dental practice*, *21*(7), 756–759. | CVM stage not based on Baccetti’s method (2005) |
| 2020 | [Akan, B., & Veli, İ. (2020). Evaluation of soft-tissue changes in young adults treated with the Forsus fatigue-resistant device. American journal of orthodontics and dentofacial orthopedics, 157(4), 481–489.e2. https://doi.org/10.1016/j.ajodo.2019.05.014](https://doi.org/10.1016/j.ajodo.2019.05.014) | CVM stage not based on Baccetti’s method (2005) |
| 2020 | Yezdani, A., Sreenivasan, P., Gnanashanmugham, K., Kannan, M. S., Kumar, K., & Padmavathy, K. (2020). Morphometric and cephalometric assessment of cervical vertebral maturation stages and its implications in dentofacialorthopedics. *Journal of Critical Reviews, 7*(14), 3684-3691. | No mean or/and standard deviation or/and number of subjects values |
| 2020 | Andreas, P. .R., Budiardjo, S. & Suharsini, M. (2020). The correlation between cervical vertebral maturation and mandibular dimensions in children aged 8-16 years: study on children's population in Jakarta, Indonesia. *Journal of Stomatology, 73*(6), 308-312. | No mean or/and standard deviation or/and number of subjects values |
| 2020 | Echevarría-Sánchez, G., Arriola-Guillén, L. E., Malpartida-Carrillo, V., Tinedo-López, P. L., Palti-Menendez, R., & Guerrero, M. E. (2020). Reliability of cephalograms derived of cone beam computed tomography versus lateral cephalograms to estimate cervical vertebrae maturity in a Peruvian population: A retrospective study. *International orthodontics, 18*(2), 258–265. https://doi.org/10.1016/j.ortho.2020.01.001 | No mean or/and standard deviation or/and number of subjects values |
| 2020 | Bozkurt, A. P., Aras, I., Othman, E., & Aras, A. (2020). Comparison of 2 treatment protocols using fixed functional appliances in Class II malocclusion: Treatment results and stability. *American Journal of Orthodontics and Dentofacial Orthopedics*, *157*(4), 474–480. doi:10.1016/j.ajodo.2019.05.013 | No mean or/and standard deviation or/and number of subjects values |
| 2020 | Dogan, E., & Seckin, O. (2020). Maxillary protraction in patients with unilateral cleft lip and palate : Evaluation of soft and hard tissues using the Alt-RAMEC protocol. Maxilläre Protraktion bei Patienten mit einseitiger Lippen-Kiefer-Gaumenspalte : Evaluierung von Weich- und Hartgeweben mit dem Alt-RAMEC-Protokoll. *Journal of orofacial orthopedics, 8*1(3), 209–219. https://doi.org/10.1007/s00056-020-00220-y | No mean or/and standard deviation or/and number of subjects values |
| 2020 | Amasya, H., Yildirim, D., Aydogan, T., Kemaloglu, N., & Orhan, K. (2020). Cervical vertebral maturation assessment on lateral cephalometric radiographs using artificial intelligence: comparison of machine learning classifier models. *Dento maxillo facial radiology*, *49*(5). https://doi.org/10.1259/dmfr.20190441 | No mean or/and standard deviation or/and number of subjects values |
| 2020 | Perinetti, G., Braga, C., Contardo, L., & Primozic, J. (2020). Cervical vertebral maturation: Are postpubertal stages attained in all subjects? *American Journal of Orthodontics and Dentofacial Orthopedics*, *157*(3), 305–312. doi:10.1016/j.ajodo.2019.03.026 | No mean or/and standard deviation or/and number of subjects values |
| 2020 | James, J., Sundareswaran, S., & Davis, S. (2020). Effect of adding daytime Class III Elastics to the alternate rapid maxillary expansion-constriction and reverse headgear therapy - A randomized clinical trial. Journal of orthodontic science, 9, 13. https://doi.org/10.4103/jos.JOS_71_19 | CVM stage not based on Baccetti’s method (2005) |
| 2020 | Kang, S. T., Choi, S. H., Kim, K. H., & Hwang, C. J. (2020). Evaluation of cephalometric characteristics and skeletal maturation of the cervical vertebrae and hand-wrist in girls with central precocious puberty. *Korean journal of orthodontics, 5*0(3), 181–187. https://doi.org/10.4041/kjod.2020.50.3.181 | CVM stage not based on Baccetti’s method (2005) |
| 2020 | Adisen, S. R., Adisen, M. Z., & Ozdiler, F. E. (2020). The evaluation of the relationship between cervical vertebral anomalies with skeletal malocclusion types and upper airway dimensions. The Journal of Craniomandibular & Sleep Practice, 38(3), 149–157. https://doi.org/10.1080/08869634.2018.1503136 | CVM stage not based on Baccetti’s method (2005) |
| 2020 | Sah, S., Bhattacharya, P., Bhandari, R., Anwer, T., Joshi, S., Singh, A. P., & Muzaffar, A. (2020). Adolescent Body Mass Index and Skeletal Maturation Assessed with Hassel and Farman’s Cervical Vertebrae Staging Method. *Journal of Dental Sciences and Oral Rehabilitation, 11*(1), 7-10. | CVM stage not based on Baccetti’s method (2005) |
| 2020 | Mahdian, A., Safi, Y., Dalaie, K., Kavousinejad, S., & Behnaz, M. (2020). Correlation assessment of cervical vertebrae maturation stage and mid-palatal suture maturation in an Iranian population. *Journal of the World federation of orthodontists, 9*(3), 112–116. https://doi.org/10.1016/j.ejwf.2020.05.004 | No mean or/and standard deviation or/and number of subjects values |
| 2020 | Chutasripanich, N., Mahatumarat, K., & Panmekiate, S. (2020). Relationship between the Midpalatal Suture Maturation, Cervical Vertebral Maturation and Dental Age in 8-18 Years Old Patients | No mean or/and standard deviation or/and number of subjects values |
| 2020 | Utama, V., Soedarsono, N., & Yuniastuti, M. (2020). Assessment of agreement between cervical vertebrae skeletal and dental age estimation with chronological age in an Indonesian population. *Journal of Forensic Odonto-Stomatology, 38*(3), 16-24. | No mean or/and standard deviation or/and number of subjects values |
| 2020 | Emanuel, M. A., Tabita, M. R., Radu, S., Ligia, V. L., Anamaria, Ț., Liana, T., & Maria, N. B. (2020). Chronological age, dental age and skeletal age in orthodontic patients. *Research and Clinical Medicine, 4*(3), 30-35. | No mean or/and standard deviation or/and number of subjects values |
| 2020 | Hostage, M., Silver, M. T., Finn, S. C., Canary, B., Kantarci, A., Allareddy, V., Katebi, N., & Masoud, M. I. (2020). Developmental stage specific ANB reference values based on a longitudinal sample of untreated Caucasian subjects. *Australasian Orthodontic Journal, 36*(1), 69-74. | No mean or/and standard deviation or/and number of subjects values |
| 2020 | Anusuya, V., Sharan, J., & Jena, A. K. (2020). A study of cervical vertebra anomalies among individuals with different sagittal and vertical facial growth patterns. *Journal of craniovertebral junction & spine, 11*(2), 75–80. https://doi.org/10.4103/jcvjs.JCVJS_51_20 | No mean or/and standard deviation or/and number of subjects values |
| 2020 | Dargahwala, H. S., Daigavane, P., SD, V., Kamble, R., Shrivastav, S., & Khakhar, P. G. (2020). Comparison of Cervical Vertebral Body Volume in Class II Vertical and Class II Horizontal Cases With Class I Cases Using 3D-DVT. *Journal of Indian Orthodontic Society, 5*4(4), 332-337. | No mean or/and standard deviation or/and number of subjects values |
| 2020 | Thomas, A. A., Subramanian, A. K., & Varghese, R. M. (2020). Assessing The Relationship Between The Cervical Vertebrae Maturation Stages And Permanent Maxillary Canine Calcification Stages-A Retrospective Study. *International Journal of Pharmaceutical Research, 12*(4), 2116-2123. | CVM stage not based on Baccetti’s method (2005) |
| 2020 | Sihombing, T. R., & Lubis, M. M. (2020). Relationship between cervical vertebrae maturity and mandibular length. *Jurnal Kedokteran Gigi Universitas Padjadjaran, 32*(3), 205-211. | No mean or/and standard deviation or/and number of subjects values |
| 2020 | Celebi, F., & Akbulut, S. (2020). Relationship between the position of maxilla and rapid maxillary expansion failure. *South European Journal of Orthodontics and Dentofacial Research, 7*(2), 49-54. | No mean or/and standard deviation or/and number of subjects values |
| 2020 | Hosoyama, C., Hosoyama, Y., Azumi, E., Nishiura, A., Mori, A., & Matsumoto, N. (2020). Bone growth assessed by cephalometric radiographs correlates with impacted canine diagnosis. J*ournal of Osaka Dental University, 54*(2), 283-292. | No mean or/and standard deviation or/and number of subjects values |
| 2020 | Logamarta, S. W., Romdlon, M. A., & Anggraeni, Y. (2020). Perbedaan Dimensi Anteroposterior Wajah Anak Laki-Laki Dan Perempuan Berdasarkan Tingkat Maturasi Tulang Vertebra Servikalis Pada Periode Gigi Bercampur. *Prosiding, 9(*1), 66-74. | No mean or/and standard deviation or/and number of subjects values |
| 2020 | Carelli, J., Madalena, I. R., Mattos, C., Morais, N. D., de França Lopes, C. M. C., Scariot, R., Brancher, J. A., Kuchler, E., & Moro, A. (2020). Avaliação da correlação entre maturação esquelética e maturação dentária em crianças brasileiras. *Revista Sul Brasileira de Odontologia, 17*(2), 162-171. | No mean or/and standard deviation or/and number of subjects values |
| 2020 | Mahendra, P., Pradopo, S., & Puteri, M. M. (2020). Pubertal Growth Spurt Peak in Angle Class I and II Malocclusions Using Cervical Vertebrae Maturation Analysis in Deutero-Malay Children. *Acta Medica Philippina, 1-5*. | No mean or/and standard deviation or/and number of subjects values |
| 2020 | Tekın, A., & Aydın, K. C. (2020). Comparative determination of skeletal maturity by hand–wrist radiograph, cephalometric radiograph and cone beam computed tomography. *Oral radiology*, *36*(4), 327-336. | CVM stage not based on Baccetti’s method (2005) |
| 2020 | Erhamza, T. S., Kilicaslan, Y., & Unver, F. N. (2020). Effect of body mass index percentile on skeletal maturation of cervical vertebrae and hand-wrist and dental maturation. *Acta odontologica Scandinavica, 78*(3), 236–240. https://doi.org/10.1080/00016357.2019.1709891 | CVM stage not based on Baccetti’s method (2005) |
| 2020 | Oyonarte, R., Sánchez-Ugarte, F., Montt, J., Cisternas, A., Morales-Huber, R., Ramirez-Lobos, V., & Janson, G. (2020). Diagnostic assessment of tooth maturation of the mandibular second molars as a skeletal maturation indicator: A retrospective longitudinal study. American journal of orthodontics and dentofacial orthopedics, 158(3), 383–390. https://doi.org/10.1016/j.ajodo.2019.09.012 | CVM stage not based on Baccetti’s method (2005) |
| 2020 | Purevjav, E., Radnaadorj, A., Ganburged, G., Mashbat, B., Bazar, A., Moriyama, K., & Gombojav, B. (2020). Facial Soft Tissue Profile Analysis in Mongolian Children. *Central Asian Journal of Medical Sciences, 6*(4), 214-222. | CVM stage not based on Baccetti’s method (2005) |
| 2021 | Koçak, T., & Akan, B. (2021). Assessment of maturation indicators in individuals with different skeletal malocclusion. Bewertung von Reifungsindikatoren bei unterschiedlichen skelettalen Malokklusionen. Journal of orofacial orthopedics , 82(3), 187–197. https://doi.org/10.1007/s00056-021-00286-2 | CVM stage not based on Baccetti’s method (2005) |
| 2021 | Lin, J., Lu, S., Feng, X., & Li, Y. (2021). Establishment of an intelligent cervical vertebrae maturity assessment system based on cone beam CT data. Journal of Zhejiang University, 50(2), 187–194. https://doi.org/10.3724/zdxbyxb-2021-0131 | No mean or/and standard deviation or/and number of subjects values |
| 2021 | Jesus, A. S., Oliveira, C. B., Murata, W. H., Suzuki, S. S., & Santos-Pinto, A. D. (2021). Would midpalatal suture characteristics help to predict the success rate of miniscrew-assisted rapid palatal expansion?. *American journal of orthodontics and dentofacial orthopedics,* (21). | CVM stage not based on Baccetti’s method (2005) |
| 2021 | Franchi, L., Nieri, M., Lomonaco, I., McNamara, J. A., & Giuntini, V. (2021). Predicting the mandibular growth spurt. *The Angle orthodontist*, 91(3), 307–312. https://doi.org/10.2319/080220-676.1 | No mean or/and standard deviation or/and number of subjects values |
| 2021 | Thierens, L., Manalili, L., De Roo, N., Verdonck, A., De Llano-Pérula, M. C., & De Pauw, G. (2021). Assessment of craniofacial maturation in preadolescents with cleft lip and/or palate using the cervical vertebral maturation method. Clinical oral investigations, 25(8), 4851–4859. https://doi.org/10.1007/s00784-021-03790-2 | No mean or/and standard deviation or/and number of subjects values |
| 2021 | Verma, S. L., Tikku, T., Khanna, R., Maurya, R. P., Srivastava, K., & Singh, V. (2021). Predictive accuracy of estimating mandibular growth potential by regression equation using cervical vertebral bone age. National journal of maxillofacial surgery, 12(1), 25–35. https://doi.org/10.4103/njms.NJMS_264_20 | CVM stage not based on Baccetti’s method (2005) |
| 2021 | Carelli, J., Mattos, C., Morais, N. D., Scariot, R., Brancher, J. A., Baratto-Filho, F., Kuchler, E. C., & Moro, A. (2021). Correlation between Insulin-Like Growth Factor I and Skeletal Maturity Indicators. *Global pediatric health, 8, 1-6.* https://doi.org/10.1177/2333794X211011305 | No mean or/and standard deviation or/and number of subjects values |
| 2021 | Chu, Y., Zhang, L., Zhao, Y., Yi, F., & Lu, Y. (2021). Effectiveness of modifications to preadjusted appliance prescriptions based on racial dental characteristics assessed by the ABO Cast-Radiograph Evaluation: A propensity score matching study. PeerJ, 9, e10605. https://doi.org/10.7717/peerj.10605 | No mean or/and standard deviation or/and number of subjects values |
| 2021 | Muthe, K., Kumar, S., Achutha, G., Surana, P., Ankush, B., & Bharadwaj, S. R. (2021). Assessment of Growth Using Mandibular Canine Calcification Stages andIts Correlation with Modified MP3 Stages and Cervica lVertebrae Maturation Stages. *Annals of the Romanian Society for Cell Biology, 25*(6), 8464-8478. | CVM stage not based on Baccetti’s method (2005) |
| 2021 | Souza, D. C. C., Nóbrega, M. T. C., Pires, A. C., & Lacerda, R. H. W. (2021). Correlation between different methods of assessing bone maturation. *Revista de Ciências Médicas e Biológicas, 20*(1), 75-82. | CVM stage not based on Baccetti’s method (2005) |
| 2021 | Andrade, J. M., & Tinitana, V., V. (2021). Correlation of the Baccetti method of skeletal maturation with chronological age on lateral skull radiographs in Cuenca-Ecuador. *Revista Cientifica "Especialiades Odontológicas UG", 4*(1). | No mean or/and standard deviation or/and number of subjects values |
| 2021 | Pêgo, M. D. M. F., Corazza, P. F. L., Baeder, F. M., Silva, D. F., Albuquerque, A. C. L., Junqueira, J. L. C., & Panzarella, F. K. (2021). Development of the teeth, cervical vertebrae, hand and wrist combined for the estimation of the biological age. *Research, Society and Development, 1*0(3), 1-12. | No mean or/and standard deviation or/and number of subjects values |
| 2021 | Molina, V. G. G., & Schneider, A. R. A. (2021). Relación Entre Edad Cronológica, Ósea Y Dental En Pacientes De 6-11 Años, GUAYAQUIL, 2020. Revista Científica Especialidades Odontológicas UG, 4(2). | No mean or/and standard deviation or/and number of subjects values |
| 2021 | Oncan, E., & Seden, A. K. A. N. (2021).Evaluation Of Maxillary Canine Calcification And Skeletal Maturity Comparing Males And Females Subjects In Different Facial Heights. *Atatürk Üniversitesi Diş Hekimliği Fakültesi Dergisi, 31*(1), 15-21. | No mean or/and standard deviation or/and number of subjects values |
| 2021 | Bhardwaj, A., Kadu, A. A., Jayan, B., Kadu, N., & Kamat, U. R. (2021). Comparative evaluation of cervical vertebrae maturation index and stages of mandibular second molar calcification: A cross-sectional study. *Journal of Dentistry Defence Section, 15*(1), 31-37. | No mean or/and standard deviation or/and number of subjects values |
| 2021 | Ozturk, T., Gumus, H., & Ozturk, G. (2021). Are Dental Maturation, Skeletal Maturation, and Chronological Age Associated With Complete Cleft Lip and Palate?. *The Cleft palate-craniofacial journal, 58*(3), 275–283. https://doi.org/10.1177/1055665620944776 | CVM stage not based on Baccetti’s method (2005) |
| 2021 | Mardiati, E., Komara, I., Halim, H. & Maskoen, A. M. (2021). Determination of pubertal growth plot using hand-wrist and cervical vertebrae maturation indices, dental calcification, peak height velocity, and menarche. *The Open Dentistry Journal, 15*(1). | CVM stage not based on Baccetti’s method (2005) |
| 2021 | Ravera, S., Castroflorio, T., Galati, F., Cugliari, G., Garino, F., Deregibus, A., & Quinzi, V. (2021). Short term dentoskeletal effects of mandibular advancement clear aligners in Class II growing patients. A prospective controlled study according to STROBE Guidelines. *European journal of paediatric dentistry, 22*(2), 119–124. https://doi.org/10.23804/ejpd.2021.22.02.6 | CVM stage not based on Baccetti’s method (2005) |
| 2021 | Nizam, F., Mengal, N., Tareen, M., & Hayat, S. (2021). Skeletal maturation evaluation using mandibular second molar calcification stages. *Pakistan Armed Forces Medical Journal, 71*(3), 983-986. | CVM stage not based on Baccetti’s method (2005) |

**Supplementary Table 2.** Characteristics of included studies.

| **Author et al,. year  (Country)** | **Type of study** | **Records year** | **Funding** | **Sample Size** | **Sample Size, Male/Female** | **Skeletal malocclusion** | **Quality assessment** |
| --- | --- | --- | --- | --- | --- | --- | --- |
| Gu & McNamara, 2007 (1) (USA) | Longitudinal | 1970 | NR | 118 | 41/77 | Class I and III | Moderate |
| Lai, Chang, et al., 2008 (2) (Taiwan) | Cross-sectional | NR | NR | 304 | 0/304 | NR | Moderate |
| Lai et al., 2008 (3) (Taiwan) | Cross-sectional | NR | NR | 709 | 330/379 | NR | Moderate |
| Chen et al., 2010 (4) (Taiwan) | Retrospective and cross-sectional | 1999-2006 | NR | 709 | 330/379 | NR | Moderate |
| Kuc-Michalska & Baccetti, 2010 (5) (Poland) | Cross-sectional | NR | NR | 218 | 125/93 | Class I and III | Low |
| Rozylo-Kalinowska et al., 2011 (6) (Poland) | Retrospective | 2007-2008 | NR | 302 | 134/168 | NR | Moderate |
| Perinetti et al., 2011 (7) (Italy) | Prospective and Cross-sectional | NR | NR | 72 | 27/45 | NR | Moderate |
| Baidas, 2012 (8) (Saudi Arabia) | Cross-sectional | NR | NR | 214 | 104/110 | NR | Moderate |
| Carbonel & Reyes, 2013 (9) (Peru) | Retrospective and cross-sectional | 2011-2012 | NR | 150 | 74/76 | NR | Moderate |
| Hussain et al., 2013 (10) (India) | Cross-sectional | NR | NR | 90 | 47/43 | NR | Moderate |
| Flores et al., 2014 (11) (Peru) | Retrospective | NR | NR | 264 | 109/155 | NR | Moderate |
| Maló et al., 2014 (12) (Portugal) | Cross-sectional | NR | NR | 285 | 114/171 | NR | Moderate |
| Salazar-Lazo et al., 2014 (13) (Peru) | Cross-sectional | NR | NR | 154 | 84/70 | Class I and II | Moderate |
| (Vijayashree et al., 2014 (14) (India) | Cross-sectional | NR | None | 101 | 47/54 | NR | Moderate |
| Giri et al., 2016 (15) (Nepal) | Cross-sectional | NR | NR | 84 | 42/42 | NR | Moderate |
| (Hasan & Abuaffan, 2016 (16) (Sudan) | Retrospective and cross-sectional | 2009-2015 | NR | 112 | 47/65 | NR | Moderate |
| Panainte et al., 2016 (17) (Romania) | Cross-sectional | NR | NR | 221 | 75/146 | NR | Moderate |
| Perinetti et al., 2016 (18) (Italy) | Cross-sectional | 2009-2015 | NR | 320 | 160/160 | NR | Moderate |
| Bedoya Rodríguez et al., 2016 (19) (Colombia) | Prospective | NR | NR | 130 | 58/72 | NR | Moderate |
| (Felemban, 2017 (20) (Saudi Arabia) | NR | NR | NR | 405 | 150/255 | NR | Moderate |
| Kumar, 2017 (21) (India) | Cross-sectional | 2016 | None | 300 | 137/163 | NR | Moderate |
| Mini et al., 2017 (22) (India) | Cross-sectional | 2012-2013 | None | 100 | 46/54 | NR | Moderate |
| Perinetti et al., 2017 (23) (USA) | Longitudinal | NR | NR | 94 | 49/45 | NR | Moderate |
| Wijaya et al., 2017 (24) (Indonesia) | Cross-sectional | NR | NR | 136 | 64/72 | NR | Moderate |
| El-Bakary & Abo  El-Atta, 2018 (25) (Egypt) | Retrospective and cross-sectional | 2013-2014 | NR | 148 | 68/80 | NR | Moderate |
| Flieger et al., 2018 (26) (Poland) | NR | 2014-2016 | None | 180 | 56/124 | NR | Moderate |
| Hosni et al., 2018 (27) (United Kingdom) | NR | 2012-2014 | NR | 22 | 14/8 | NR | Moderate |
| Ghaleb et al., 2019 (28) (Lebanon) | Retrospective and cross-sectional | Till year 2000 | None | 346 | 131/215 | Class I and II | Moderate |
| Mollabashi et al., 2019 (29) (Iran) | Cross-sectional | NR | NR | 600 | 224/376 | NR | Moderate |
| Al-Aunhomi et al., 2020 (30) (Yemen) | Cross-sectional | 2018 | None | 207 | 85/122 | NR | Moderate |
| Banda et al., 2020 (31) (Korea) | Retrospective and cross-sectional | NR | NR | 408 | 181/227 | NR | Moderate |
| Oyonarte et al., 2020 (32) (Canada) | Retrospective and longitudinal | 1952-1970 | NR | 360 | 180/180 | NR | Moderate |
| Mauricio-Vilchez et al., 2020 (33) (Peru) | Retrospective and cross-sectional | NR | NR | 200 | 85/115 | NR | Moderate |
| Dadgar et al., 2021 (34) (Iran) | Cross-sectional | Aug 2019 to Oct 2019 | Self-funded | 224 | 112/112 | NR | Moderate |
| Szemraj-Folmer et al., 2021a (35) (Poland) | Retrospective and cross-sectional | 2008-2018 | Medical University of Gdansk | 41 | 12/29 | Class I | Moderate |
| Szemraj-Folmer et al., 2021b (36) (Poland) | Retrospective and cross-sectional | 2008-2019 | None | 213 | 84/129 | Class I and III | High |
| Moca et al., 2021 (37) (Romania) | Retrospective and cross-sectional | NR | None | 252 | 74/178 | NR | Moderate |
| Oncan & Akan, 2021 (38) (Turkey) | Retrospective and cross-sectional | 2018-2020 | None | 139 | 57/82 | NR | Moderate |
| Schoretsaniti et al., 2021 (39) (Greece) | Retrospective and cross-sectional | NR | None | 474 | 217/257 | NR | Moderate |
| Vuong & Kang, 2021 (40) (USA) | Retrospective and cross-sectional | NR | NR | 420 | 210/210 | NR | Moderate |
| Zahid et al., 2021 (41) (China) | Cross-sectional | 2010-2016 | NR | 32 | 10/22 | NR | Moderate |

NR - Not reported.

**References**

1. Gu Y, McNamara JA. Mandibular Growth Changes and Cervical Vertebral Maturation. The Angle Orthodontist. 2007;77(6):947–53.

2. Lai EH-H, Chang JZ-C, Yao C-CJ, Tsai S-J, Liu J-P, Chen Y-J, et al. Relationship Between Age at Menarche and Skeletal Maturation Stages in Taiwanese Female Orthodontic Patients. Journal of the Formosan Medical Association. 2008;107(7):527–32.

3. Lai EH-H, Liu J-P, Chang JZ-C, Tsai S-J, Yao C-CJ, Chen M-H, et al. Radiographic Assessment of Skeletal Maturation Stages for Orthodontic Patients: Hand-wrist Bones or Cervical Vertebrae? Journal of the Formosan Medical Association. 2008;107(4):316–25.

4. Chen J, Hu H, Guo J, Liu Z, Liu R, Li F, et al. Correlation between dental maturity and cervical vertebral maturity. Oral Surgery, Oral Medicine, Oral Pathology, Oral Radiology, and Endodontology. 2010;110(6):777–83.

5. Kuc-Michalska M, Baccetti T. Duration of the Pubertal Peak in Skeletal Class I and Class III Subjects. The Angle Orthodontist. 2010;80(1):54–7.

6. Rozylo-Kalinowska I, Kolasa-Raczka A, Kalinowski P. Relationship between dental age according to Demirjian and cervical vertebrae maturity in Polish children. The European Journal of Orthodontics. 2011;33(1):75–83.

7. Perinetti G, Baccetti T, Contardo L, Di Lenarda R. Gingival crevicular fluid alkaline phosphatase activity as a non-invasive biomarker of skeletal maturation: GCF ALP activity and skeletal maturation. Orthodontics & Craniofacial Research. 2011;14(1):44–50.

8. Baidas L. Correlation between cervical vertebrae morphology and chronological age in Saudi adolescents. King Saud University Journal of Dental Sciences. 2012;3(1):21–6.

9. Carbonel C, Reyes W. Edad promedio de aparición de los estadios de maduración esquelética de las vértebras cervicales con el Método de Hassel y Farman y Baccetti. Revista dental de Chile. 2013;19–23.

10. Hussain MZ, Talapaneni AK, Prasad M, Krishnan R. Serum PTHrP level as a biomarker in assessing skeletal maturation during circumpubertal development. American Journal of Orthodontics and Dentofacial Orthopedics. 2013;143(4):515–21.

11. Flores SA, Raffo FSE, Duran CL. Correlación entre la maduración esquelética vertebral y el desarrollo dentario de la segunda molar inferior permanente. Ortodoncia Ciencia & Arte. 2014;33–46.

12. Maló L, Lima S, Teixeira V, Canova F, Alves S. Maturação esquelética numa população portuguesa – comparação entre maturação da mão e punho e vértebras cervicais. Revista Portuguesa de Estomatologia, Medicina Dentária e Cirurgia Maxilofacial. 2014;55(2):102–9.

13. Salazar-Lazo R, Arriola-Guillén LE, Flores-Mir C. Duration of the peak of adolescents growth spurt in class I and II malocclusion subjects using a cervical vertebrae maturation analysis. Acta Odontol Latinoam. 2014;27:6.

14. Vijayashree U, Naik V, Pai V. Second molar calcification stages to evaluate skeletal maturation: A cross-sectional radiographic study. APOS Trends Orthod. 2014;4(6):156.

15. Giri J, Shrestha BK, Yadav R, Ghimire TR. Assessment of skeletal maturation with permanent mandibular second molar calcification stages among a group of Nepalese orthodontic patients. CCIDE. 2016;57.

16. Hasan BM, Abuaffan AH. Correlation between Chronological Age, Dental Age and Skeletal Maturity in a sample of Sudanese Children. 2016;10.

17. Panainte I, Pop SI, Mártha K. Correlation Among Chronological Age, Dental Age and Cervical Vertebrae Maturity in Romanian Subjects. The Medical-Surgical Journal. 2016;120(3):700–10.

18. Perinetti G, Rosso L, Riatti R, Contardo L. Sagittal and Vertical Craniofacial Growth Pattern and Timing of Circumpubertal Skeletal Maturation: A Multiple Regression Study. BioMed Research International. 2016;2016:1–7.

19. Bedoya Rodríguez A, Osorio Patiño JC, Tamayo Cardona JA. Edad cronológica y maduración ósea cervical en niños y adolescentes. Revista Cubana de Estomatología. 2016;53(1):43–53.

20. Felemban NH. Third Molar Maturation Stages in Correlation with Cervical Vertebra Maturation Stages in a Saudi Sample. ADOH. 2017;5(5).

21. Kumar S. Correlation between Maxillary Canine Calcification and Skeletal Maturation. JCDR [Internet]. 2017.

22. Mini M, Thomas V, Bose T. Correlation between Dental Maturity by Demirjian Method and Skeletal Maturity by Cervical Vertebral Maturity Method using Panoramic Radiograph and Lateral Cephalogram. J Indian Acad Oral Med Radiol. 2017;29(4):362.

23. Perinetti G, Bianchet A, Franchi L, Contardo L. Cervical vertebral maturation: An objective and transparent code staging system applied to a 6-year longitudinal investigation. American Journal of Orthodontics and Dentofacial Orthopedics. 2017;151(5):898–906.

24. Wijaya H, Kusdhany LS, Redjeki S, Soegiharto BM. The salivary bone spesific alkaline phosphatase in relation to puberal growth phase in indonesian children. Asian J Pharm Clin Res. 2017;10(5):389.

25. El-Bakary A, Abo El-Atta H. Skeletal Maturation Using Cervical Vertebrae Versus Dental Age For Age Estimation. Mansoura Journal of Forensic Medicine and Clinical Toxicology. 2018;26(1):13–21.

26. Flieger R, Matys J, Dominiak M. The best time for orthodontic treatment for Polish childrenbased on skeletal age analysis in accordance to refundpolicy of the Polish National Health Fund (NFZ). Adv Clin Exp Med. 2018;27(10):1377–82.

27. Hosni S, Burnside G, Watkinson S, Harrison JE. Comparison of statural height growth velocity at different cervical vertebral maturation stages. American Journal of Orthodontics and Dentofacial Orthopedics. 2018;154(4):545–53.

28. Ghaleb H, Akl R, Khoury E, Ghoubril J. Estimation and Comparison of the Duration of the Pubertal Peak in Skeletal Class II and Class I Subjects Using the Cervical Vertebrae Maturation Index Method. J Contemp Dent Pract. 2019;20(9):1095–101.

29. Mollabashi V, Yousefi F, Gharebabaei L, Amini P. The relation between dental age and cervical vertebral maturation in orthodontic patients aged 8 to 16 years: A cross-sectional study. International Orthodontics. 2019;17(4):710–8.

30. Al-Aunhomi A, Aldhorae K, Ishaq R, Al-Labani M, Al-Maweri S, Al-Ashtal A. Relationship between cervical vertebral maturation and dental development in a sample of yemeni children and adolescents. J Oral Res. 2020;9(1):7–13.

31. Banda TR, Komuravelli AK, Balla SB, Korrai BR, Alluri K, Kondapaneni J, et al. Discriminatory ability of cervical vertebral maturation stages in predicting attainment of the legal age threshold of 14 years: A pilot study using lateral cephalograms. Imaging Sci Dent. 2020;50(3):209–16.

32. Oyonarte R, Sánchez-Ugarte F, Montt J, Cisternas A, Morales-Huber R, Ramirez-Lobos V, et al. Diagnostic assessment of tooth maturation of the mandibular second molars as a skeletal maturation indicator: A retrospective longitudinal study. American Journal of Orthodontics and Dentofacial Orthopedics. 2020;158(3):383–90.

33. Mauricio-Vilchez C, Mauricio F, Vilchez L, Cadenillas A, Medina J, Mayta-Tovalino F. Radiographic Correlation of Skeletal Maturation Using the Stages of Dental Calcification in a Peruvian Population. Scientifica. 2020; 1–6.

34. Dadgar S, Hadian H, Ghobadi M, Sobouti F, Rakhshan V. Correlations among chronological age, cervical vertebral maturation index, and Demirjian developmental stage of the maxillary and mandibular canines and second molars. Surg Radiol Anat. 2021;43(1):131–43.

35. Szemraj-Folmer A, Wojtaszek-Słomińska A, Racka-Pilszak B, Kuc-Michalska M. Assessment of the duration of the pubertal growth spurt in patients with skeletal open bite: A cross-sectional study. J Orofac Orthop. 2021;82(2):92–8.

36. Szemraj-Folmer A, Wojtaszek-Słomińska A, Racka-Pilszak B, Kuc-Michalska M. Duration of the pubertal growth spurt in patients with increased craniofacial growth component in sagittal and vertical planes—retrospective and cross-sectional study. Clin Oral Invest. 2021;25(8):4907–14.

37. Moca AE, Vaida LL, Moca RT, Țuțuianu AV, Bochiș CF, Bochiș SA, et al. Chronological Age in Different Bone Development Stages: A Retrospective Comparative Study. Children. 2021;8(2):142.

38. Oncan E, Akan S. Assessment of the Relationship between Skeletal Maturity and the Calcifications Stages of Permanent Canines and Second Premolars. Turk J Orthod. 2021;34(1):31–8.

39. Schoretsaniti L, Mitsea A, Karayianni K, Sifakakis I. Cervical Vertebral Maturation Method: Reproducibility and Efficiency of Chronological Age Estimation. Applied Sciences. 2021;11(7):3160.

40. Vuong L, Kang H-K. A cross-sectional retrospective study of normal changes in the pharyngeal airway volume in white children with different skeletal patterns. Part 2: Cervical vertebral maturation method and hyoid bone. American Journal of Orthodontics and Dentofacial Orthopedics. 2021;159(4):e377–88.

41. Zahid D, Zaib, Nik, Yazid, Dalila. Correlation of Chronological Age and Skeletal Maturity in Chinese Patients: A Cross-Sectional Study. Academy of Sciences Malaysia. 2021;14(1):162–8.

**Supplementary Table 3.** Quality assessment of Selected Full Text Article.

| **Author et al., Year** | **Item 1** | **Item 2** | **Item 3** | **Item 4** | **Item 5** | **Item 6** | **Item 7** | **Item 8** | **Item 9** | **Item 10** | **Item 11** | **Item 12** | **Total** | **Quality assessment** |
| --- | --- | --- | --- | --- | --- | --- | --- | --- | --- | --- | --- | --- | --- | --- |
| Gu & McNamara, 2007 | Yes | Yes | No | Yes | No | Yes | Yes | No | Yes | Yes | Yes | No | 8 | Moderate |
| Lai, Chang, et al., 2008 | Yes | Yes | No | Yes | Yes | No | Yes | Yes | Yes | Yes | Yes | No | 9 | Moderate |
| Lai, Liu, et al., 2008 | Yes | Yes | No | Yes | Yes | No | Yes | No | Yes | Yes | Yes | No | 8 | Moderate |
| Chen et al., 2010 | Yes | Yes | No | Yes | Yes | No | Yes | No | Yes | Yes | Yes | No | 8 | Moderate |
| Rozylo-Kalinowska et al., 2011 | Yes | Yes | No | Yes | No | Yes | No | No | Yes | Yes | Yes | No | 7 | Moderate |
| Kuc-Michalska & Baccetti, 2010 | Yes | Yes | No | No | Yes | No | No | No | Yes | Yes | Yes | No | 6 | Low |
| Perinetti et al., 2011 | Yes | Yes | No | Yes | Yes | No | No | Yes | Yes | Yes | Yes | No | 8 | Moderate |
| Baidas, 2012 | Yes | Yes | No | Yes | Yes | Yes | Yes | No | Yes | Yes | Yes | Yes | 10 | Moderate |
| Carbonel & Reyes, 2013 | Yes | Yes | No | Yes | Yes | No | Yes | No | Yes | Yes | No | No | 7 | Moderate |
| Hussain et al., 2013 | Yes | Yes | No | Yes | Yes | No | Yes | No | Yes | Yes | Yes | No | 8 | Moderate |
| Flores et al., 2014 | Yes | Yes | No | Yes | Yes | No | Yes | No | Yes | Yes | No | No | 7 | Moderate |
| Maló et al., 2014 | Yes | Yes | No | Yes | Yes | No | Yes | Yes | Yes | Yes | Yes | No | 9 | Moderate |
| Salazar-Lazo et al., 2014 | Yes | Yes | Yes | No | Yes | Yes | Yes | No | Yes | Yes | Yes | No | 9 | Moderate |
| Vijayashree et al., 2014 | Yes | Yes | No | No | Yes | No | No | No | Yes | Yes | Yes | Yes | 7 | Moderate |
| Giri et al., 2016 | Yes | Yes | Yes | Yes | Yes | Yes | Yes | No | Yes | Yes | Yes | No | 10 | Moderate |
| Hasan & Abuaffan, 2016 | Yes | Yes | No | Yes | No | No | Yes | No | Yes | Yes | Yes | Yes | 8 | Moderate |
| Panainte et al., 2016 | Yes | Yes | No | Yes | Yes | Yes | No | No | No | Yes | Yes | Yes | 8 | Moderate |
| Perinetti et al., 2016 | Yes | Yes | No | Yes | Yes | Yes | Yes | No | Yes | Yes | Yes | No | 9 | Moderate |
| Bedoya Rodríguez et al., 2016 | Yes | Yes | No | Yes | Yes | No | No | No | Yes | Yes | Yes | Yes | 8 | Moderate |
| Felemban, 2017 | Yes | Yes | No | Yes | Yes | No | No | No | Yes | Yes | Yes | Yes | 8 | Moderate |
| Kumar, 2017 | Yes | Yes | Yes | Yes | Yes | No | Yes | No | Yes | Yes | Yes | No | 9 | Moderate |
| Mini et al., 2017 | Yes | Yes | No | No | Yes | Yes | No | No | Yes | Yes | Yes | No | 7 | Moderate |
| Perinetti et al., 2017 | Yes | Yes | No | Yes | Yes | Yes | No | No | Yes | Yes | Yes | No | 8 | Moderate |
| Wijaya et al., 2017 | Yes | Yes | No | Yes | Yes | No | No | Yes | Yes | Yes | Yes | Yes | 9 | Moderate |
| El-Bakary & Abo El-Atta, 2018 | Yes | Yes | No | Yes | Yes | No | No | No | Yes | Yes | Yes | Yes | 8 | Moderate |
| Flieger et al., 2018 | Yes | Yes | No | Yes | Yes | No | No | No | Yes | Yes | Yes | No | 7 | Moderate |
| Hosni et al., 2018 | Yes | Yes | No | Yes | Yes | No | Yes | Yes | Yes | Yes | No | Yes | 9 | Moderate |
| Ghaleb et al., 2019 | Yes | Yes | Yes | Yes | Yes | No | Yes | No | Yes | Yes | Yes | Yes | 10 | Moderate |
| Mollabashi, 2019 | Yes | Yes | Yes | Yes | Yes | No | Yes | No | Yes | Yes | No | No | 8 | Moderate |
| Al-Aunhomi et al., 2020 | Yes | Yes | No | Yes | Yes | No | Yes | No | Yes | Yes | Yes | No | 8 | Moderate |
| Banda et al., 2020 | Yes | Yes | No | Yes | Yes | No | Yes | Yes | Yes | Yes | Yes | Yes | 10 | Moderate |
| Oyonarte et al., 2020 | Yes | Yes | Yes | Yes | Yes | No | No | No | Yes | Yes | Yes | No | 8 | Moderate |
| Mauricio-Vilchez et al., 2020 | Yes | Yes | Yes | Yes | Yes | Yes | Yes | No | Yes | No | Yes | No | 9 | Moderate |
| Dadgar et al., 2021 | No | No | Yes | Yes | Yes | Yes | No | No | Yes | Yes | Yes | No | 7 | Moderate |
| [Szemraj-](https://www.zotero.org/google-docs/?broken=jZIlo6)  Folmer et al., 2021 | Yes | Yes | Yes | Yes | Yes | Yes | Yes | No | Yes | Yes | Yes | Yes | 11 | High |
| [Szemraj-](https://www.zotero.org/google-docs/?broken=jZIlo6)  Folmer et al., 2021 | Yes | Yes | No | Yes | Yes | Yes | Yes | No | Yes | Yes | Yes | Yes | 10 | Moderate |
| Moca et al., 2021 | Yes | Yes | No | Yes | Yes | No | No | No | Yes | Yes | Yes | Yes | 8 | Moderate |
| Oncan & Akan, 2021 | Yes | Yes | No | Yes | Yes | Yes | No | No | Yes | No | Yes | No | 7 | Moderate |
| Schoretsaniti et al., 2021 | Yes | Yes | No | Yes | Yes | Yes | Yes | No | Yes | Yes | Yes | Yes | 10 | Moderate |
| Vuong & Kang, 2021 | Yes | Yes | No | Yes | Yes | No | No | No | Yes | Yes | Yes | No | 7 | Moderate |
| Zahid et al., 2021 | Yes | Yes | No | Yes | Yes | Yes | Yes | No | Yes | Yes | Yes | No | 9 | Moderate |

Item1. Are the objectives clearly formulated?; Item 2. Are there key elements of study design early in the paper?; Item 3. Was the sample size calculated?; Item 4. Does the study report demographic characteristics of the study population?; Item 5. Were the sample selection criteria clearly described?; Item 6. Does the study describe specifications of material and methods involved including how and when measurements were taken?; Item 7. Was there a reliability assessment, with adequate level of agreement intraexaminer or/and interexaminer?; Item 8. Were there blinding measurements?; Item 9. Does the study give details of methods of assessment (measurements) for each variable of interest?; Item 10. Was there a complete and adequate reporting of results, with self-explanatory tables and figures?; Item 11. Was there a statistical analysis appropriate for data?; Item 12. Was the P-value stated or confidence intervals provided?

**Supplementary Table 4.** Grading of Recommendations Assessment, Development and Evaluation analyses.

| **CVM** | **Study design** | **Risk of bias** | **Inconsistency** | **Indirectness** | **Imprecision** | **Publication bias** |
| --- | --- | --- | --- | --- | --- | --- |
| CS-1 | Observational studies (serious) | Not serious | Serious* | Not serious | Not serious | Not serious |
| CS-2 | Observational studies (serious) | Not serious | Serious* | Not serious | Not serious | Not serious |
| CS-3 | Observational studies (serious) | Not serious | Serious* | Not serious | Not serious | Not serious |
| CS-4 | Observational studies (serious) | Not serious | Serious* | Not serious | Not serious | Not serious |
| CS-5 | Observational studies (serious) | Not serious | Serious* | Not serious | Not serious | Not serious |
| CS-6 | Observational studies (serious) | Not serious | Serious* | Not serious | Not serious | Not serious |

*Downgraded for serious inconsistency: even considering the large sample sizes and the use of digital, some degree of heterogeneity is still perceptible.
